# Nanoscale Topography Dictates Residue Hydropathy in Proteins

**DOI:** 10.1021/acs.langmuir.4c02142

**Published:** 2024-10-11

**Authors:** Jingjing Ji, Advait D. Shukla, Ratnakshi Mandal, Wafiq Ibsan Khondkar, Catilin R. Mehl, Arindam Chakraborty, Shikha Nangia

**Affiliations:** † Department of Biomedical and Chemical Engineering, Syracuse University, Syracuse, New York 13244, United States; ‡ Department of Electrical Engineering and Computer Science, Syracuse University, Syracuse, New York 13244, United States; § Department of Biology, Syracuse University, Syracuse, New York 13244, United States; ∥ Department of Chemistry, Syracuse University, Syracuse, New York 13244, United States

## Abstract

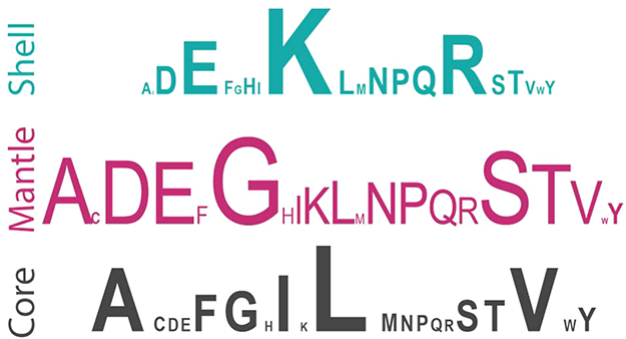

Proteins exhibit diverse structures, including pockets, cavities, channels, and bumps, which are crucial in determining their functions. This diversity in topography also introduces significant chemical heterogeneity, with polar and charged domains often juxtaposed with nonpolar domains in proximity. Consequently, accurately assessing the hydropathy of amino acid residues within the intricate nanoscale topology of proteins is essential. This study presents quantitative hydropathy data for 277,877 amino acid residues, computed using the Protocol for Assigning a Residue’s Character on a Hydropathy (PARCH) scale. Leveraging this data set comprising 1000 structurally diverse proteins sourced from the Protein Data Bank, we examined residues situated in various nanoscale environments and analyzed hydropathy in relation to protein topography. Our findings indicate that the hydropathy of a residue is intricately linked to both its individual characteristics and the geometric features of its neighboring residues in response to water. Changes in the number and chemical identity of the neighbors, as well as the nanoscale topography surrounding a residue, are mirrored in its hydropathy profile. Our calculations reveal the intricate interplay of hydrophilic, hydroneutral, and hydrophobic residues distributed across the surface and core of proteins. Notably, we observe that protein surfaces can be ten times more hydrophilic than their cores.

## Introduction

Structured proteins have precise nanoscale topography, with features such as pockets, channels, crevices, and bumps.^1−4^ The chemical environment of a topographical feature, for example, a pocket, is a function of the resident amino acids.^5^

Depending on the residues lining the pocket wall, the pocket can be hydrophilic or hydrophobic. Moreover, neighboring pockets within a few nanometers may have opposite hydropathies. Similarly, intrinsically disordered proteins can adopt various conformations with variable hydropathies. Therefore, quantifying the hydropathies of residues in protein is critical for understanding protein–protein interactions, protein folding, and protein–ligand binding.

However, quantifying a residue’s hydropathy in the context of its topography has been challenging.^6−9^ Numerous hydropathy scales have used different features of proteins to assign a numerical value to the amino acid residues that best describe their character.^1,5,10−21^ A recent approach called the Protocol for Assigning a Residue’s Character on a Hydropathy (PARCH) scale can accurately and affordably measure the hydropathy of a residue based on this local nanoscale environment.^22^ The method evaluates the collective response of the water molecules in the protein’s first hydration shell to increasing temperatures. Each residue is assigned a value between 0 and 10 based on how readily it loses or retains water. A residue on a protein’s surface, exposed to water, has a different parch value than when the same residue is buried inside a protein’s fold. The parch scale, therefore, permits the comparison of residues in complex topological environments.

**Figure 1:**
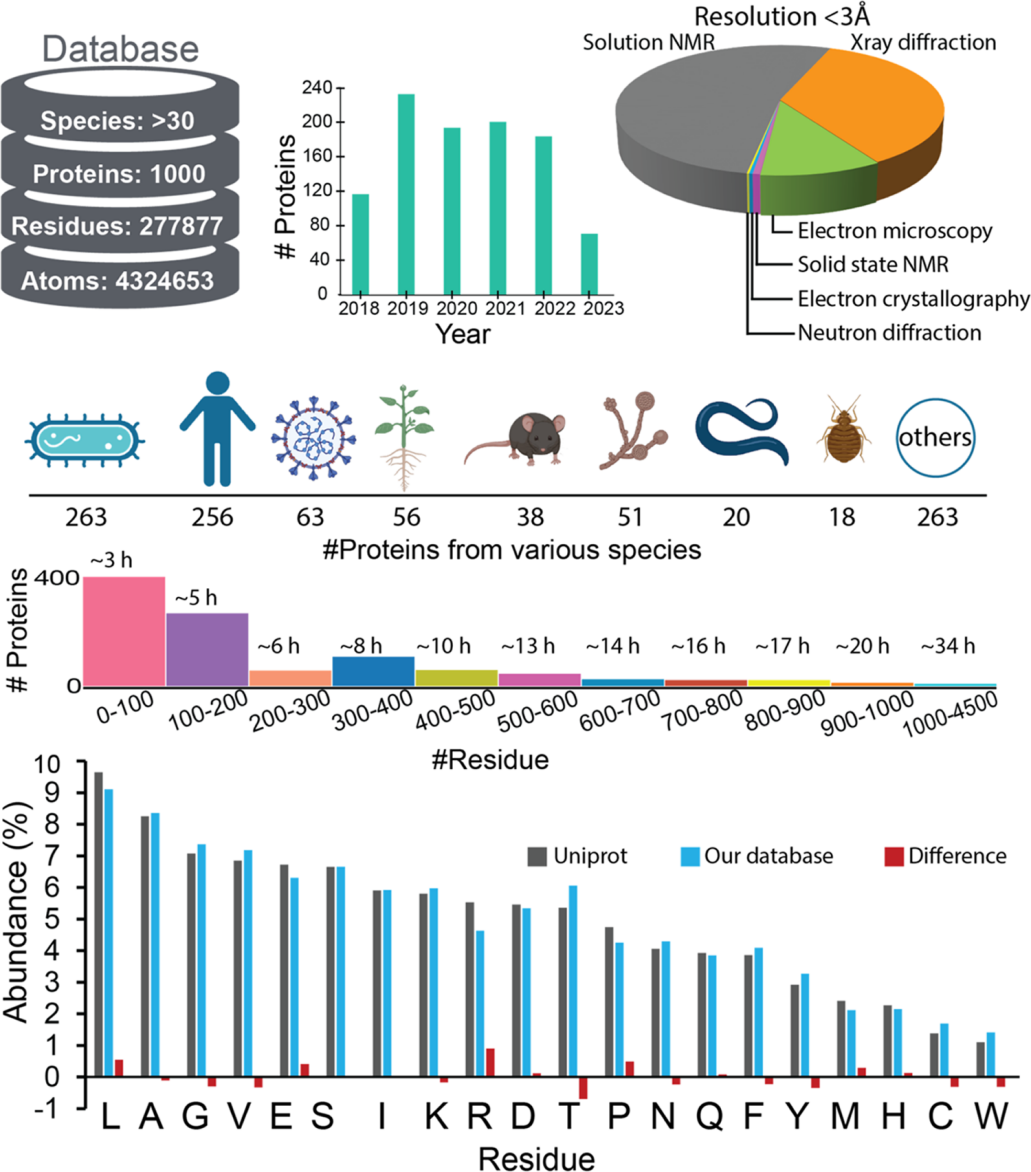
Overview of the Protein Data set. The figure provides a comprehensive breakdown of the data set. The top-left panel presents the count of species, proteins, amino acid residues, and atoms included in our database. The top-center panel illustrates the distribution of protein release years to the PDB (https://www.rcsb.org) databank. The top-right panel highlights the experimental methods used to determine protein structures. In the middle section, the number of proteins across various species is shown alongside a histogram depicting protein counts binned by residue number. The bottom section compares the percent abundance of the 20 amino acid residues in the UniProt release (2023–03) with those in our database, with the difference in abundance remaining below 1% for all residues.

Here, we examine 1000 proteins to decode the relationship between a protein’s topography and hydropathy. To ensure the data set encompasses proteins with diverse topographies, they were sourced from the Protein Data Bank (Table S1) and exhibit less than 30% sequence identity.^23^ Furthermore, the selected structures were determined using experimental techniques such as solution and solid nuclear magnetic diffraction (NMR), X-ray diffraction (XRD), electron microscopy, and neutron diffraction (Figure 1). The average resolution of the protein structures is less than 3 Å. The proteins span multiple species and perform diverse functions such as enzymes, binding proteins, toxins, transport proteins, membrane proteins, chaperons, hormones, and others (Figure S1). The 1000 protein data comprise 277,877 residues. The repartition of the data set shows proteins of variable sizes; the smallest proteins are ten residue peptides, and the largest protein has 4428 residues. Compared to our data set, the UniProtKB/Swiss-Prot^24^ contains 569793 sequence entries, curated from 293323 unique references and comprising 206004162 amino acids. The residue abundance order in the UniProtKB/Swiss-Prot sequence data set shows that L and W are the most and the least abundant residues, respectively, which matches the abundance observed in our data set. The difference between the two data sets is less than ±1%, confirming that our data set represents the amino acid distribution of the known proteins.

Next, we evaluated the parch values of the residues in the database. To map parch values to the topography, we recorded additional attributes, such as the number and identity of neighboring residues, location of the residues on the protein surface versus core, frequency of specific neighbors, number of contacting water molecules, and the ratio of amino acid neighbors to contacting waters. We observed that the parch value of a residue is influenced by three factors: (i) the number of neighboring residues, (ii) the chemical identity of the neighbors, and (ii) the number of surrounding water molecules. Although the role of these factors is intuitive in protein’s conformational stability, folding, and function, their implication in surface topography and hydropathy has not been quantified and reported in the literature.

Moreover, the parch method is computationally affordable, requiring only a few hours to run the calculations in triplicate—the largest protein in our database with ∼5000 residues required ∼34 h. The affordability of the methods allowed us to generate an unparalleled hydropathy database of 277,877 residues in 1000 experimentally reported protein structures. To our knowledge, no other protein database offers hydropathy values of residues based on the nanoscale structure surrounding the amino acid residues. This knowledge has enabled us to predict amino acid mutations that can enhance or prevent ligand binding, alter enzyme activity, and missense mutations. We expect this database to grow and for the scientific community to incorporate hydropathy into protein engineering and design workflows.

In the results section, we highlight the key findings of this work and show that the hydropathy of a residue is a collective property of the neighborhood in which the focal residue resides. Therefore, residues have different hydropathy or parch values based on their topographic neighbors. A sole amino acid or its bonded partners are insufficient to provide a complete picture of the hydropathy of the protein. We provide a systematic way of identifying amino acid neighborhoods and provide hydropathy with nanoscale precision. The entire approach is computationally affordable and has been shown to corroborate experimental data.

## Approach

**Figure 2:**
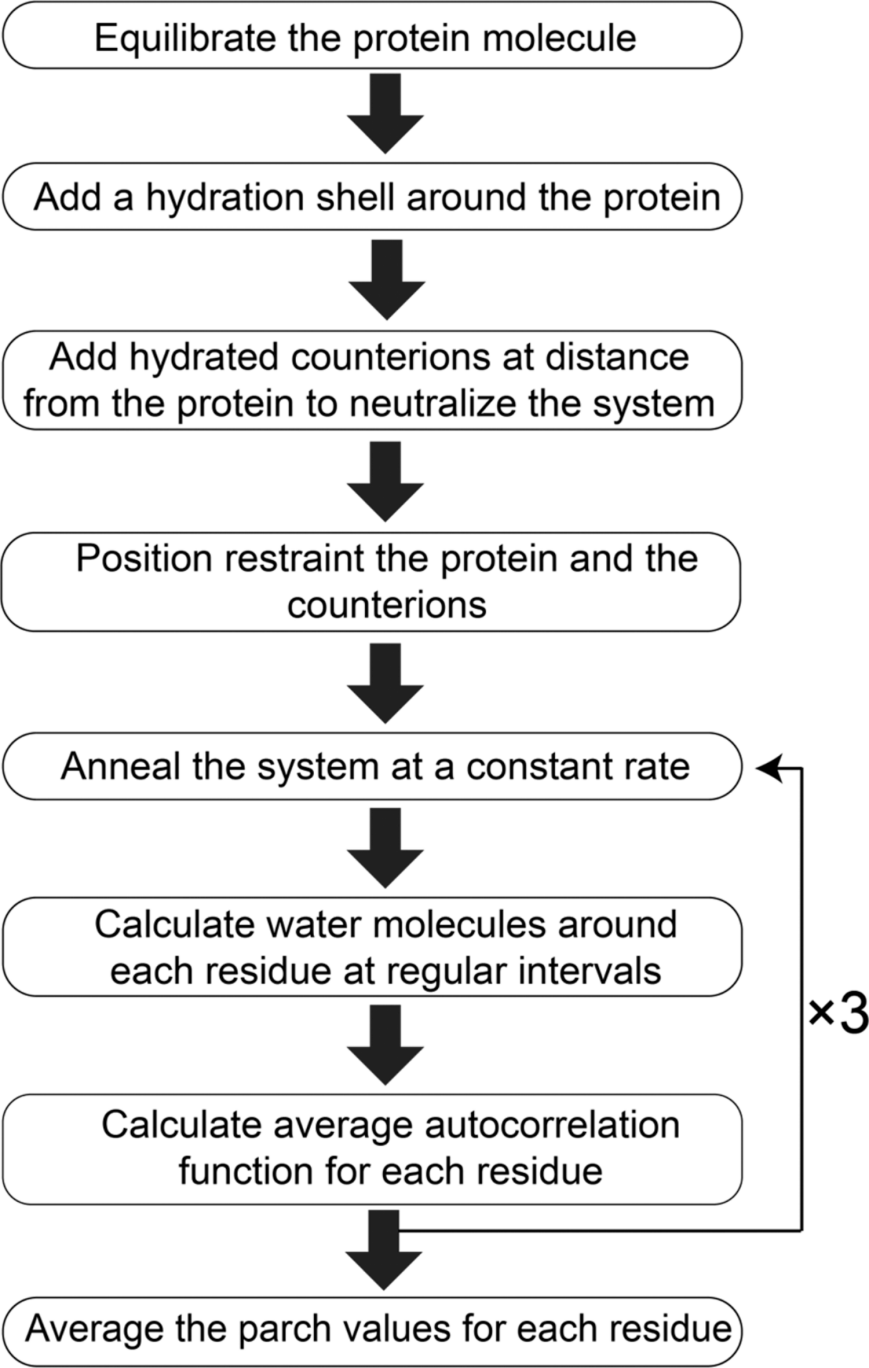
The parch scale calculation workflow.


Our approach to compute the hydropathy of each residue in a protein is based on tracking the number of water contacts during the annealing process. The foundational elements of our approach were reported earlier,^22^ but here we include the major components of the parch calculation workflow (Figure 2). The process begins by placing the equilibrated atomistic structure of the protein enveloped in a shell of water of uniform thickness in the center of an empty cubic simulation box. The system is neutralized by hydrated counterions placed at a fixed distance from the protein surface to prevent these ions from interacting with the protein and the hydration shell. The system is then annealed at a constant rate to determine the loss or retention of water from each residue. During this process, the protein is position-restrained to prevent conformational changes in the protein’s structure. The ions are also position-restrained to prevent ions from interfering with the protein and the water molecules. As the temperature increases, water molecules progressively evaporate from the protein surface. The number of water molecules contacting each residue is recorded at regular time intervals during the annealing process. Finally, for each residue, *i*, a time-averaged autocorrelation function, *C̅*_*i*_ of the water molecules is computed. The *C̅*_*i*_ value is then divided by the time-averaged autocorrelation function of a reference zwitterionic lysine (Lys) residue, *C̅*_Lys_, to normalize the parch values. Moreover, normalizing the time-averaged autocorrelation function of a residue against a reference makes the method less dependent on the choice of the force field used for computing the protein–water interactions. The quantity, *C̅*_*i*_/*C̅*_Lys_, has a value between 0 and 1, but to expand our parch values on a broader scale, we multiply the quantity by 10 for parch values to span a 0–10 scale, as shown. The annealing process is performed in triplicate, and the final parch value of each residue is obtained by averaging the three annealing runs. Notably, residues in the protein core that do not contact water have a zero *C̅*_*i*_ values and by definition have 0 parch values. In sum, the hydropathy of amino acid residues is measured on the 0–10 parch scale, where 0 represents the most hydrophobic residue, and 10 is the most hydrophilic residue.

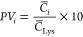


## Results and Discussion

### Hydropathy of an Amino Acid Depends on Its Nanoscale Topography

**Figure 3:**
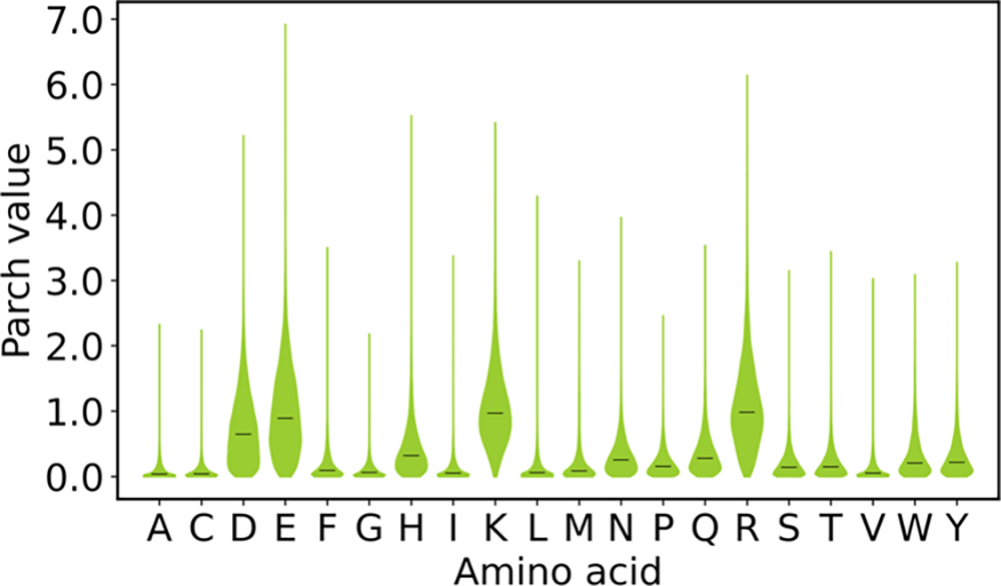
Hydropathy of amino acid residues. Parch value distributions of amino acids were computed for a 277,877-residue data set on the 0–10 parch scale, where 0 and 10 denote hydrophobic and hydrophilic character, respectively. The horizontal line denotes the median parch value for each amino acid.

Our analysis of a 277,877-residue data set revealed a diverse range of hydropathies of amino acids in proteins. Using this large data set, we constructed reliable distributions of parch values for the 20 amino acids through violin plots illustrating the median parch values (Figure 3). Notably, these violin plots have different shapes, widths, tail lengths, and median parch values, emphasizing the variability in amino acid hydropathy. Most hydropathy scales assign a unique value to each amino acid based on specific chemical attributes critical to that particular scale (Table S2). As a result, these scales often differ in identifying the most hydrophobic or hydrophilic amino acids. Interestingly, the median parch values of the residues align with hydrophobic trends reported by several hydropathy scales. One of the more recent scales, the Water Orientation Probability Hydropathy Scale (WOPHS)^8^ uses the orientation of water molecules around a residue to assign three values to each residue that indicate the hydrophilic, hydrophobic, and overall measure of hydropathy. However, WOPHS also assigns fixed values to each amino acid, which remain constant regardless of changes in nanoscale topography.

The parch scale overcomes the limitation of the hydropathy scales because it captures the hydropathic character of amino acids in different nanoscale topographies and assigns a parch value on a 0–10 scale that is context-based. Aspartate can have a parch value anywhere from 0 to 7. Even for the most hydrophobic residue leucine, the parch values vary from 0 to 4.5. This variability in parch values highlights that the chemical identity of a residue alone does not dictate its hydropathy; rather, it is context-based and depends on other factors discussed in later sections of this article. The low median parch values of amino acids leucine, isoleucine, and valine show that these residues are hydrophobic in most contexts, consistent with experimental and computational studies. The WOPHS approach ranks leucine as the most hydrophobic residue.^8^

Our analysis revealed that the median parch values for the charged residues are higher than those of the uncharged residues. Moreover, there are noticeable distinctions in the shapes of violin plots between positively and negatively charged residues. Specifically, negatively charged residues exhibit a higher proportion of zero parch values compared to positively charged residues, evident from the wider violin plots at this value. While all 20 amino acids can possess a zero parch value, lysine, and arginine residues exhibit the lowest proportion of such values. Further investigation unveiled that positively charged residues are more prevalent on the protein surface, correlating with their tendency to exhibit higher parch values rather than lower or zero values. The WOPHS scale ranked the charged residues among their most hydrophilic residues. Our data shows high median parch values for aspartate and other charged amino acid (glutamate, lysine, and arginine) residue. However, like other residues, the charged residues can also have a 0 parch value, emphasizing the significance of context-based hydropathy. Further analysis revealed that these hydropathy shifts are influenced by both the number and identity of neighboring residues.

### Amino Acid Hydropathy Changes Based on Its Location in a Protein: Surface Versus Core

To systematically map the hydropathy of residues to the protein’s topography, we developed a method that divided the residues into three distinct zones: shell, mantle, and core. This classification was based on the count of contacting water molecules and neighboring amino acids within a specified cutoff (Figure S2). Utilizing an unsupervised learning algorithm on a robust data set of 277,877 residues, we established precise criteria for zone assignment, detailed in the Supporting Information. The residues identified in the shell zone are water-exposed and have few contacting residues, while mantle residues show partial exposure to water with comparatively more residue contacts than the shell. In contrast, core residues exhibit minimal to no water contact but display significantly higher residue contacts than the shell. This analysis enabled us to delineate topographical features such as channels, cavities, and bumps within proteins. On average, shell, mantle, and core residues constitute approximately 20, 40, and 40% of structured proteins, respectively, with a few unstructured proteins lacking a discernible core.

**Figure 4:**
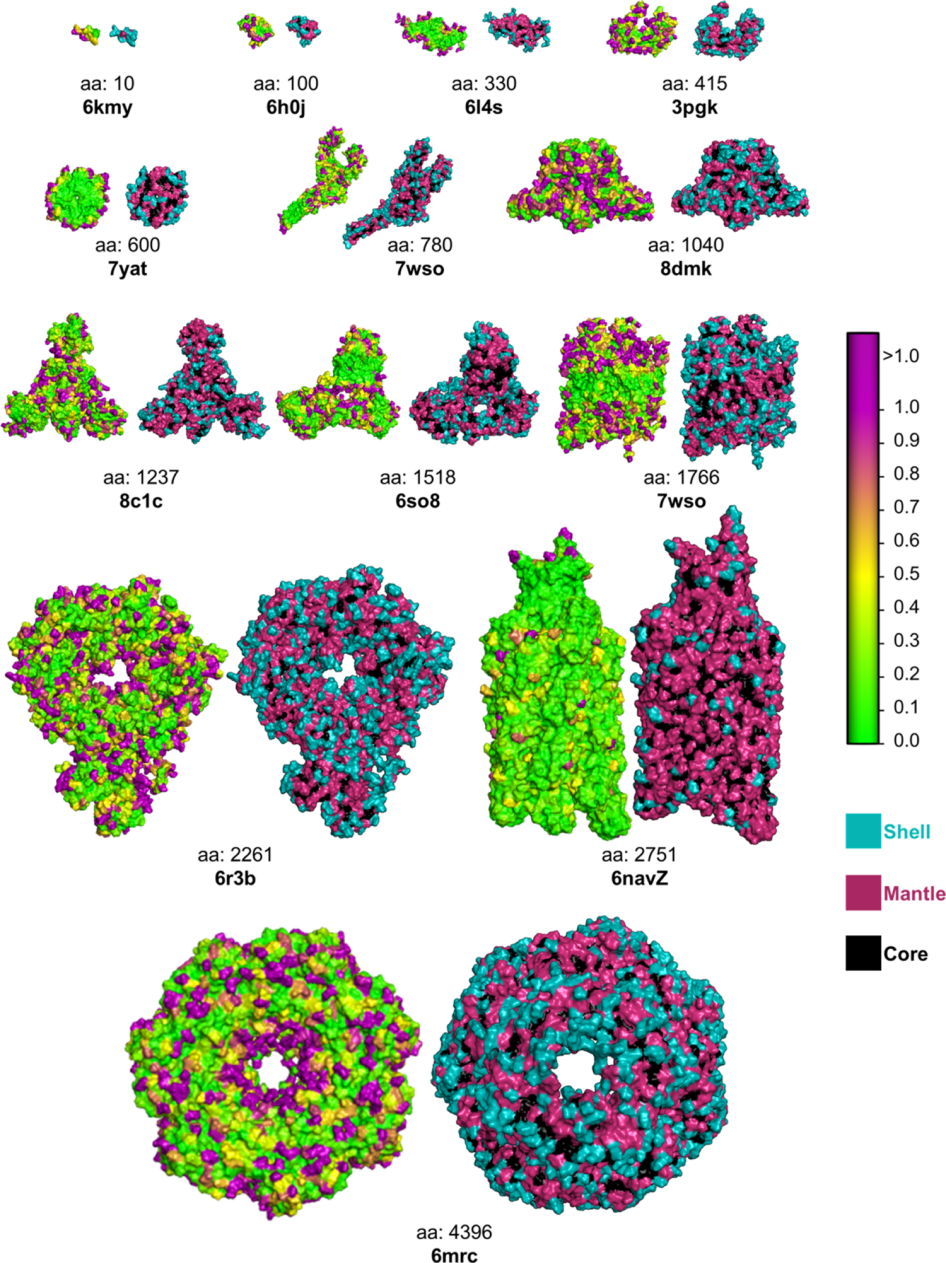
Hydropathy of amino acid residues. Hydropathy of a representative set of proteins of different sizes and shapes. The number of amino acids (aa) and the Protein Data Bank ID is provided for each protein. The left image of each protein shows residues colored based on their parch values on the color scale, where 0 denotes hydrophobic character, and 10 denotes hydrophilic character. The right image of each protein shows the three protein zones—shell, mantle, and core. All proteins, irrespective of shape and size, show hydrophilic, hydrophobic, and hydroneutral residues and have shell, mantle, and core zones.

Dividing each protein into three zones provides a framework to compare different proteins, even if they vastly differ in size or topography. Comparison of small (<200 residues), medium (200–2000 residues), and large (>2000 residues) proteins revealed that all categories exhibit hydrophilic, hydrophobic, and hydroneutral residues regardless of size or shape (Figure 4), reinforcing the importance of evaluating residues within the context of their neighboring environment. These neighboring residues impose unique nanoscale constraints on conformation and steric accessibility, influencing binding affinity with water.

We can compare parch values of shell residues of different proteins and report most hydrophilic or hydrophobic proteins. We can compare proteins with the largest core or the largest mantle. We can show how the hydropathies of protein cores vary among the proteins. For a single protein, we can evaluate the effect of the chemical identity of the residue’s neighbor(s) on the hydropathy in each zone. The combination of parch values and a three-zone description of the protein provides a quantitative measure of hydropathy and a standard scale to study and compare proteins.

**Figure 5:**
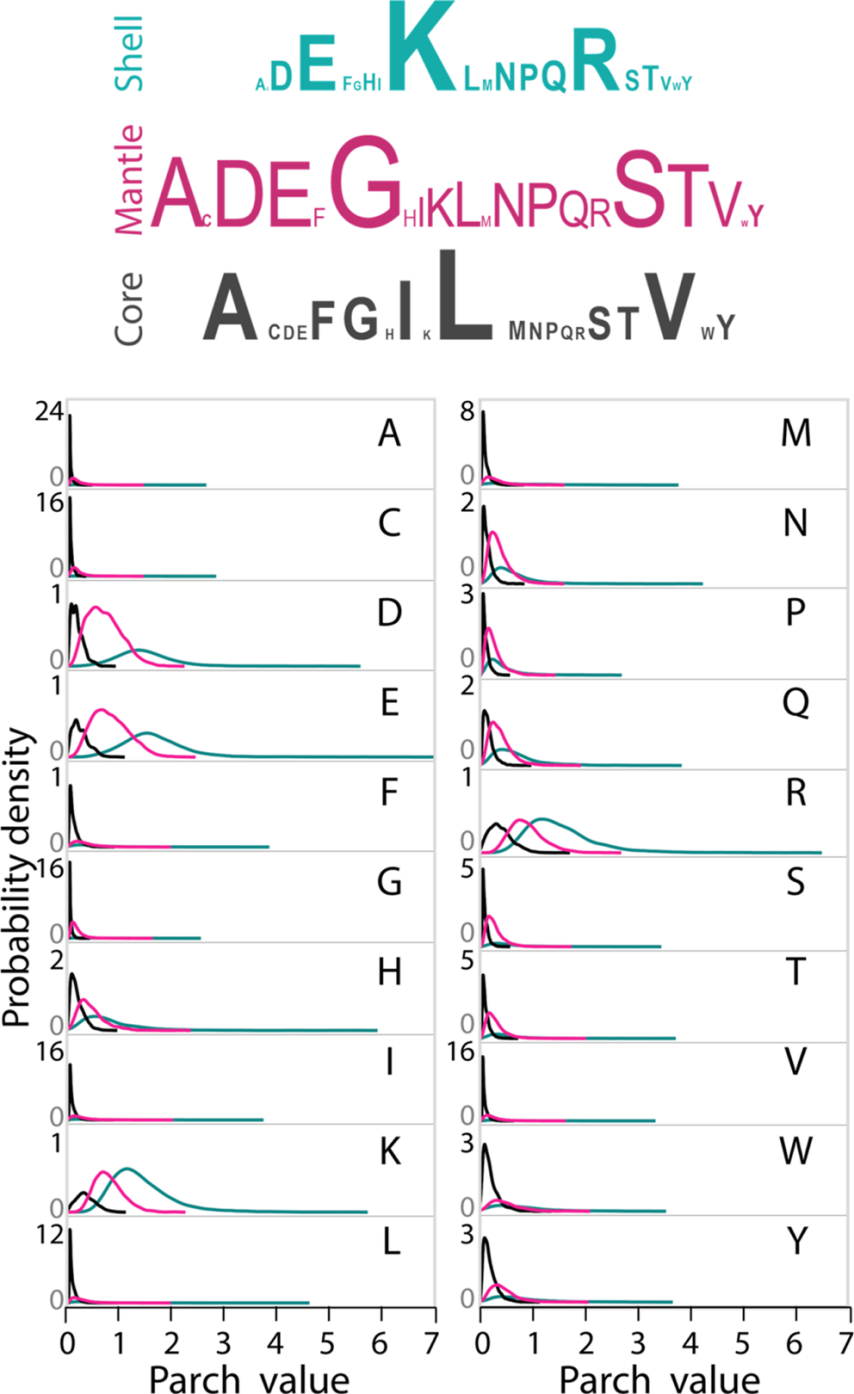
The relative abundance of the 20 amino acids is denoted by the size of letters in the shell (teal), mantle (pink), and core (black) zones (top). The parch value probability distributions of the amino acid residues were generated from 277,877 residues in the protein data set (bottom).

Interestingly, the three zones revealed distinct populations of the amino acid residues (Figure 5). Leucine, the most abundant residue in nature, is not uniformly distributed in a structured protein. Instead, it is most abundant in the protein’s core. Other core abundant residues include valine (V), alanine (A), and isoleucine (I). In contrast, the shell has the highest abundance of charged residues, particularly positively charged lysine (K) and arginine (R), followed by negatively charged glutamic acid (E). The mantle, packed between the core and the shell, has the highest abundance of glycine (G), followed by serine (S), alanine (A), and negatively charged residues aspartic acid (D) and E. Residues with aromatic side chains, such as phenylalanine (F), tyrosine (Y), and tryptophan (W), are more abundant in the core of the structure than in the mantle or shell.

We calculated the average parch values for the three zones within each protein, revealing distinctive hydropathy profiles. The mean and standard deviation of the parch values in the shell, mantle, and core zones are 0.89 ± 0.19, 0.31 ± 0.09, and 0.06 ± 0.03, respectively (Figure S3). These results emphasize the varying degrees of hydrophilicity across protein zones, with the shell being the most hydrophilic, followed by the mantle and the core. The elevated hydrophilicity of the shell can be attributed to the higher abundance of K, R, and E residues and their preferential colocalization with other residues. Conversely, the core, enriched in L, V, and A residues, exhibits a distinct chemical composition compared to the shell. Similarly, the mantle, characterized by G, S, and T residues, differs chemically from both the core and the shell. It is noteworthy that peptides, small proteins, and intrinsically disordered proteins may lack a core.

In proteins with a core, the shell exhibits hydrophilicity levels that are typically an order of magnitude higher than those observed in the core, underscoring the crucial function of the shell in facilitating interactions with other biomolecules, irrespective of protein size. Directly comparing the parch values of protein shells provides a convenient method for assessing their hydrophilicity levels. The average parch values of proteins in our database ranged from 0.27 to 1.61. For reference, Table S3 presents the list of the data set proteins arranged by decreasing parch values of the shell.

Interestingly, by changing the resolution from zones to individual amino acids, we observe that the parch value distribution for each amino acid is also distinct. Figure 5 shows the probability density curves for the parch values for the residues in the shell, mantle, and cores. The residues span a wider range of parch values in the shell than in the mantle or core, indicating a higher influence of local geometry.

The key observations that emerge by comparing parch value distributions of the residues:

- All residues exhibit a wide range of parch values, with their highest values in the shell.
- Charged residues D, E, K, and R have a broader range of parch values than others.
- All residues can have zero parch values, irrespective of their chemical nature. Charged residues K, R, D, and E can cease to be hydrophilic due to the local nanoscale topography in the shell, mantle, and core.
- Residues L, I, V, and A, abundant in the core, have parch values up to 3 or 4 in the shell.
- Most of the residues in the core have parch values of less than 1, with a few exceptions, including K, R, and E.
- Glutamate, though most abundant in the mantle, has its highest parch value of 7 in the shell.
- The parch values of all residues in the mantle lie between 0 and 2.7.

This work unequivocally shows that the amino acid residues do not have fixed hydropathy, unlike other hydropathy scales that classify amino acids as hydrophilic or hydrophobic or assign a single numeric value on a relative hydropathy scale.^9,11,12^ Hydropathy of proteins is far more complex and depends on the residue’s location and topological neighbors.

### Amino Acid Residues Often Colocalize with Preferred Topological Neighbors

The analysis of nearest neighbor interactions among the 20 amino acids unveiled distinct colocalization preferences for each residue. These preferences are the consequence of local topography and could be either bound or proximal neighbors. A heatmap depicting the colocalization preferences for each amino acid’s first closest neighbor illustrates these preferences. Notably, our observations of residue pairs, or dyads, across multiple proteins reveal statistically significant trends, as the data is averaged over a data set comprising 1000 proteins (Figure S4). This colocalization data provides significant insights into the topology and, ultimately, the hydropathy of proteins.

Leucine, the most abundant amino acid residue, demonstrates a propensity for forming dyads, particularly with residues possessing aliphatic side chains—highlighting preferences such as LI, LV, LA, LL, and LF. The prevalence of leucine in these dyads may be attributed in part to its high abundance. However, other aliphatic chain residues, such as valine (V), alanine (A), and isoleucine (I), also exhibit prolific and nonselective tendencies in forming dyads.

Among charged residues, lysine forms abundant dyads with residues like glutamic acid, leucine, alanine, and aspartic acid—KE, KL, KA, KD. Similarly, arginine predominantly forms dyads with glutamic acid—RE. Negatively charged residues, such as aspartic acid and glutamic acid, exhibit dyad formations with lysine and arginine. Overall, we observed that most residues form abundant dyads with leucine, except for cysteine (C), which forms CC dyads.

**Figure 6:**
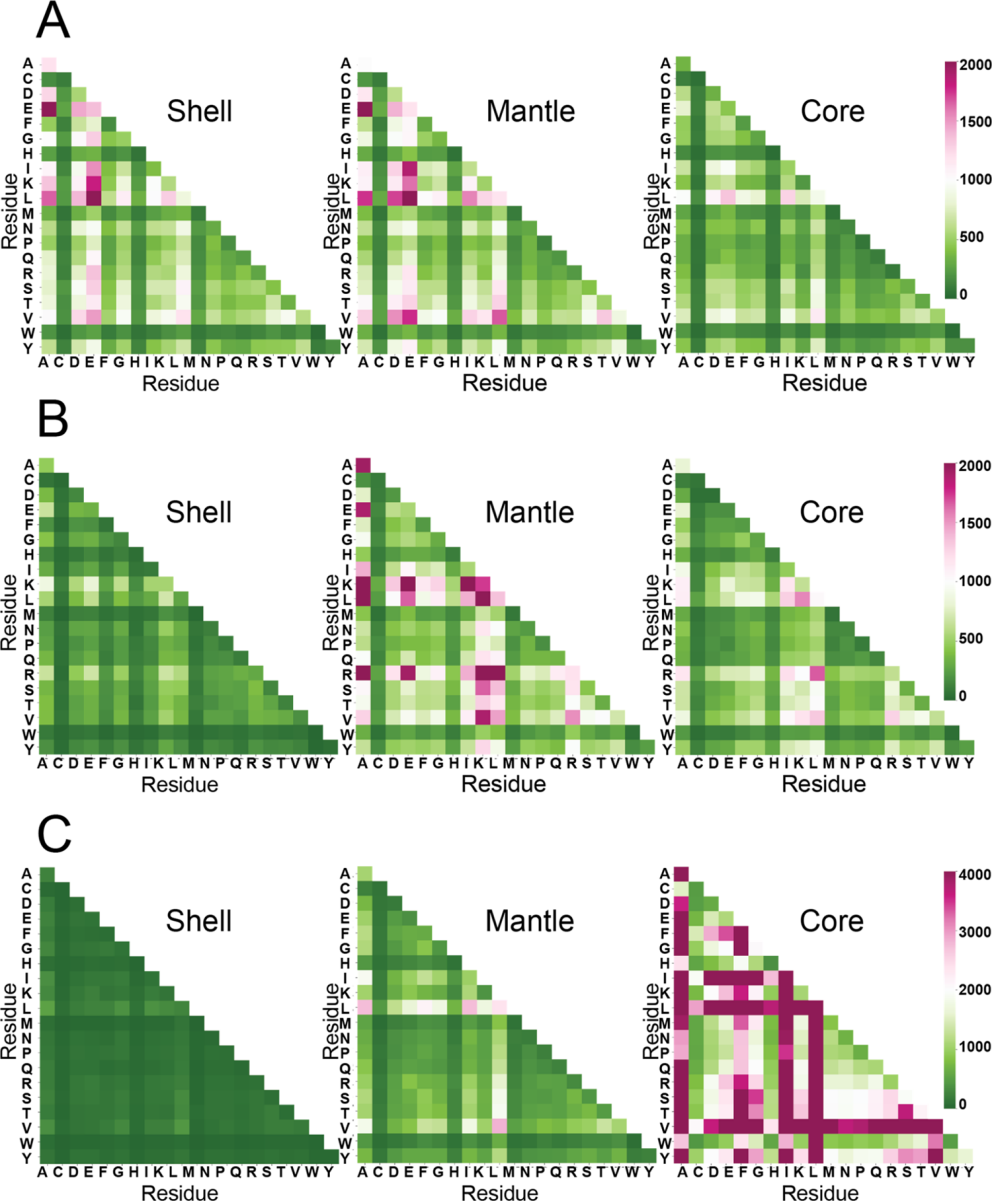
Heat maps of residue triads. (A) KXX (B) EXX, and (C) LXX triad in the shell, mantle, and core. Variable X denotes the 20 amino acid residues shown on the *x* and *y* axes of the heat maps. The color bars are shown on the right for all three triads.

Expanding the topological neighborhoods from dyads to triads revealed that charged residues colocalize with each other or with residues with aliphatic side chains. For example, the most abundant triads of K are extensions of the KE dyad—KEK, KEE, KED, KER, KEL, and KEA. Interestingly, these triads are not uniformly distributed in the protein; they are more abundant in the shell and mantle than the core of proteins (Figure 6A). The negatively charged E forms abundant triads with R, K, L, and A, which preferentially occupy the mantle (Figure 6B). The L residues follow their dyad behavior and colocalize with L, I, V, and A to form abundant triads in the core (Figure 6C). The triads of all remaining residues are provided in the Supporting Information (Figures S5–S21).

The emergent theme from examining the dyads and triads is that each zone defines a colocalization of residues. The distribution of triads in the shell, mantle, and core zones impacts the zonal hydropathies. The shell richer in lysine triads contributes to a higher average parch value than the mantle and the core. In contrast, the core rich in leucine triads is relatively more hydrophobic than the shell and mantle.

### Hydropathy of an Amino Acid Depends on the Chemical Identity of the Neighbors

The parch values of amino acid residues in local nanoscale geometries reveal a strong dependency on the chemical identity of their neighbors, particularly in their interaction with water. To demonstrate this, we examined K triads with various combinations of charged (±) and uncharged (0) neighbors: K±±, K ± 0, and K00. We computed the average parch values of these triads across the shell, mantle, and core zones, reflecting their differing water accessibility.

**Figure 7:**
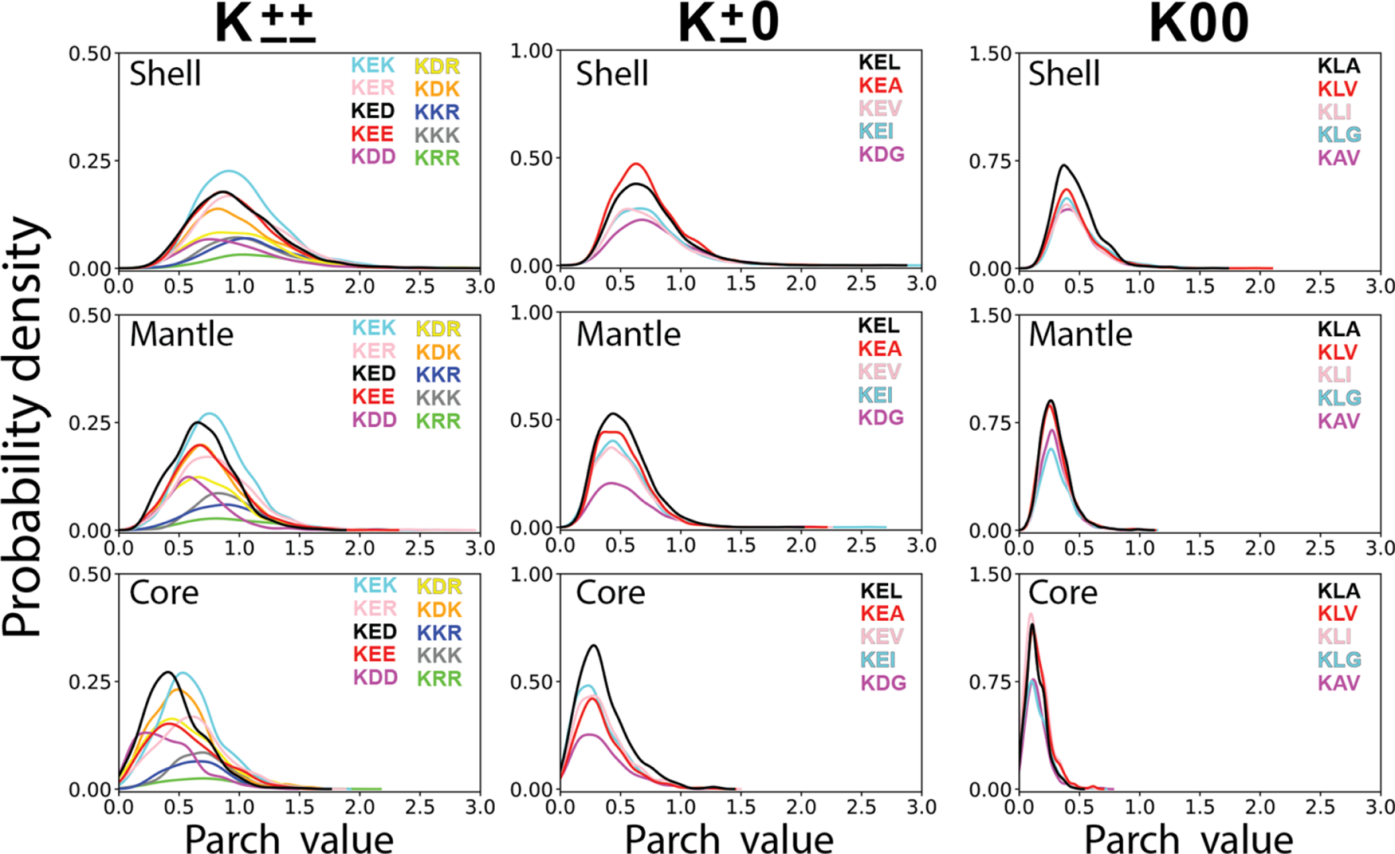
The probability density of parch values of K±±, K ± 0, and K00 triads in the shell, mantle, and core of proteins.

In the protein’s shell, all ten K±± triads (KED, KEE, KER, KEK, KDD, KDR, KDK, KRR, KKR, and KKK) exhibited similar parch value distributions, with the most probable values ranging from 0.9 to 1.0 and a maximum parch value of 3.0 (Figure 7). However, in the mantle and core, the distributions shifted to lower parch values of 0.6–0.7 and 0.4–0.5, respectively. This trend aligns with the observation that the shell is the most hydrophilic, followed by the mantle and core zones. Additionally, analysis of probability density distributions revealed that KEK was the most abundant lysine triad, while KRR was the least abundant across all zones. This suggests that charged triads encompassing both positively and negatively charged residues, such as KED and KER, are preferred over same charge triads like KKK or KKR. This observation underscores the importance of local nanoscale environments containing opposite charges for achieving high water affinity in proteins.

For the K ± 0 triads, we opted for E as the charged residue, given that KE is the most prevalent K dyad, alongside naturally abundant uncharged residues like L, I, A, V, and G. Among the five K ± 0 triads (KEL, KEI, KEA, KEV, and KEG), the most probable parch values were 0.7, 0.5, and 0.3 in the shell, mantle, and core, respectively. This decreasing trend in parch values from the shell to the core aligns with our earlier observations. Notably, the KEA triad predominates in the shell, while as the abundance of L increases away from the protein shell, KEL becomes more prevalent in the mantle and core.

In examining the K00 triads, we investigated combinations of K with aliphatic side chain residues L, I, A, V, and G. Across the five triads (KLA, KLV, KLI, KLG, and KAV), the most probable parch values were 0.4, 0.3, and 0.1 in the shell, mantle, and core, respectively (Figure 7).

A comparison of the most probable parch values of the K triads underscores their sensitivity to both the number of charged neighbors and the specific zone within the protein. Notably, K triads exhibit higher hydropathy with an increased number of charged residues, ranking as follows: K±± > K ± 0 > K00, regardless of zone. Specifically, within the shell, the most probable parch values for K±±, K ± 0, and K00 are 0.9–1.0, 0.7, and 0.4, respectively. This decreasing trend in most probable parch values also holds true across the shell, mantle, and core zones for any K triad, consistent with the average parch values observed in the database.

The five LL0 triads (LLL, LLA, LLV, LLI, and LLG) exhibit similar parch value distributions, with a most probable parch value of 0.1 in the shell (Figure S22). Notably, LLA shows slightly higher abundance compared to the others, a trend that persists in both the mantle and the core. However, in the mantle, the most probable parch value of the triads drops below 0.1 and eventually reaches 0 in the core.

**Figure 8:**
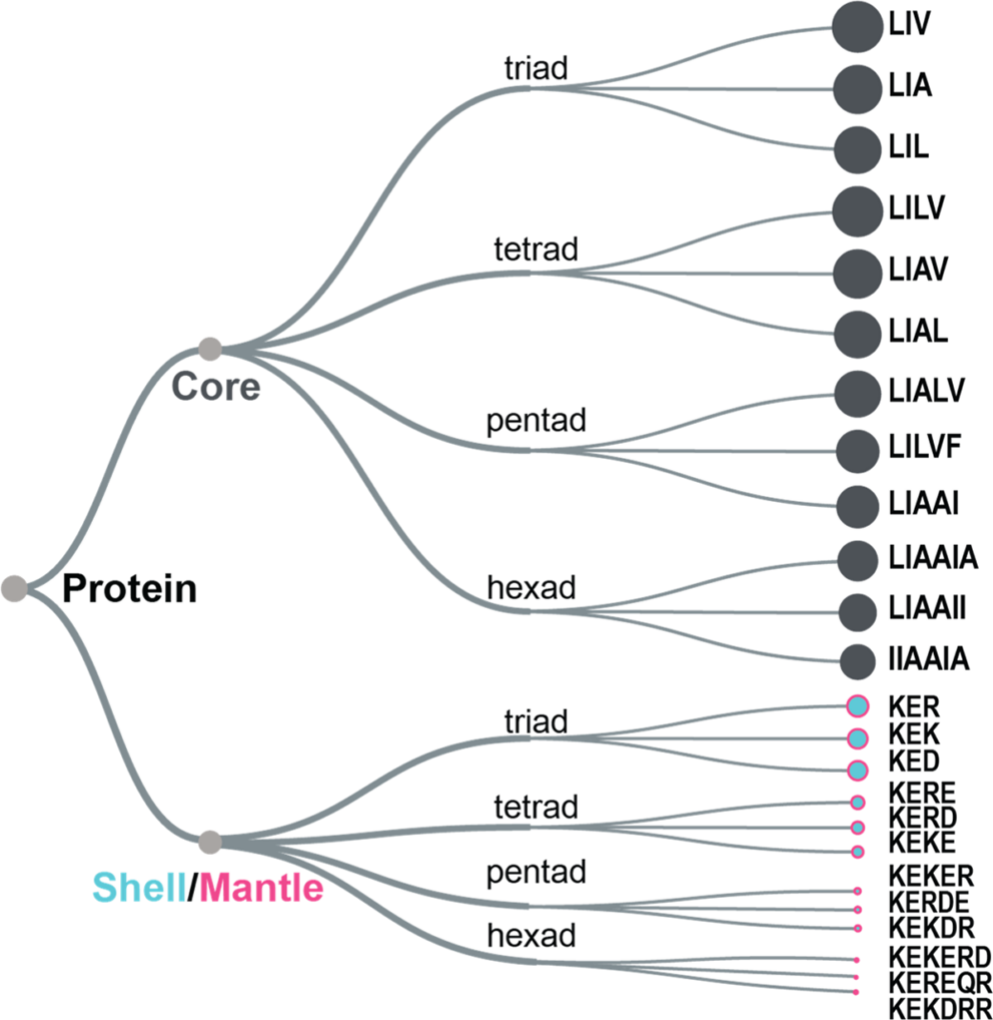
The relative abundance of polyads in the core and shell/mantle regions of the proteins.

Exploring larger neighborhoods such as tetrads, pentads, and beyond revealed distinct patterns between the core and the shell/mantle zones. Various combinations of L, V, I, A, and F residues in the core form tetrads, pentads, and hexads with average parch values nearing 0 (Figure 8). This clustering suggests that these residues interchangeably contribute to achieving similar local hydropathy within the core.

Interestingly, the shell and mantle lack an abundance of tetrads or pentads, indicating significant variability in their local neighborhoods. This variability has several implications: it underscores the rich chemical and topographical heterogeneity of protein surfaces, highlights the role of different residue combinations in tuning local hydropathies and nanoscale architecture for specific biological functions, suggests the absence of concentrated regions of high charge density, and emphasizes the potential consequences of mutations or posttranslational modifications on residues within the shell and mantle zones for the protein’s surface properties.

## Conclusions

In summary, our analysis of 277,877 residues reveals the nuanced interplay between amino acid hydropathy and protein topography. Using the parch scale, we quantify hydropathies, highlighting the impact of neighboring residues. Our study delineates hydropathy profiles across shell, mantle, and core zones, offering a standardized framework for comparative analysis. Additionally, our investigation into residue colocalization underscores the role of local environments in determining protein properties. Overall, our work enhances understanding of protein structure–function relationships, with implications for protein engineering and drug design.

## Data Availability

All structural and computed parch values for the protein data set is available at https://github.com/NangiaLab/ParchValues. There is no restriction on the use of the data.

## Supporting Information Available

Details of the protein data set, protein ranked based on the average parch values of their shells, proteins binned based on their functions, details of the cluster analysis of the protein data set, probability density distributions of parch values in each zone, heat maps of residue dyads and triads, and methods (PDF)
