# Supplementary material for: Nanoscale Topography Dictates Residue Hydropathy in Proteins
Source: Langmuir. 2024 Oct 11;40(42):22049–57. doi: 10.1021/acs.langmuir.4c02142 (PMC11500397; doi:10.1021/acs.langmuir.4c02142)
Supplement: Supplementary file 1 — la4c02142_si_001.pdf [file la4c02142_si_001.pdf]

Nanoscale Topography Dictates Residue Hydropathy in Proteins

Jingjing Ji[1], Advait Dinesh Shukla[2], Ratnakshi Mandal[1], Wafiq Ibsan Khondkar[3], Catilin R Mehl[1], Arindam Chakraborty[4], and Shikha Nangia[1]*

[1]Department of Biomedical and Chemical Engineering, Syracuse University; Syracuse, NY 13244, USA.

[2]Department of Electrical Engineering and Computer Science, Syracuse University; Syracuse, NY 13244, USA.

[3]Department of Biology, Syracuse University; Syracuse, NY 13244, USA.

[4]Department of Chemistry, Syracuse University; Syracuse, NY 13244, USA.

*Corresponding author. Email: snangia@syr.edu

*Address for correspondence:
Dr. Shikha Nangia
343 Link Hall
Department of Biomedical and Chemical Engineering
Syracuse University, Syracuse, NY 13244, USA
Phone (315) 443 0571 | Email: snangia@syr.edu
ORCID 0000-0003-1170-8461

Contents

Table S1. Details of the 1000 protein database. 3
Table S2. Comparison of median parch values residues in the protein database with a subset of hydropathy scales available in the literature....9
Table S3. Proteins rank-ordered (highest to lowest) based on the average parch value (PV) of the shell zone. 10
Figure S1. 1000 proteins binned based on their function. 18
Figure S2. Cluster analysis of 277,877 residues in 1000 proteins. 19
Figure S3. The probability density distributions of the parch values in the three zones. 20
Figure S4. Heat map of residue dyads. 21
Figure S5. Arginine triads 22
Figure S6. Aspartate triads 23
Figure S7. Asparagine triads 24
Figure S8. Glutamine triads 25
Figure S9. Serine triads 26
Figure S10. Threonine triads 27
Figure S11. Tyrosine triads 28
Figure S12. Isoleucine triads 29
Figure S13. Valine triads 30
Figure S14. Alanine triads 31
Figure S15. Glycine triads 32
Figure S16. Histidine triads 33
Figure S17. Proline triads 34
Figure S18. Tryptophan triads 35
Figure S19. Methionine triads 36
Figure S20. Phenylalanine triads 37
Figure S21. Cysteine triads 38
Figure S22. Distribution and parch values of LL0 triads in the shell, mantle, and core regions of proteins. 39
Methods 40
Figure S23. K-means clustering. 41

Table S1. Details of the 1000 protein database.

Protein PDB file arranged in alphanumeric order. The proteins with less than 30% sequence identity and released to the RCSB (https://www.rcsb.org/) repository within the 2018-2023 period were selected.

153l (*25*)	1a3d (*26*)	1aaj (*27*)	1aho (*28*)	1alc (*29*)	1arb (*30*)	1ask (*31*)	1axn (*32*)	1bd8 (*33*)	1c9h (*34*)
1chn (*35*)	1dgk (*36*)	1dhf (*37*)	1eg3 (*38*)	1egf (*39*)	1esa (*40*)	1eyq (*41*)	1ezt (*42*)	1fas (*43*)	1ff4 (*44*)
1fna (*45*)	1fzv (*46*)	1gcn (*47*)	1gy6 (*48*)	1hka (*49*)	1hox (*50*)	1hrs (*51*)	1i6z (*52*)	1ido (*53*)	1ijt (*54*)
1krn (*55*)	1l2p (*56*)	1l6h (*57*)	1lz1 (*58*)	1m60 (*59*)	1mbd (*60*)	1mzl (*61*)	1nep (*62*)	1osa (*63*)	1poe (*64*)
1prh (*65*)	1psn (*66*)	1qxt (*67*)	1rhp (*68*)	1rro (*69*)	1s6d (*70*)	1st3 (*71*)	1su4 (*72*)	1ten (*73*)	1thm (*74*)
1tux (*75*)	1ubq (*76*)	1vhh (*77*)	1w74 (*78*)	1wab (*79*)	1wba (*80*)	1xnb (*81*)	1xnd (*82*)	1z15 (*83*)	2a0b (*84*)
2a3g (*85*)	2a4c (*86*)	2a7u (*87*)	2alg (*88*)	2c55 (*89*)	2che (*90*)	2cpl (*91*)	2dh3 (*92*)	2e4q (*93*)	2e5y (*94*)
2end (*95*)	2hiu (*96*)	2hvm (*97*)	2jwa (*98*)	2l7l (*99*)	2mal (*100*)	2mmg (*101*)	2n1b (*102*)	2oar (*103*)	2ohx (*104*)
2pkc (*105*)	2sfa (*106*)	2st1 (*107*)	2w0g (*108*)	2ypi (*109*)	2z5e (*110*)	3cyt (*111*)	3dwn (*112*)	3ebx (*113*)	3f6l (*114*)
3g2u (*115*)	3gj3 (*116*)	3gpd (*117*)	3hee (*118*)	3onl (*119*)	3pgk (*120*)	3seb (*121*)	3vts (*122*)	4ald (*123*)	4fyp (*124*)
4gq9 (*125*)	4hqa (*126*)	4icb (*127*)	4om5 (*128*)	4pti (*129*)	4pu8 (*130*)	4tpy (*131*)	4ytw (*132*)	5enl (*133*)	5g39 (*134*)
5ggw (*135*)	5mjo (*136*)	5oqk (*137*)	5p21 (*138*)	5rsa (*139*)	5yl6 (*140*)	5yx4 (*141*)	5yxd (*142*)	5z1y (*143*)	5z26 (*144*)
5z2d (*145*)	5z2o (*146*)	5z2w (*147*)	5z31 (*148*)	5z32 (*148*)	5z62 (*149*)	5zcz (*150*)	5zfo (*151*)	5zgg (*152*)	5zrn (*153*)
5zsy (*154*)	5zsz (*155*)	5zuh (*156*)	5zv6 (*157*)	5zvn (*158*)	5zyx (*159*)	6a2b (*160*)	6a3l (*161*)	6a3z (*162*)	6a4c (*163*)
6a5i (*164*)	6a5j (*165*)	6a8y (*166*)	6aab (*167*)	6acl (*168*)	6ahp (*169*)	6ak7 (*170*)	6but (*171*)	6bym (*172*)	6c0a (*173*)
6c37 (*174*)	6c4s (*175*)	6c8u (*176*)	6can (*177*)	6cfa (*178*)	6cgh (*179*)	6cjn (*180*)	6cjz (*181*)	6ckf (*182*)	6cqg (*183*)
6csk (*184*)	6csz (*184*)	6ct1 (*184*)	6ctg (*185*)	6cui (*186*)	6d2h (*187*)	6d6x (*188*)	6d8v (*189*)	6d9p (*190*)	6dk5 (*191*)
6dl4 (*192*)	6drf (*193*)	6dri (*194*)	6drq (*195*)	6ds8 (*196*)	6dst (*197*)	6dul (*198*)	6duu (*199*)	6dz9 (*200*)	6dzb (*200*)

6e1l (*201*)	6e4d (*202*)	6e7e (*203*)	6e98 (*204*)	6e9m (*205*)	6edg (*206*)	6ef8 (*207*)	6f8e (*208*)	6f98 (*209*)	6f9a (*210*)
6fbl (*211*)	6ffq (*212*)	6fgm (*213*)	6fi9 (*214*)	6fmg (*215*)	6fsl (*216*)	6ftk (*217*)	6fzk (*218*)	6g4a (*219*)	6g4k (*220*)
6g7g (*221*)	6g7o (*222*)	6ggz (*223*)	6gig (*224*)	6gms (*225*)	6go0 (*226*)	6go1 (*227*)	6gse (*228*)	6gv9 (*225*)	6gw7 (*229*)
6h0j (*230*)	6h0q (*230*)	6h3e (*231*)	6h7s (*232*)	6h8c (*233*)	6h9f (*234*)	6hkc (*235*)	6hqe (*236*)	6hrc (*237*)	6ht4 (*238*)
6hvk (*239*)	6i2o (*240*)	6i3y (*241*)	6i9b (*242*)	6iar (*243*)	6ib6 (*244*)	6ic4 (*245*)	6if7 (*246*)	6iha (*247*)	6il7 (*248*)
6ilb (*249*)	6im4 (*250*)	6img (*251*)	6imh (*251*)	6ir0 (*252*)	6irr (*253*)	6ixx (*254*)	6iy4 (*255*)	6izg (*256*)	6izv (*257*)
6j07 (*258*)	6j12 (*259*)	6j2e (*260*)	6j2y (*261*)	6j9p (*262*)	6jbr (*263*)	6jcq (*264*)	6jcs (*264*)	6ji7 (*265*)	6jl7 (*266*)
6jn8 (*267*)	6jnp (*268*)	6joy (*269*)	6jp4 (*270*)	6jq8 (*271*)	6jqd (*272*)	6jqe (*272*)	6jsx (*273*)	6jto (*274*)	6jza (*275*)
6k0j (*276*)	6k0o (*277*)	6k2f (*278*)	6k2k (*279*)	6k4v (*280*)	6k4w (*280*)	6k51 (*281*)	6k5t (*282*)	6k7w (*283*)	6k8q (*284*)
6kas (*285*)	6kbv (*286*)	6kcw (*287*)	6kcz (*288*)	6kd0 (*289*)	6kga (*290*)	6kgi (*291*)	6kgj (*291*)	6khu (*292*)	6khv (*283*)
6kjo (*293*)	6klf (*294*)	6klm (*295*)	6kmy (*296*)	6kn1 (*297*)	6kne (*298*)	6kq1 (*299*)	6kqv (*283*)	6kra (*300*)	6krb (*301*)
6ku6 (*302*)	6kwt (*303*)	6kyf (*304*)	6kz4 (*305*)	6l00 (*306*)	6l2h (*307*)	6l4s (*308*)	6l6v (*309*)	6l7n (*310*)	6l7q (*311*)
6l87 (*312*)	6l8r (*313*)	6l8v (*314*)	6l95 (*315*)	6lcy (*316*)	6lf5 (*317*)	6lf8 (*318*)	6lkf (*319*)	6lkl (*320*)	6llq (*321*)
6lr2 (*322*)	6luk (*323*)	6lvh (*324*)	6lwz (*325*)	6lxf (*326*)	6m0y (*327*)	6m3a (*328*)	6m4c (*329*)	6m4z (*330*)	6m55 (*331*)
6m56 (*332*)	6m5c (*333*)	6m6f (*334*)	6m6z (*335*)	6mbm (*336*)	6mgn (*337*)	6mhd (*338*)	6mi9 (*339*)	6mjv (*340*)	6mk1 (*236*)
6mrc (*341*)	6msp (*342*)	6mun (*343*)	6mvm (*344*)	6mw6 (*345*)	6mwm (*346*)	6mzt (*347*)	6n0s (*348*)	6n3a (*349*)	6n9y (*350*)
6nav (*351*)	6nhy (*352*)	6nnb (*353*)	6nnq (*354*)	6nox (*355*)	6npk (*356*)	6nr6 (*357*)	6ntv (*358*)	6nu4 (*359*)	6nvz (*360*)
6nw4 (*361*)	6nw8 (*362*)	6nx4 (*363*)	6ny9 (*364*)	6nzl (*365*)	6nzs (*366*)	6o3q (*367*)	6o3s (*367*)	6o6i (*368*)	6o8t (*369*)
6obi (*370*)	6obk (*371*)	6ocv (*372*)	6odd (*373*)	6ofa (*374*)	6oh1 (*375*)	6oqk (*376*)	6oqp (*377*)	6otb (*378*)	6ovj (*379*)
6p3e (*380*)	6p6b (*381*)	6p73 (*382*)	6pf0 (*383*)	6pmg (*384*)	6poo (*385*)	6por (*386*)	6ppc (*387*)	6pq2 (*388*)	6pqm (*388*)

6prq (*388*)	6pw7 (*389*)	6pw8 (*390*)	6px7 (*391*)	6px8 (*391*)	6q1x (*392*)	6q28 (*393*)	6q2z (*394*)	6q5z (*395*)	6q6e (*396*)
6qax (*397*)	6qb0 (*397*)	6qb1 (*397*)	6qbk (*398*)	6qbl (*398*)	6qbz (*399*)	6qd4 (*400*)	6qes (*401*)	6qet (*401*)	6qeu (*401*)
6qjb (*402*)	6qjh (*403*)	6qk6 (*404*)	6qs0 (*405*)	6qxz (*406*)	6qzt (*407*)	6r0j (*408*)	6r0l (*409*)	6r1b (*410*)	6r1v (*411*)
6r3b (*412*)	6r6o (*413*)	6rf2 (*414*)	6rfd (*414*)	6rfm (*415*)	6rpk (*416*)	6rpr (*417*)	6rqf (*418*)	6rqp (*419*)	6rra (*420*)
6rrl (*421*)	6rro (*421*)	6rsm (*421*)	6ruj (*422*)	6ruz (*423*)	6rwg (*424*)	6ryq (*425*)	6rz1 (*426*)	6rzy (*427*)	6s3v (*428*)
6s3w (*429*)	6s7q (*430*)	6s7v (*431*)	6saa (*432*)	6sab (*432*)	6sai (*433*)	6sap (*434*)	6sjx (*435*)	6so0 (*436*)	6so8 (*437*)
6sof (*438*)	6sxw (*439*)	6sy2 (*440*)	6syg (*441*)	6t3i (*442*)	6taz (*443*)	6tc4 (*444*)	6tdd (*445*)	6tdm (*445*)	6tdn (*445*)
6tey (*446*)	6th8 (*447*)	6tit (*448*)	6tk9 (*449*)	6tkt (*450*)	6tl0 (*451*)	6tl2 (*452*)	6tm6 (*453*)	6tob (*454*)	6tr0 (*455*)
6trp (*456*)	6tub (*457*)	6tuk (*458*)	6tuq (*459*)	6tvm (*460*)	6twg (*461*)	6txt (*462*)	6tya (*463*)	6tyq (*464*)	6u19 (*465*)
6u1o (*466*)	6u1s (*467*)	6u24 (*468*)	6u3g (*469*)	6u3s (*470*)	6u46 (*471*)	6u47 (*472*)	6u6p (*473*)	6u7q (*468*)	6u7r (*468*)
6u7u (*468*)	6u7w (*468*)	6u7x (*468*)	6uch (*474*)	6uhw (*475*)	6urp (*476*)	6urs (*477*)	6ux5 (*478*)	6v4t (*479*)	6v6y (*480*)
6v88 (*481*)	6vba (*482*)	6vfo (*483*)	6vh8 (*484*)	6vhj (*485*)	6vk9 (*486*)	6vla (*487*)	6vrj (*488*)	6vs0 (*489*)	6vti (*490*)
6vtw (*491*)	6vvr (*492*)	6vwb (*493*)	6vxw (*494*)	6vy7 (*495*)	6vyv (*496*)	6w0v (*497*)	6w9n (*498*)	6wa1 (*499*)	6wbo (*500*)
6wbu (*501*)	6wc6 (*502*)	6wes (*503*)	6wi6 (*504*)	6win (*505*)	6wl1 (*506*)	6wpd (*507*)	6wqj (*508*)	6ww4 (*509*)	6wyk (*510*)
6x6n (*511*)	6x7i (*512*)	6x8r (*513*)	6xeh (*514*)	6xmn (*515*)	6xn9 (*516*)	6xor (*517*)	6xp8 (*518*)	6xry (*519*)	6xss (*520*)
6xv2 (*521*)	6xxd (*522*)	6xxe (*522*)	6xyi (*523*)	6xyv (*524*)	6y14 (*525*)	6y4h (*526*)	6y6m (*526*)	6y92 (*527*)	6y96 (*526*)
6ydh (*528*)	6yel (*529*)	6ygn (*530*)	6yhi (*531*)	6yhz (*532*)	6yj0 (*533*)	6ymy (*534*)	6yp7 (*535*)	6yrk (*536*)	6yrr (*537*)
6yse (*538*)	6ysh (*539*)	6ytc (*540*)	6yw8 (*541*)	6z1o (*542*)	6z29 (*543*)	6z41 (*544*)	6z5n (*545*)	6zbi (*546*)	6zfv (*547*)
6zja (*548*)	6zn2 (*549*)	6zss (*244*)	6ztg (*550*)	6zuz (*551*)	6zv1 (*551*)	6zv3 (*551*)	6zv4 (*551*)	6zxp (*552*)	6zyg (*553*)
6zzf (*244*)	7aaf (*554*)	7aao (*555*)	7abm (*556*)	7ac1 (*557*)	7acb (*558*)	7acy (*559*)	7afr (*560*)	7ald (*561*)	7asy (*562*)

7aty (*563*)	7ay8 (*564*)	7azn (*565*)	7b7o (*566*)	7bbb (*567*)	7blo (*568*)	7blq (*568*)	7bpl (*569*)	7bpm (*569*)	7bpn (*569*)
7bqb (*569*)	7bqc (*569*)	7bqd (*569*)	7bqe (*569*)	7bqn (*570*)	7bqq (*571*)	7bqr (*572*)	7bqs (*573*)	7bqv (*574*)	7bra (*575*)
7bv9 (*576*)	7bwo (*577*)	7bx2 (*578*)	7bxy (*579*)	7by7 (*580*)	7c8g (*581*)	7cfv (*582*)	7cia (*583*)	7cir (*584*)	7cix (*585*)
7cjw (*586*)	7ck5 (*587*)	7cke (*588*)	7cm1 (*589*)	7cnb (*590*)	7cnf (*591*)	7cog (*592*)	7cqp (*593*)	7csq (*594*)	7css (*595*)
7cuz (*596*)	7cwh (*597*)	7cy1 (*598*)	7cz6 (*599*)	7czj (*600*)	7d16 (*601*)	7d54 (*602*)	7d5y (*603*)	7d61 (*604*)	7dab (*605*)
7dcv (*606*)	7dee (*607*)	7dfe (*608*)	7di1 (*609*)	7dkr (*610*)	7dla (*611*)	7dm4 (*612*)	7dmd (*613*)	7dme (*613*)	7dmf (*614*)
7dou (*615*)	7drs (*616*)	7dsw (*617*)	7dsx (*617*)	7dz9 (*618*)	7e4j (*619*)	7e4w (*620*)	7ead (*621*)	7eau (*622*)	7edk (*623*)
7eel (*624*)	7eeq (*624*)	7eg5 (*625*)	7egr (*626*)	7elk (*627*)	7ely (*628*)	7ep0 (*629*)	7ep9 (*630*)	7eqm (*631*)	7erv (*632*)
7evq (*633*)	7ewh (*634*)	7f29 (*635*)	7f32 (*636*)	7f7n (*637*)	7f7x (*638*)	7f7y (*639*)	7fbv (*640*)	7fc3 (*641*)	7jgi (*642*)
7jgy (*643*)	7jh1 (*644*)	7jh6 (*645*)	7jia (*646*)	7jiy (*646*)	7jn6 (*647*)	7jnn (*647*)	7jpm (*648*)	7jq8 (*649*)	7js6 (*650*)
7jti (*651*)	7ju9 (*485*)	7jxt (*652*)	7jyn (*649*)	7jyz (*649*)	7k1m (*653*)	7k3g (*654*)	7k3s (*655*)	7k6b (*656*)	7k7a (*657*)
7k7f (*658*)	7k7h (*659*)	7kbq (*660*)	7kcg (*661*)	7kdq (*662*)	7keu (*663*)	7kkw (*664*)	7kl8 (*665*)	7klr (*666*)	7knv (*667*)
7kuw (*668*)	7kwi (*669*)	7l6k (*670*)	7l7a (*671*)	7l83 (*672*)	7l8v (*673*)	7lc8 (*674*)	7lcw (*675*)	7ldf (*676*)	7lgl (*677*)
7lie (*678*)	7lif (*679*)	7lml (*680*)	7lp4 (*681*)	7lp5 (*681*)	7lq5 (*682*)	7lqr (*683*)	7lqs (*683*)	7lqt (*490*)	7lrw (*684*)
7lsp (*685*)	7lt7 (*686*)	7lu8 (*687*)	7luw (*688*)	7lvf (*689*)	7lvg (*689*)	7lwr (*690*)	7lx4 (*691*)	7lxk (*689*)	7lzl (*692*)
7m0g (*693*)	7m1d (*694*)	7m1e (*694*)	7m1w (*695*)	7m59 (*696*)	7m5t (*697*)	7m67 (*698*)	7m73 (*698*)	7mp7 (*699*)	7mu1 (*700*)
7mu9 (*701*)	7my8 (*702*)	7mzz (*703*)	7n02 (*704*)	7n1n (*705*)	7n1o (*706*)	7n23 (*707*)	7n82 (*708*)	7n87 (*709*)	7n99 (*710*)
7ne8 (*711*)	7nee (*712*)	7nqa (*713*)	7ns1 (*714*)	7nwj (*715*)	7nxi (*716*)	7ny0 (*717*)	7o2k (*718*)	7o6w (*719*)	7o77 (*720*)
7o7b (*721*)	7od2 (*722*)	7ofo (*723*)	7oio (*724*)	7oj9 (*725*)	7olg (*726*)	7omk (*727*)	7oo5 (*728*)	7opa (*729*)	7osc (*558*)
7ovc (*730*)	7ovp (*731*)	7ovx (*732*)	7oxf (*733*)	7oz0 (*734*)	7p2k (*735*)	7p2o (*736*)	7p3m (*737*)	7p3o (*738*)	7p3p (*739*)

7p4n (*740*)	7p4w (*741*)	7p4x (*742*)	7p55 (*743*)	7p5g (*743*)	7pbh (*744*)	7pc1 (*745*)	7ph8 (*746*)	7pht (*747*)	7pku (*748*)
7pl4 (*749*)	7pmp (*750*)	7pq4 (*751*)	7pqc (*752*)	7pqw (*753*)	7prd (*754*)	7pvb (*755*)	7pvc (*756*)	7pvm (*757*)	7pzt (*758*)
7q3v (*759*)	7q65 (*760*)	7qab (*761*)	7qap (*762*)	7qb0 (*763*)	7qcc (*764*)	7qdw (*765*)	7qid (*438*)	7qil (*766*)	7qjf (*767*)
7qlf (*768*)	7qv6 (*769*)	7qxj (*770*)	7qzv (*771*)	7r3m (*772*)	7r7m (*773*)	7r7p (*774*)	7rcp (*775*)	7rdr (*776*)	7rfa (*777*)
7rjf (*778*)	7rtm (*779*)	7rul (*780*)	7rx4 (*781*)	7ry6 (*782*)	7rz3 (*783*)	7s3q (*784*)	7s3r (*784*)	7s55 (*785*)	7s5j (*709*)
7s5s (*786*)	7s7k (*787*)	7sag (*788*)	7sci (*789*)	7sek (*790*)	7sf5 (*791*)	7sft (*792*)	7sgt (*793*)	7siy (*794*)	7sjl (*795*)
7skc (*796*)	7skx (*797*)	7sox (*798*)	7swi (*799*)	7sxb (*800*)	7sxi (*801*)	7t2e (*802*)	7t2f (*803*)	7t3h (*804*)	7t6g (*805*)
7t7w (*806*)	7t9x (*807*)	7tb9 (*808*)	7tba (*809*)	7tgp (*810*)	7tjg (*811*)	7tod (*812*)	7tt9 (*813*)	7tvr (*814*)	7txx (*815*)
7tz8 (*816*)	7u0z (*817*)	7u21 (*818*)	7udj (*819*)	7ugc (*820*)	7ui5 (*821*)	7un5 (*822*)	7uur (*823*)	7uv1 (*824*)	7uv3 (*824*)
7uv4 (*824*)	7uwy (*825*)	7uwz (*825*)	7v5e (*826*)	7v5f (*827*)	7v5i (*828*)	7vbg (*829*)	7vd7 (*830*)	7vh9 (*831*)	7vi5 (*832*)
7vij (*833*)	7viv (*834*)	7vma (*835*)	7vq2 (*836*)	7vrg (*837*)	7vsx (*838*)	7vtm (*839*)	7vtr (*840*)	7vu3 (*841*)	7vu5 (*842*)
7vua (*843*)	7vyr (*844*)	7w3w (*845*)	7w5c (*846*)	7w8k (*847*)	7w8r (*847*)	7w8t (*847*)	7w8z (*847*)	7w96 (*847*)	7wag (*848*)
7we3 (*847*)	7wh3 (*849*)	7wnz (*850*)	7wr2 (*851*)	7wr7 (*852*)	7wso (*853*)	7wtj (*854*)	7wwc (*855*)	7wyr (*856*)	7wz5 (*857*)
7x59 (*858*)	7x5c (*859*)	7x7m (*860*)	7x7w (*861*)	7x89 (*862*)	7x9u (*863*)	7xb6 (*864*)	7xez (*865*)	7xfg (*866*)	7xfs (*867*)
7xga (*868*)	7xhz (*869*)	7xrw (*870*)	7y13 (*871*)	7y3j (*872*)	7yat (*873*)	7yf1 (*874*)	7ywq (*875*)	7ywr (*876*)	7yyi (*877*)
7z3c (*878*)	7zbs (*879*)	7zcl (*880*)	7zey (*881*)	7zez (*881*)	7zgb (*882*)	7zok (*883*)	7zqe (*884*)	7zro (*885*)	7zru (*886*)
7zsj (*887*)	7zwj (*888*)	7zzv (*889*)	8a04 (*890*)	8a06 (*890*)	8a7z (*891*)	8a8n (*892*)	8ab1 (*893*)	8aig (*891*)	8ajr (*894*)
8all (*891*)	8ao1 (*895*)	8ar0 (*896*)	8ar1 (*896*)	8ar3 (*896*)	8atd (*897*)	8b0u (*898*)	8b1l (*899*)	8bfk (*900*)	8boo (*901*)
8bvl (*902*)	8c18 (*903*)	8c1c (*904*)	8c7k (*905*)	8cio (*906*)	8czc (*907*)	8ddj (*908*)	8dft (*909*)	8dfu (*909*)	8dh7 (*910*)
8dmk (*911*)	8doa (*803*)	8dpx (*912*)	8dtu (*913*)	8dym (*914*)	8ef4 (*915*)	8ehs (*916*)	8eht (*916*)	8ert (*917*)	8eu7 (*918*)

8f2f (*919*)	8f41 (*920*)	8f68 (*921*)	8fey (*922*)	8fg2 (*923*)	8flp (*924*)	8gq0 (*925*)	8gs7 (*926*)	8gvn (*927*)	8gyx (*928*)
8h0u (*929*)	8h6r (*930*)	8hfo (*931*)	8hgr (*932*)	8hjd (*933*)	8hz1 (*934*)	8hz2 (*935*)	8ib0 (*936*)	8il3 (*937*)	8rnt (*938*)

Table S2. Comparison of median parch values residues in the protein database with a subset of hydropathy scales available in the literature.

The hydropathy values are formatted in color to demonstrate the hydrophobic (blue) to hydrophilic (red) residues.

Residue	Kyte-Doolittle[a]	Eisenberg[b]	Rose[c]	Janin[d]	Engelman GES[e]	PARCH
A	1.80	0.62	0.74	0.30	1.60	0.04
C	2.50	0.29	0.91	0.90	2.00	0.04
D	−3.50	−0.90	−0.60	−0.60	−9.20	0.65
E	−3.50	−0.74	−0.70	−0.70	−8.20	0.89
F	2.80	1.19	0.50	0.50	3.70	0.09
G	−0.40	0.48	0.30	0.30	1.00	0.06
H	3.20	−0.40	−0.10	−0.10	−3.00	0.32
I	4.50	1.38	0.70	0.70	3.10	0.05
K	−3.90	−1.50	−1.80	−1.80	−8.80	0.97
L	3.80	1.06	0.50	0.50	2.80	0.06
M	1.90	0.64	0.40	0.40	3.40	0.09
N	−3.50	−0.78	−0.50	−0.50	−4.80	0.25
P	−1.60	0.12	−0.30	−0.30	−0.20	0.15
Q	−3.50	−0.85	−0.70	−0.70	−4.10	0.28
R	−4.50	−2.53	−1.40	−1.40	−12.30	0.98
S	−0.80	−0.18	−0.10	−0.10	0.60	0.14
T	−0.70	−0.05	−0.20	−0.20	1.20	0.15
V	4.20	1.08	0.60	0.60	2.60	0.05
W	−0.90	0.81	0.30	0.30	1.90	0.21
Y	−1.30	0.76	−0.40	−0.40	−0.70	0.22

[a]**Kyte-Doolittle:** Scale range −4.5 to +4.5. Lower values are hydrophilic, higher values are hydrophobic.
Kyte, J. and Doolittle, R. F. (1982). A simple method for displaying the hydropathic character of a protein. J Mol Biol, 157(1):105-132.

[b]**Eisenberg:** Normalized hydropathy scale. Values range −3 to +2. Lower values are hydrophilic, higher values are hydrophobic.
Eisenberg, D., Schwarz, E., Komaromy, M., and Wall, R. (1984). Analysis of membrane and surface protein sequences with the hydrophobic moment plot. J Mol Biol, 179(1):125-142.

[c]**Rose**: Lower values are hydrophilic, higher values are hydrophobic.
Rose, G. D., Geselowitz, A. R., Lesser, G. J., Lee, R. H., and Zehfus, M. H. (1985). Hydrophobicity of amino acid residues in globular proteins. Science, 229(4716):834-838.

[d]**Janin:** Lower values are hydrophilic, higher values are hydrophobic.
Janin, J. (1979). Surface and inside volumes in globular proteins. Nature, 277(5696):491-492.

[e]**Engelman GES:** Lower values are hydrophilic, higher values are hydrophobic.
Engelman, D. M., Steitz, T. A., and Goldman, A. (1986). Identifying nonpolar transbilayer helices in amino acid sequences of membrane proteins. Annu Rev Biophys Biophys Chem, 15:321-353.

Table S3. Proteins rank-ordered (highest to lowest) based on the average parch value (PV) of the shell zone.

Pdb ID	Rank	PV (avg)
6r0l	1	1.6072
6x8r	2	1.5200
6mk1	3	1.5002
8ert	4	1.4882
6mrc	5	1.4259
6but	6	1.3842
7lt7	7	1.3279
8f41	8	1.3235
7yat	9	1.3142
6fi9	10	1.3090
3g2u	11	1.3082
7keu	12	1.3073
7cqp	13	1.2937
6wl1	14	1.2871
6mvm	15	1.2810
6can	16	1.2797
7bx2	17	1.2792
6dzb	18	1.2752
7kuw	19	1.2703
7lrw	20	1.2685
6urp	21	1.2660
6drq	22	1.2644
7pl4	23	1.2627
7zey	24	1.2616
7lwr	25	1.2561
7lvg	26	1.2544
6pmg	27	1.2533
5zrn	28	1.2512
8dym	29	1.2458
6v6y	30	1.2444
7sft	31	1.2433
6pw7	32	1.2421
7cnf	33	1.2414
6u1s	34	1.2347
6rsm	35	1.2313
6p3e	36	1.2313
7olg	37	1.2306
6hqe	38	1.2304
8b1l	39	1.2296
6kne	40	1.2284
6krb	41	1.2231
6hvk	43	1.2209
7r7m	44	1.2157
6o6i	45	1.2122
7drs	46	1.2112
7ep9	47	1.2102
6l4s	48	1.2091
7cwh	49	1.2083
7x59	50	1.2081
6s7q	51	1.1974
2e5y	52	1.1947
6l7q	53	1.1912
7lgl	54	1.1910
1vhh	55	1.1885
7rx4	56	1.1883
6cgh	57	1.1877
6wbo	58	1.1874
7t7w	59	1.1859
7vd7	60	1.1851
7zez	61	1.1848
6m0y	62	1.1790
6nw4	63	1.1785
3pgk	64	1.1733
7uv3	65	1.1730
7bv9	66	1.1714
6r1v	67	1.1670
7abm	68	1.1665
8atd	69	1.1629
7m0g	70	1.1626
6bym	71	1.1570
1dgk	72	1.1557
7jgi	73	1.1550
7vma	74	1.1550
6r0j	75	1.1544
7dz9	76	1.1522
6jsx	77	1.1510
7bbb	78	1.1509
6mjv	79	1.1508
7ewh	80	1.1487
7v5i	81	1.1486
6u7u	82	1.1457
6vrj	83	1.1456
6h9f	85	1.1416
6ysh	86	1.1414
6rf2	87	1.1410
7bpn	88	1.1407
7lcw	89	1.1397
7n1n	90	1.1396
7pku	91	1.1387
7e4j	92	1.1355
7kbq	93	1.1339
2mmg	94	1.1333
2ypi	95	1.1323
2l7l	96	1.1321
6u7x	97	1.1306
6rra	98	1.1290
7qzv	99	1.1274
6wqj	100	1.1259
6lkf	101	1.1258
7bxy	102	1.1254
4hqa	103	1.1236
7lvf	104	1.1228
8dmk	105	1.1225
7opa	106	1.1224
6rpr	107	1.1214
7udj	108	1.1213
6k2f	109	1.1213
6jto	110	1.1209
6zv4	111	1.1196
7jh1	112	1.1167
6ruj	113	1.1160
7wnz	114	1.1142
7l8v	115	1.1121
7bqr	116	1.1119
7vi5	117	1.1117
6lkl	118	1.1115
6zja	119	1.1115
6n0s	120	1.1107
7tod	121	1.1103
6vti	122	1.1082
7dsx	123	1.1069
7dmd	124	1.1061
1chn	125	1.1040

7swi	42	1.2211
**Pdb ID**	**Rank**	**PV (avg)**
6kgi	127	1.1021
7uwy	128	1.1020
7evq	129	1.1019
6ygn	130	1.1015
7zgb	131	1.1006
6jnp	132	1.0998
6p73	133	1.0996
1s6d	134	1.0993
7egr	135	1.0975
4icb	136	1.0974
7o6w	137	1.0972
7c8g	138	1.0971
6mhd	139	1.0968
3f6l	140	1.0956
6ilb	141	1.0951
6vfo	142	1.0949
1ido	143	1.0944
2cpl	144	1.0938
6nnq	145	1.0935
7blq	146	1.0917
1hox	147	1.0905
7pvm	148	1.0896
8hz1	149	1.0894
7jn6	150	1.0894
6jqd	151	1.0890
3hee	152	1.0880
6xss	153	1.0875
6ak7	154	1.0870
5z62	155	1.0831
7sci	156	1.0817
7sek	157	1.0800
7ywr	158	1.0790
6wbu	159	1.0776
7qid	160	1.0756
7uv1	161	1.0753
6ruz	162	1.0743
7jyn	163	1.0739
7qlf	164	1.0733
6nr6	165	1.0727
6qzt	166	1.0726
8dpx	167	1.0718
7js6	168	1.0717

6zbi	84	1.1449
**Pdb ID**	**Rank**	**PV (avg)**
1dhf	169	1.0693
7lif	170	1.0685
6ib6	171	1.0683
7jxt	172	1.0677
6kas	173	1.0676
6ic4	174	1.0674
7kl8	175	1.0638
6a3l	176	1.0635
7cia	177	1.0634
6oqk	178	1.0627
6o3q	179	1.0624
8b0u	180	1.0622
6sai	181	1.0620
7bra	182	1.0582
6edg	183	1.0580
1qxt	184	1.0575
5z26	185	1.0574
7y3j	186	1.0571
6n9y	187	1.0569
7uur	188	1.0568
6j2e	189	1.0563
7pqc	190	1.0562
7zwj	191	1.0562
1alc	192	1.0558
7t2f	193	1.0556
7xb6	194	1.0549
8ajr	195	1.0548
6fsl	196	1.0541
6k5t	197	1.0540
7jq8	198	1.0538
6dri	199	1.0527
7wr2	200	1.0509
6khv	201	1.0507
4ytw	202	1.0493
6rfd	203	1.0473
6xp8	204	1.0462
6mgn	205	1.0457
7ovc	206	1.0452
6tub	207	1.0451
8h6r	208	1.0450
8dtu	209	1.0447
6kjo	210	1.0437

8a8n	126	1.1040
**Pdb ID**	**Rank**	**PV (avg)**
6lvh	211	1.0413
6ku6	212	1.0399
7rdr	213	1.0398
6u24	214	1.0379
7tba	215	1.0377
7e4w	216	1.0375
7u21	217	1.0374
7acb	218	1.0367
5zyx	219	1.0352
7cir	220	1.0350
6px8	221	1.0347
6jza	222	1.0328
6r6o	223	1.0320
7o77	224	1.0315
6yel	225	1.0314
6tk9	226	1.0307
7vtr	227	1.0302
6izg	228	1.0300
6rqf	229	1.0287
6lf8	230	1.0277
4fyp	231	1.0277
6twg	232	1.0258
1prh	233	1.0255
3gpd	234	1.0251
6sof	235	1.0238
6ryq	236	1.0236
6o8t	237	1.0218
8dh7	238	1.0202
5p21	239	1.0198
7d16	240	1.0198
1fzv	241	1.0193
5enl	242	1.0189
6m4c	243	1.0186
7ep0	244	1.0184
7rtm	245	1.0182
6u19	246	1.0171
6yw8	247	1.0166
7nee	248	1.0161
6g7o	249	1.0160
6q2z	250	1.0159
5z31	251	1.0157
7n82	252	1.0149

Pdb ID	Rank	PV (avg)
6iy4	253	1.0143
8a04	254	1.0142
6h3e	255	1.0138
6h7s	256	1.0134
6qeu	257	1.0133
6zyg	258	1.0131
6ymy	259	1.0129
6y6m	260	1.0128
7ne8	261	1.0121
6vwb	262	1.0115
6kn1	263	1.0111
6sjx	264	1.0101
8ddj	265	1.0095
1su4	266	1.0081
7uwz	267	1.0078
6a2b	268	1.0071
7tt9	269	1.0065
7dou	270	1.0063
6yrr	271	1.0062
6vyv	272	1.0057
7blo	273	1.0054
8aig	274	1.0041
7vij	275	1.0032
1rro	276	1.0015
2a4c	277	1.0013
7lml	278	1.0001
7r7p	279	0.9997
7vrg	280	0.9988
7d5y	281	0.9977
6tdm	282	0.9976
7zcl	283	0.9966
7wwc	284	0.9965
8c1c	285	0.9957
6tvm	286	0.9953
6l87	287	0.9951
6ny9	288	0.9950
7ui5	289	0.9947
5z2o	290	0.9945
6r3b	291	0.9938
7bqq	292	0.9937
5zuh	293	0.9936
6ht4	294	0.9933

Pdb ID	Rank	PV (avg)
1m60	295	0.9925
7l6k	296	0.9920
7jnn	297	0.9904
6kwt	298	0.9899
7mp7	299	0.9897
6qs0	300	0.9896
1eyq	301	0.9894
6z29	302	0.9885
7eg5	303	0.9884
7csq	304	0.9874
7dm4	305	0.9870
7oxf	306	0.9867
6m6z	307	0.9863
7f32	308	0.9862
7dee	309	0.9860
7siy	310	0.9851
6z1o	311	0.9847
6so8	312	0.9845
7zzv	313	0.9845
7nqa	314	0.9833
6msp	315	0.9828
8h0u	316	0.9821
7vbg	317	0.9820
7n02	318	0.9815
8dfu	319	0.9805
7pqw	320	0.9800
6jl7	321	0.9792
8fg2	322	0.9788
5yxd	323	0.9787
6a3z	324	0.9786
7lqt	325	0.9770
7sf5	326	0.9768
7xrw	327	0.9767
8f2f	328	0.9764
7mu1	329	0.9761
6xor	330	0.9759
6kd0	331	0.9749
2ohx	332	0.9746
7k3g	333	0.9745
7t2e	334	0.9743
7m73	335	0.9738
2w0g	336	0.9734

Pdb ID	Rank	PV (avg)
6taz	337	0.9730
1mbd	338	0.9728
7bqe	339	0.9718
7ldf	340	0.9715
6tob	341	0.9705
7q65	342	0.9701
6y4h	343	0.9699
5z32	344	0.9689
7n87	345	0.9688
7eeq	346	0.9686
7acy	347	0.9684
7ry6	348	0.9684
6i3y	349	0.9682
2a0b	350	0.9680
6dl4	351	0.9679
8ehs	352	0.9674
1z15	353	0.9673
6cqg	354	0.9668
7ovp	355	0.9663
6o3s	356	0.9658
6y14	357	0.9645
6g4a	358	0.9644
6jbr	359	0.9644
7uv4	360	0.9642
7p4n	361	0.9636
6qd4	362	0.9633
6d2h	363	0.9629
1axn	364	0.9622
7bpm	365	0.9617
7w8t	366	0.9613
7rjf	367	0.9612
1wab	368	0.9609
6d8v	369	0.9604
6xeh	370	0.9603
6w0v	371	0.9598
6kra	372	0.9576
5yl6	373	0.9572
7erv	374	0.9568
6zv3	375	0.9548
6qet	376	0.9547
5ggw	377	0.9546
7vq2	378	0.9546

Pdb ID	Rank	PV (avg)
7sox	379	0.9545
3cyt	380	0.9520
6go1	381	0.9502
7bqb	382	0.9497
6y92	383	0.9494
8eht	384	0.9490
6vh8	385	0.9488
6zxp	386	0.9487
7vua	387	0.9482
6kga	388	0.9467
7m67	389	0.9459
6q5z	390	0.9442
1hka	391	0.9439
5zvn	392	0.9436
7cjw	393	0.9418
7x5c	394	0.9417
7qcc	395	0.9416
1ubq	396	0.9415
7ywq	397	0.9414
6odd	398	0.9407
6jp4	399	0.9405
7bqd	400	0.9403
7zqe	401	0.9402
7jh6	402	0.9401
7s5j	403	0.9397
7tjg	404	0.9395
6mun	405	0.9393
5mjo	406	0.9382
1aho	407	0.9376
4ald	408	0.9361
6hrc	409	0.9361
7r3m	410	0.9361
6go0	411	0.9360
7x7m	412	0.9353
8czc	413	0.9344
6ctg	414	0.9342
6yhz	415	0.9332
6xmn	416	0.9330
6r1b	417	0.9325
6obi	418	0.9302
6c0a	419	0.9294
7dme	420	0.9294

Pdb ID	Rank	PV (avg)
7xga	421	0.9279
6v88	422	0.9270
7afr	423	0.9267
6hkc	424	0.9265
6kyf	425	0.9263
6zn2	426	0.9263
6qxz	427	0.9259
7klr	428	0.9256
7bqs	429	0.9256
7xhz	430	0.9251
7by7	431	0.9249
2z5e	432	0.9248
6jqe	433	0.9246
6g7g	434	0.9245
6pqm	435	0.9243
6u6p	436	0.9237
7wr7	437	0.9233
6im4	438	0.9231
5zsz	439	0.9231
6a5i	440	0.9230
6l6v	441	0.9224
7f7n	442	0.9223
6k0j	443	0.9220
8dft	444	0.9220
6lxf	445	0.9216
6j9p	446	0.9214
6ntv	447	0.9211
6cui	448	0.9201
7w5c	449	0.9199
6jq8	450	0.9198
7edk	451	0.9197
3gj3	452	0.9195
7k3s	453	0.9195
6px7	454	0.9194
6j07	455	0.9192
6rzy	456	0.9191
6th8	457	0.9191
7zok	458	0.9187
2dh3	459	0.9185
6h8c	460	0.9169
7k7f	461	0.9164
6qbl	462	0.9158

Pdb ID	Rank	PV (avg)
7p3o	463	0.9155
6wc6	464	0.9153
6d9p	465	0.9148
8gvn	466	0.9142
7rul	467	0.9141
4gq9	468	0.9135
6lr2	469	0.9132
7skc	470	0.9126
7jyz	471	0.9125
7eel	472	0.9121
6kq1	473	0.9099
6kqv	474	0.9099
7f7x	475	0.9094
6izv	476	0.9091
7ovx	477	0.9090
6fmg	478	0.9084
1bd8	479	0.9082
6i9b	480	0.9073
6gse	481	0.9060
7s3q	482	0.9059
6s3v	483	0.9059
7w3w	484	0.9050
6u47	485	0.9040
7xfs	486	0.9039
6sy2	487	0.9038
6a4c	488	0.9017
6acl	489	0.9017
7cuz	490	0.9015
7n99	491	0.9008
6wa1	492	0.9007
7t6g	493	0.9004
7pc1	494	0.9000
6k8q	495	0.8994
6uhw	496	0.8990
5zfo	497	0.8987
7bqc	498	0.8978
1w74	499	0.8969
6m55	500	0.8968
1wba	501	0.8968
7cix	502	0.8968
3onl	503	0.8955
8il3	504	0.8954

Pdb ID	Rank	PV (avg)
6nnb	505	0.8952
7bqn	506	0.8950
7sxi	507	0.8940
6rwg	508	0.8934
6l8r	509	0.8922
8all	510	0.8903
8a06	511	0.8898
6syg	512	0.8893
7oj9	513	0.8885
6e4d	514	0.8882
6ir0	515	0.8878
6qbk	516	0.8875
6ixx	517	0.8872
6tuk	518	0.8871
1ijt	519	0.8862
7ny0	520	0.8862
6joy	521	0.8853
6tdn	522	0.8849
6f9a	523	0.8847
6ahp	524	0.8846
6kcz	525	0.8825
7jti	526	0.8822
8ab1	527	0.8821
6e9m	528	0.8819
7fbv	529	0.8819
7un5	530	0.8817
7zsj	531	0.8813
2che	532	0.8812
7lp5	533	0.8810
7f29	534	0.8806
7mu9	535	0.8797
6lcy	536	0.8794
1rhp	537	0.8786
8f68	538	0.8782
2a3g	539	0.8780
7qil	540	0.8778
6ckf	541	0.8775
7o7b	542	0.8769
6qbz	543	0.8769
5z2w	544	0.8760
5z1y	545	0.8741
6ztg	546	0.8735

Pdb ID	Rank	PV (avg)
7vyr	547	0.8731
7vu3	548	0.8728
7x7w	549	0.8725
7dmf	550	0.8713
7yf1	551	0.8711
6x6n	552	0.8703
7pzt	553	0.8702
7nxi	554	0.8686
3seb	555	0.8683
6vba	556	0.8676
6pq2	557	0.8673
7k7a	558	0.8672
6u7r	559	0.8667
7ugc	560	0.8666
7yyi	561	0.8664
7asy	562	0.8662
7dcv	563	0.8634
7tz8	564	0.8628
6u3g	565	0.8625
1a3d	566	0.8625
7n1o	567	0.8612
1ezt	568	0.8611
8gyx	569	0.8606
6iha	570	0.8605
6il7	571	0.8603
7k6b	572	0.8601
8flp	573	0.8593
7azn	574	0.8590
2end	575	0.8584
1l2p	576	0.8583
7t3h	577	0.8581
7pbh	578	0.8579
2oar	579	0.8575
5g39	580	0.8569
7cfv	581	0.8562
6vvr	582	0.8557
6kz4	583	0.8553
5zgg	584	0.8540
6nzs	585	0.8530
7cy1	586	0.8521
7k7h	587	0.8510
8hfo	588	0.8505

Pdb ID	Rank	PV (avg)
6klf	589	0.8501
6csk	590	0.8500
7sxb	591	0.8488
6fzk	592	0.8485
7bqv	593	0.8483
6zuz	594	0.8477
6k7w	595	0.8475
6c4s	596	0.8475
8boo	597	0.8469
6vxw	598	0.8459
7knv	599	0.8455
6khu	600	0.8454
7s5s	601	0.8453
7cm1	602	0.8441
6j12	603	0.8433
6tc4	604	0.8432
7w8z	605	0.8429
6jcs	606	0.8414
6npk	607	0.8411
7bpl	608	0.8407
7pvc	609	0.8404
6sap	610	0.8403
6irr	611	0.8402
1aaj	612	0.8393
7oz0	613	0.8393
1osa	614	0.8379
6vla	615	0.8377
6u3s	616	0.8371
6f8e	617	0.8371
7lxk	618	0.8356
6u7q	619	0.8352
6qjh	620	0.8342
7wtj	621	0.8341
8ib0	622	0.8340
6s3w	623	0.8336
7fc3	624	0.8327
6j2y	625	0.8326
7sjl	626	0.8314
7jia	627	0.8313
7qdw	628	0.8296
2n1b	629	0.8294
153l	630	0.8292

Pdb ID	Rank	PV (avg)	Pdb ID	Rank	PV (avg)	Pdb ID	Rank	PV (avg)
6u1o	631	0.8292	6k4w	673	0.8075	7ac1	715	0.7875
6jcq	632	0.8283	7zro	674	0.8073	7vsx	716	0.7871
1i6z	633	0.8263	1ask	675	0.8071	6f98	717	0.7870
6llq	634	0.8261	6jn8	676	0.8065	1c9h	718	0.7863
7aaf	635	0.8258	6x7i	677	0.8060	7viv	719	0.7858
6p6b	636	0.8257	6kgj	678	0.8056	1krn	720	0.7856
6mi9	637	0.8257	7cke	679	0.8055	6y96	721	0.7841
6rqp	638	0.8253	7xez	680	0.8052	6tyq	722	0.7839
7kkw	639	0.8249	7pht	681	0.8048	7w8r	723	0.7838
7x89	640	0.8243	7kdq	682	0.8045	6ww4	724	0.7836
6uch	641	0.8241	7s3r	683	0.8043	1hrs	725	0.7835
5zcz	642	0.8238	7lzl	684	0.8038	6yj0	726	0.7835
7qv6	643	0.8236	6ct1	685	0.8037	6txt	727	0.7822
6rpk	644	0.8231	6ovj	686	0.8022	7dab	728	0.7821
6qes	645	0.8227	6yp7	687	0.8018	4pti	729	0.7819
7d54	646	0.8227	6xyi	688	0.7991	7w96	730	0.7812
7pq4	647	0.8210	6u46	689	0.7976	7dsw	731	0.7804
6l8v	648	0.8208	2e4q	690	0.7976	2a7u	732	0.7803
6qk6	649	0.8201	6m4z	691	0.7970	6k0o	733	0.7802
7tb9	650	0.8200	7zbs	692	0.7967	6mbm	734	0.7800
6oh1	651	0.8196	1eg3	693	0.7967	7lqr	735	0.7792
7u0z	652	0.8195	7wz5	694	0.7965	6l7n	736	0.7786
6kbv	653	0.8190	7jpm	695	0.7958	6c8u	737	0.7780
6dk5	654	0.8187	6iar	696	0.7954	7eqm	738	0.7766
8ar1	655	0.8185	6trp	697	0.7953	6m56	739	0.7765
7wso	656	0.8179	7bwo	698	0.7948	7dkr	740	0.7765
4pu8	657	0.8168	6poo	699	0.7941	1poe	741	0.7763
5z2d	658	0.8165	7m5t	700	0.7941	6dst	742	0.7759
8fey	659	0.8160	1egf	701	0.7938	1lz1	743	0.7757
6vtw	660	0.8159	6wyk	702	0.7938	6sxw	744	0.7744
7ju9	661	0.8148	6w9n	703	0.7934	7p4w	745	0.7740
7dfe	662	0.8142	6nx4	704	0.7933	7kwi	746	0.7740
6c37	663	0.8141	7lu8	705	0.7930	7ofo	747	0.7736
6m6f	664	0.8140	8a7z	706	0.7927	6a8y	748	0.7725
6cjn	665	0.8139	7di1	707	0.7925	7vtm	749	0.7725
6tit	666	0.8138	7aao	708	0.7921	6qb0	750	0.7719
6a5j	667	0.8137	6xn9	709	0.7917	6e7e	751	0.7682
7f7y	668	0.8136	6xv2	710	0.7911	7lie	752	0.7682
6d6x	669	0.8126	7eau	711	0.7909	7p2k	753	0.7662
6q28	670	0.8115	6q6e	712	0.7903	5oqk	754	0.7657
6qjb	671	0.8107	7wyr	713	0.7889	7n23	755	0.7657
8ao1	672	0.8080	7oio	714	0.7883	7aty	756	0.7655

Pdb ID	Rank	PV (avg)
6z5n	757	0.7643
1nep	758	0.7636
8hz2	759	0.7636
6tdd	760	0.7634
7nwj	761	0.7632
8bvl	762	0.7618
2hvm	763	0.7617
7wh3	764	0.7617
6nw8	765	0.7610
6l2h	766	0.7607
6kcw	767	0.7602
6tkt	768	0.7594
6xry	769	0.7590
6luk	770	0.7583
6zzf	771	0.7566
7m1d	772	0.7565
8doa	773	0.7564
7xfg	774	0.7555
6sab	775	0.7548
7b7o	776	0.7544
6nu4	777	0.7524
7we3	778	0.7522
6tya	779	0.7512
6v4t	780	0.7505
6xyv	781	0.7502
6t3i	782	0.7497
6ocv	783	0.7490
6saa	784	0.7485
7cnb	785	0.7479
7kcg	786	0.7467
6l00	787	0.7454
6m3a	788	0.7440
6nzl	789	0.7438
8c7k	790	0.7438
6yrk	791	0.7433
6ds8	792	0.7421
6zv1	793	0.7418
2st1	794	0.7417
7p55	795	0.7415
6tl0	796	0.7403
6yse	797	0.7400
6nvz	798	0.7391

Pdb ID	Rank	PV (avg)
6s7v	799	0.7381
7s7k	800	0.7368
7tgp	801	0.7367
8c18	802	0.7359
7qap	803	0.7351
6wes	804	0.7335
7omk	805	0.7321
6zfv	806	0.7319
5rsa	807	0.7317
1tux	808	0.7311
2c55	809	0.7307
7mzz	810	0.7303
1l6h	811	0.7292
7x9u	812	0.7275
7sag	813	0.7264
6vy7	814	0.7263
7skx	815	0.7255
6ydh	816	0.7251
7qb0	817	0.7243
6drf	818	0.7235
7qxj	819	0.7219
8rnt	820	0.7213
3vts	821	0.7211
2hiu	822	0.7202
7m1w	823	0.7170
6tl2	824	0.7143
3dwn	825	0.7136
6tm6	826	0.7122
6urs	827	0.7104
6z41	828	0.7092
8ef4	829	0.7059
6fbl	830	0.7056
6h0j	831	0.7040
6csz	832	0.7036
7m59	833	0.7036
6xxd	834	0.7036
7lx4	835	0.7021
6gms	836	0.7016
6qb1	837	0.7008
7txx	838	0.7007
6win	839	0.7000
7ck5	840	0.6998

Pdb ID	Rank	PV (avg)
7qjf	841	0.6983
7wag	842	0.6974
5yx4	843	0.6965
1gy6	844	0.6963
7z3c	845	0.6937
2pkc	846	0.6924
6gw7	847	0.6910
6prq	848	0.6896
6duu	849	0.6894
6obk	850	0.6886
6tey	851	0.6885
1esa	852	0.6877
8eu7	853	0.6862
2jwa	854	0.6857
6if7	855	0.6846
6wpd	856	0.6841
6i2o	857	0.6836
7qab	858	0.6829
7jiy	859	0.6826
1ff4	860	0.6817
6dz9	861	0.6812
6gig	862	0.6807
7sgt	863	0.6807
6ppc	864	0.6798
7w8k	865	0.6791
7oo5	866	0.6786
6rrl	867	0.6767
7pmp	868	0.6754
7lsp	869	0.6750
8bfk	870	0.6749
6ef8	871	0.6745
7ph8	872	0.6744
7jgy	873	0.6744
6oqp	874	0.6739
1fna	875	0.6721
7ald	876	0.6689
6rz1	877	0.6684
6mw6	878	0.6676
6zss	879	0.6670
7v5f	880	0.6664
7rz3	881	0.6663
1gcn	882	0.6662

Pdb ID	Rank	PV (avg)
6ji7	883	0.6636
6mzt	884	0.6631
7zru	885	0.6622
6ftk	886	0.6613
7vh9	887	0.6610
8hgr	888	0.6602
6so0	889	0.6590
6klm	890	0.6575
6cjz	891	0.6565
6pf0	892	0.6552
6e1l	893	0.6550
7d61	894	0.6532
6qax	895	0.6515
6yhi	896	0.6483
6k2k	897	0.6475
6lf5	898	0.6439
6mwm	899	0.6424
8gs7	900	0.6420
6pw8	901	0.6419
7od2	902	0.6414
7m1e	903	0.6377
7rcp	904	0.6353
6wi6	905	0.6349
7elk	906	0.6340
6vs0	907	0.6322
7p3p	908	0.6315
6ggz	909	0.6304
7ely	910	0.6303
1fas	911	0.6284
6kmy	912	0.6276
6aab	913	0.6267
8gq0	914	0.6250
7lq5	915	0.6239
6h0q	916	0.6237
5zsy	917	0.6235
6k4v	918	0.6232
7lc8	919	0.6213
7vu5	920	0.6210
6cfa	921	0.6203
6fgm	922	0.6190
7luw	923	0.6174
6k51	924	0.6158

Pdb ID	Rank	PV (avg)
2mal	925	0.6149
6dul	926	0.6133
6img	927	0.6100
1psn	928	0.6100
6ytc	929	0.6065
1xnb	930	0.6046
6ux5	931	0.6041
7lqs	932	0.6039
6rro	933	0.6024
7l83	934	0.6014
7tvr	935	0.5993
1ten	936	0.5968
6tr0	937	0.5946
6xxe	938	0.5914
7p2o	939	0.5887
7dla	940	0.5868
6e98	941	0.5851
7v5e	942	0.5844
7pvb	943	0.5844
6n3a	944	0.5840
6ffq	945	0.5809
7p5g	946	0.5807
6ofa	947	0.5787
7lp4	948	0.5785
6nhy	949	0.5717
7ead	950	0.5714
7t9x	951	0.5705
1mzl	952	0.5594
7prd	953	0.5574
3ebx	954	0.5573
7s55	955	0.5548
7q3v	956	0.5516
6rfm	957	0.5512
6g4k	958	0.5493
1st3	959	0.5470
7rfa	960	0.5467
6lwz	961	0.5438
7y13	962	0.5412
7k1m	963	0.5410
6m5c	964	0.5406
1arb	965	0.5401
7ay8	966	0.5367

Pdb ID	Rank	PV (avg)
7cz6	967	0.5338
6vk9	968	0.5328
4tpy	969	0.5323
6gv9	970	0.5198
6nox	971	0.5197
8ar3	972	0.5187
6l95	973	0.5175
7osc	974	0.5113
6vhj	975	0.5113
2alg	976	0.5085
6nav	977	0.5040
7p4x	978	0.4938
5zv6	979	0.4797
7my8	980	0.4763
7ns1	981	0.4721
6tuq	982	0.4682
2sfa	983	0.4618
7css	984	0.4606
1xnd	985	0.4550
6imh	986	0.4517
7o2k	987	0.4498
6otb	988	0.4496
7p3m	989	0.4444
6por	990	0.4360
7czj	991	0.4343
7cog	992	0.4324
1thm	993	0.4315
6q1x	994	0.4215
8cio	995	0.4097
8hjd	996	0.4067
4om5	997	0.3969
8ar0	998	0.3697
7l7a	999	0.2990
6u7w	1000	0.2720

Figure S1. 1000 proteins binned based on their function.

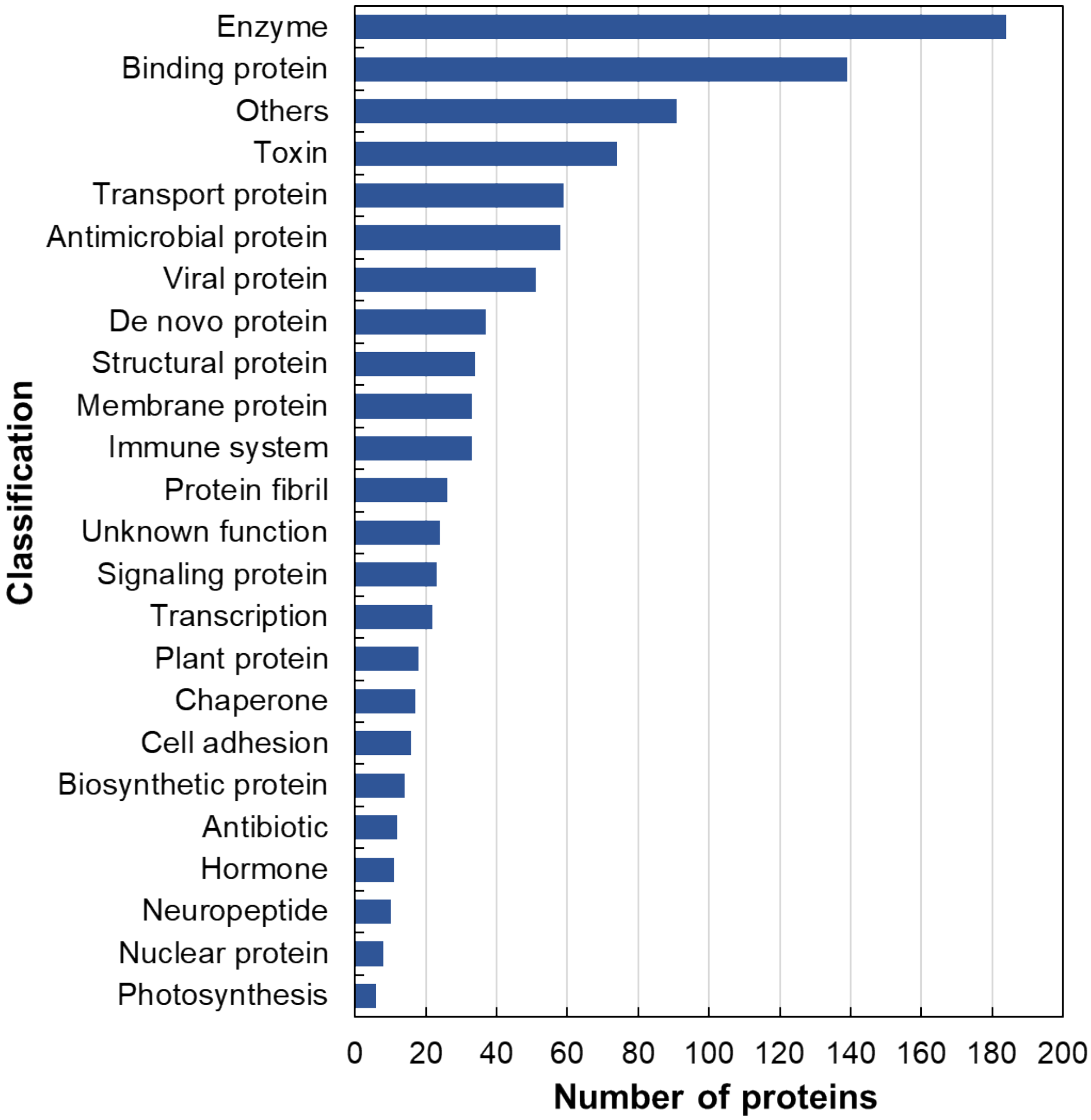

Figure S2. Cluster analysis of 277,877 residues in 1000 proteins.

Shell (teal), mantle (pink), and core (gray) clustering is based on two criteria: (i) the number of three-dimensional neighbors (x-axis) of a residue and (ii) the number of contacting water molecules (y-axis). The dashed lines show the zone boundaries. Based on these criteria, 277,877 residues were classified into one of three zones—54855 (shell), 110348 (mantle), and 112674 (core).

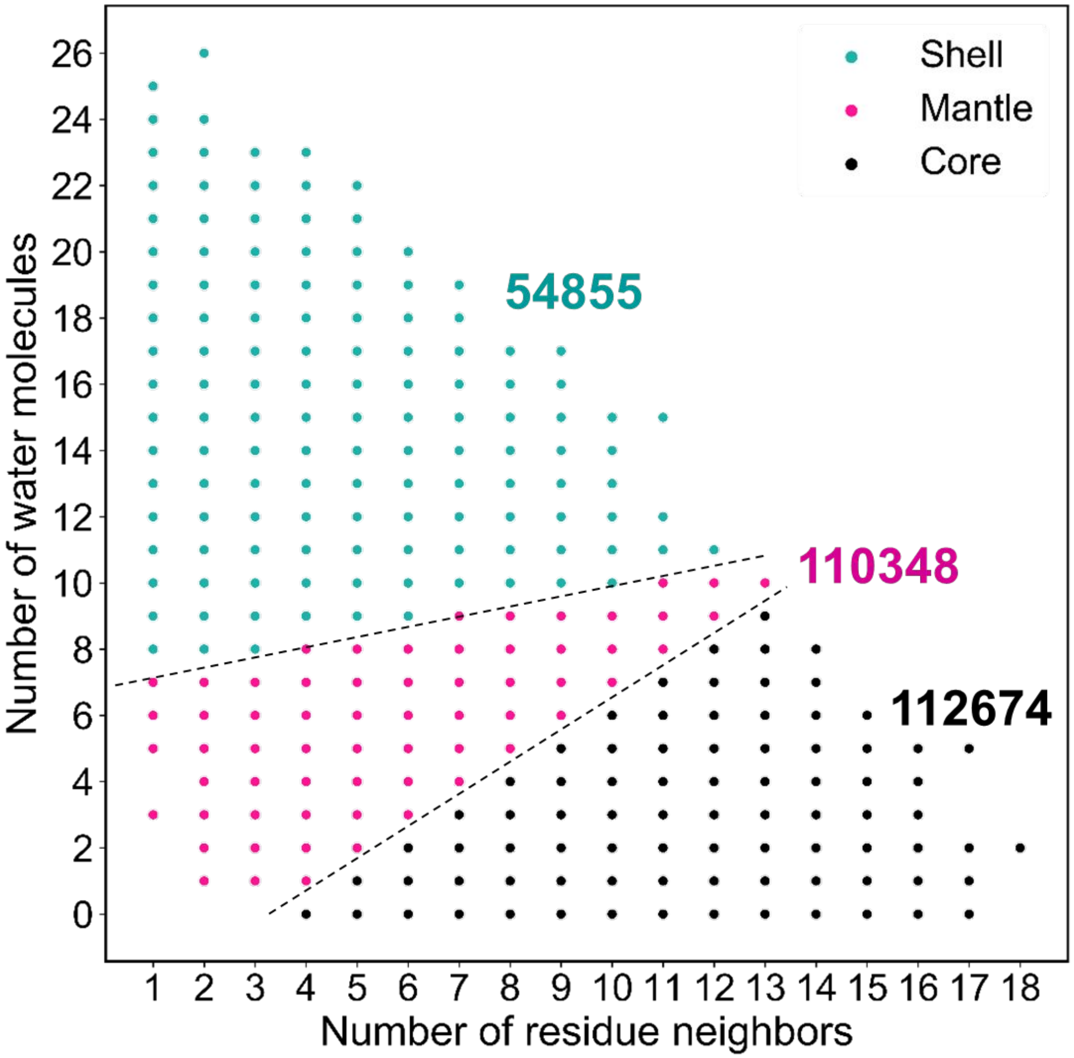

Figure S3. The probability density distributions of the parch values in the three zones. The sample mean $\overline{x}$ and sample standard deviation (s) for the three zones were 0.063 ±0.028, 0.312 ± 0.092, and 0.890 ± 0.190 for the core (gray), mantle (pink), and shell (teal) regions, respectively. The distributions were found to be non-Gaussian in all three cases.

Analysis of the sample statistics revealed more than 99% of the data was contained in the $[\bar{x} - 3s, \bar{x} + 3s]$ interval. The means of neighboring distributions were outside the $\pm 3s$ range. The student's t-statistics of the distributions revealed that the three distributions are statistically distinct and well-separated. The tscore of the mantle with respect to the core is 8.75, and the tscore of the shell with respect to the mantle is 6.32.

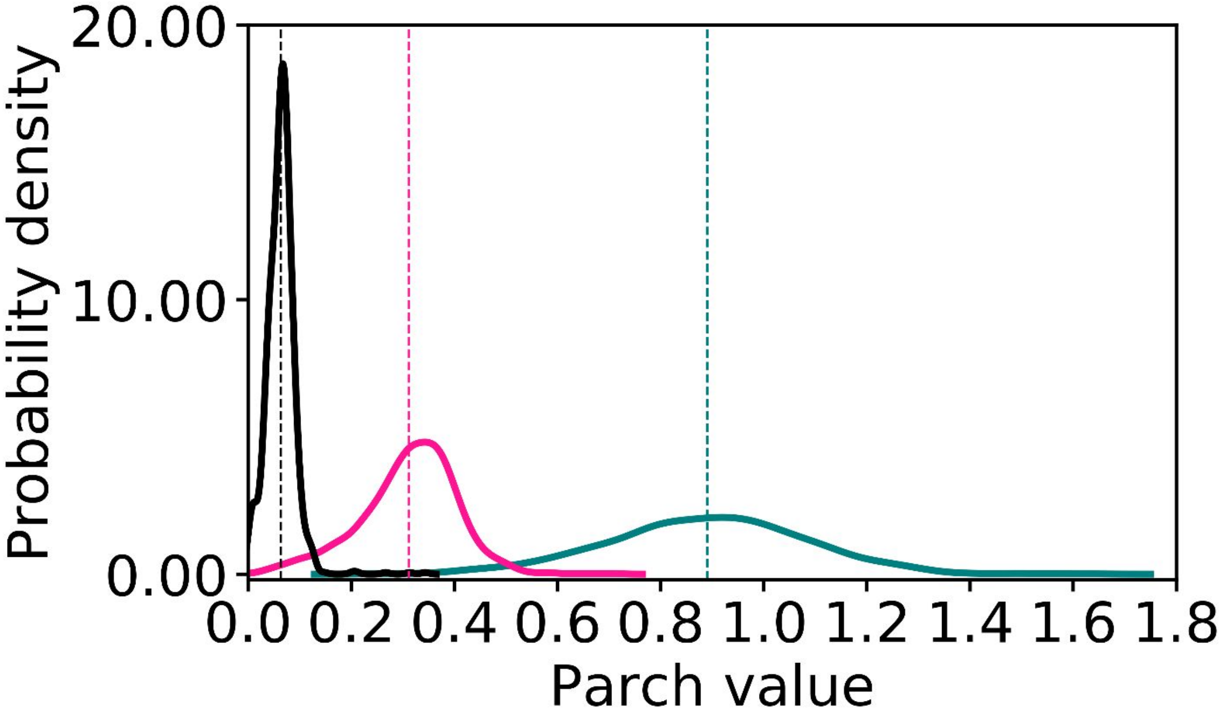

Figure S4. Heat map of residue dyads.
Our analysis revealed that residues tend to colocalize with a preferred topological neighbor. We observed residue pairs or dyads in several proteins, and the statistics are significant as the data is averaged over the 1000 protein data set (Fig. 4). Leucine, the most abundant residue, is prolific in forming dyads, particularly with residues with aliphatic side chains—LI>LV>LA>LL>LF. Another reason why L is less selective could be due to its abundance. However, other aliphatic chain residues V, A, and I are also prolific and nonselective in forming dyads. Among the charged residues, K forms an abundant dyad with E, L, A, and D. Similarly, R forms the most abundant dyad with E. The negatively charged D and E residues form dyads with K and R. The remaining residues form the most abundant dyads with L, except Cystine (C), which forms abundant CC dyads.

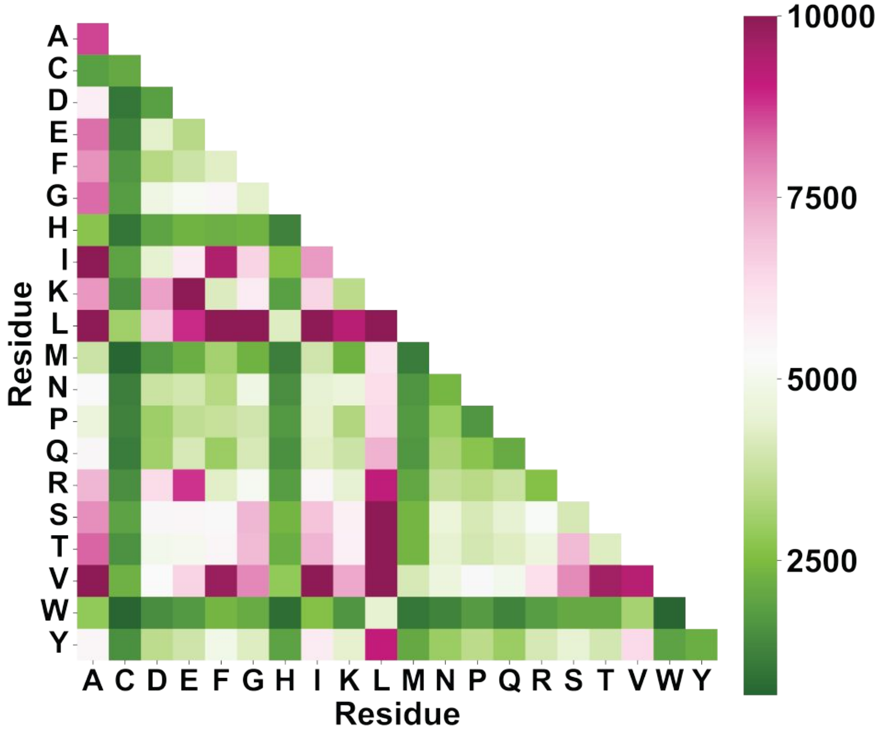

Figure S5. Arginine triads

Heat maps of RXX triads in the surface (left), mantle (center), and core (right) zones of the 1000 protein data set, where X represents 20 amino acids on the x- or y-axis.

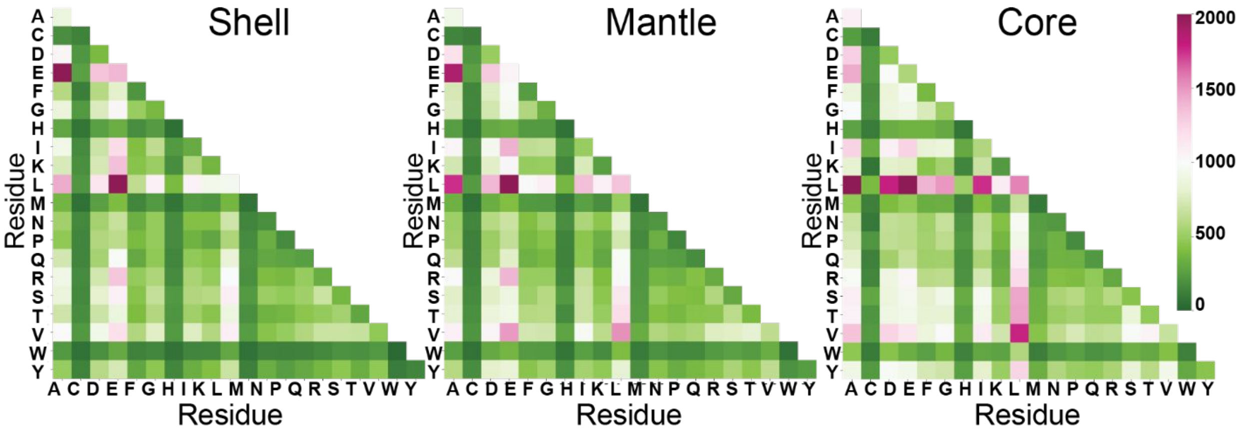

Figure S6. Aspartate triads

Heat maps of DXX triads in the surface (left), mantle (center), and core (right) zones of the 1000 protein data set, where X represents 20 amino acids on the x- or y-axis.

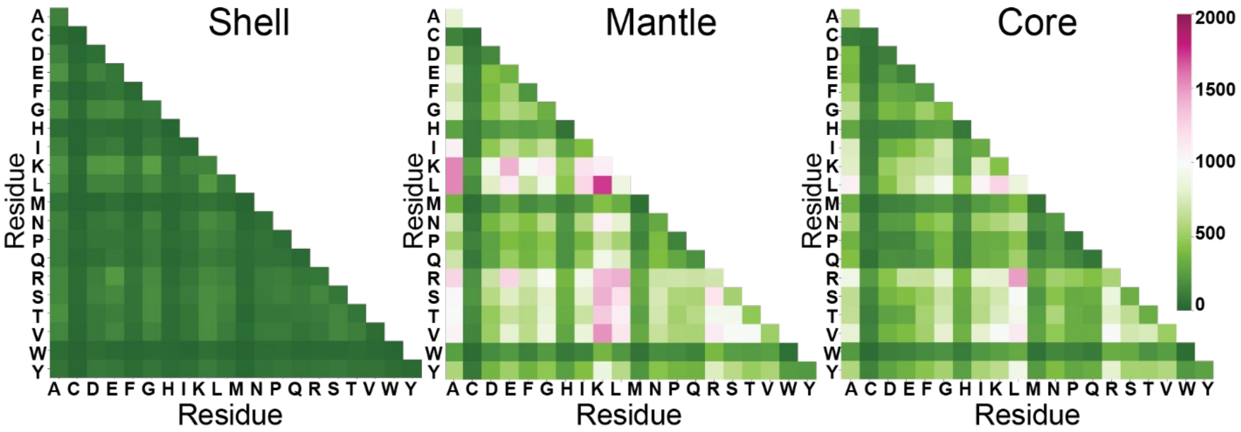

Figure S7. Asparagine triads

Heat maps of NXX triads in the surface (left), mantle (center), and core (right) zones of the 1000 protein data set, where X represents 20 amino acids on the x- or y-axis.

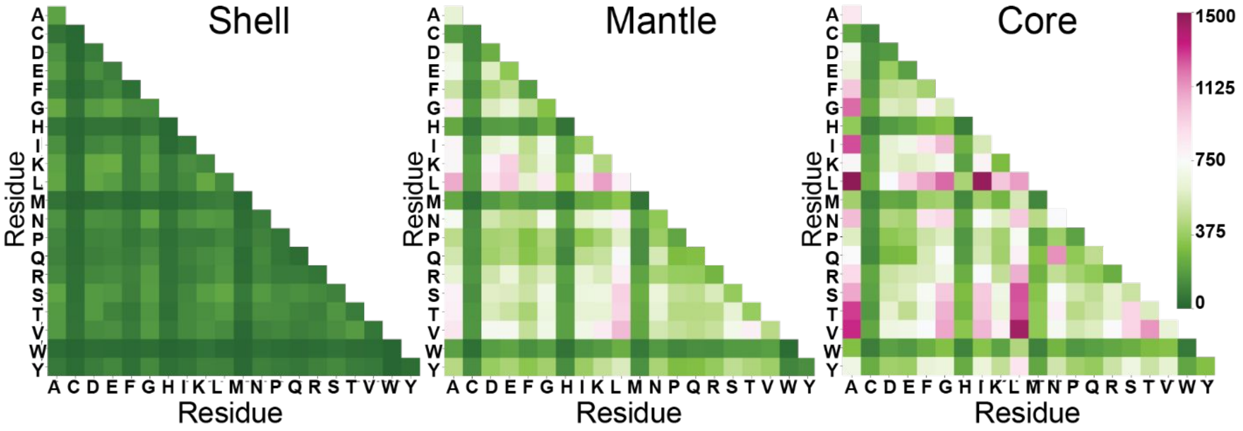

Figure S8. Glutamine triads

Heat maps of QXX triads in the surface (left), mantle (center), and core (right) zones of the 1000 protein data set, where X represents 20 amino acids on the x- or y-axis.

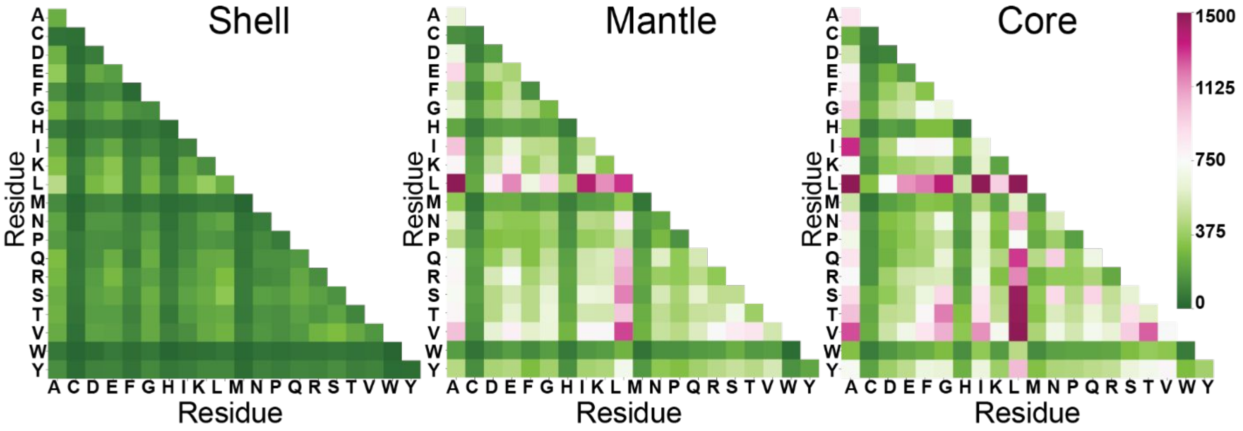

Figure S9. Serine triads

Heat maps of SXX triads in the surface (left), mantle (center), and core (right) zones of the 1000 protein data set, where X represents 20 amino acids on the x- or y-axis.

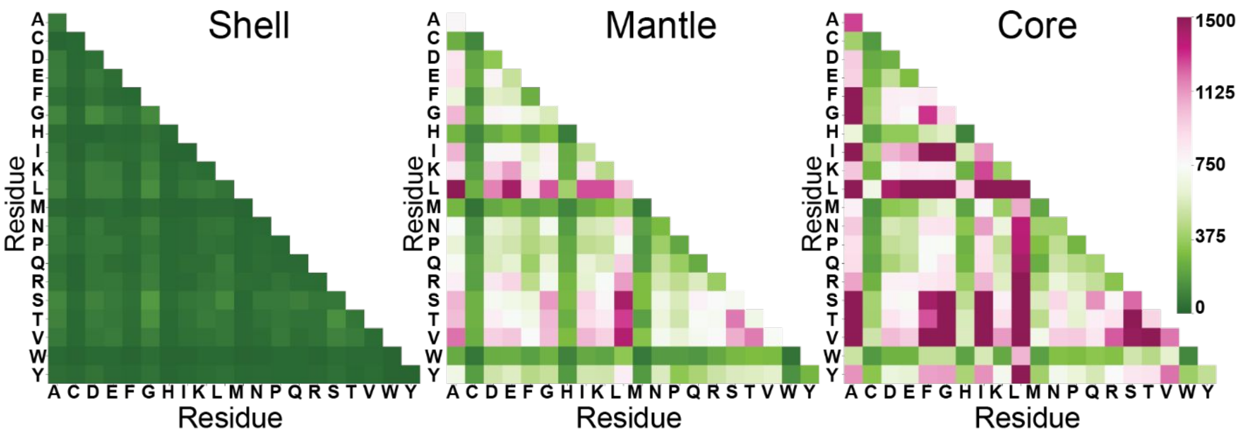

Figure S10. Threonine triads

Heat maps of TXX triads in the surface (left), mantle (center), and core (right) zones of the 1000 protein data set, where X represents 20 amino acids on the x- or y-axis.

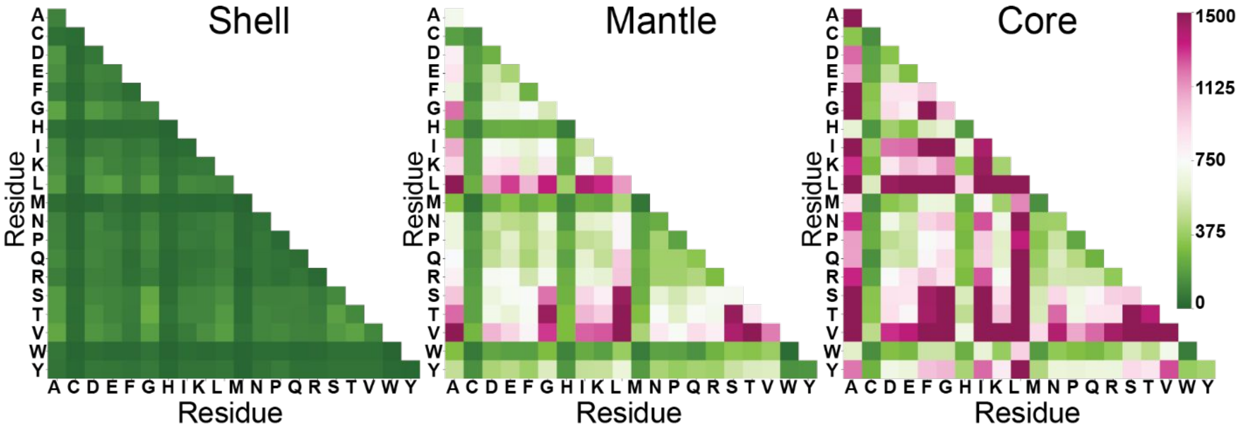

Figure S11. Tyrosine triads

Heat maps of YXX triads in the surface (left), mantle (center), and core (right) zones of the 1000 protein data set, where X represents 20 amino acids on the x- or y-axis.

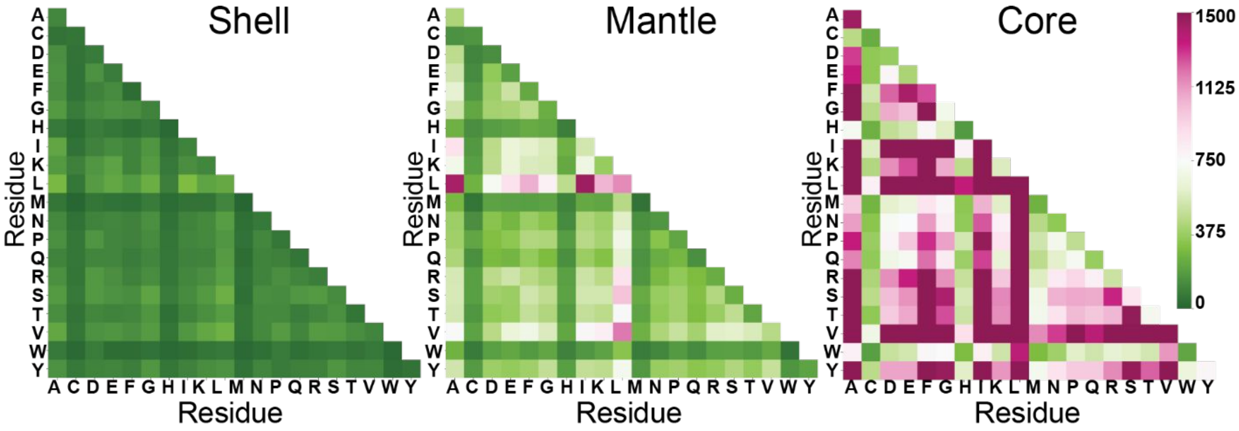

Figure S12. Isoleucine triads

Heat maps of IXX triads in the surface (left), mantle (center), and core (right) zones of the 1000 protein data set, where X represents 20 amino acids on the x- or y-axis.

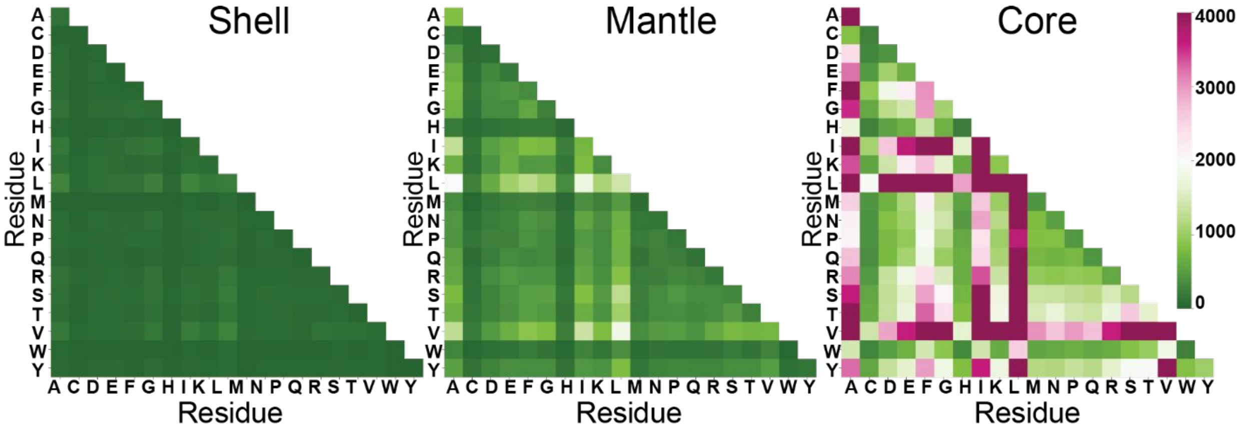

Figure S13. Valine triads

Heat maps of VXX triads in the surface (left), mantle (center), and core (right) zones of the 1000 protein data set, where X represents 20 amino acids on the x- or y-axis.

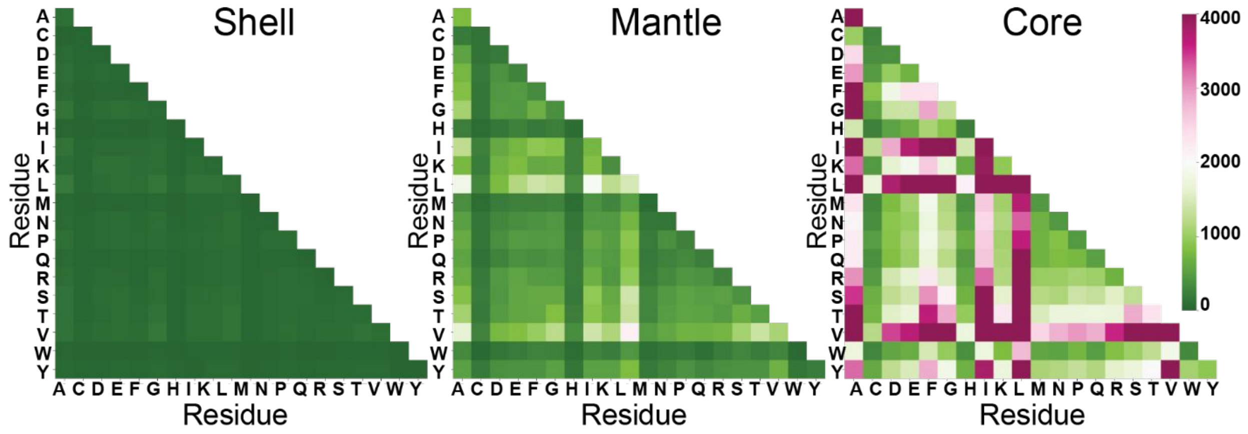

Figure S14. Alanine triads

Heat maps of AXX triads in the surface (left), mantle (center), and core (right) zones of the 1000 protein data set, where X represents 20 amino acids on the x- or y-axis.

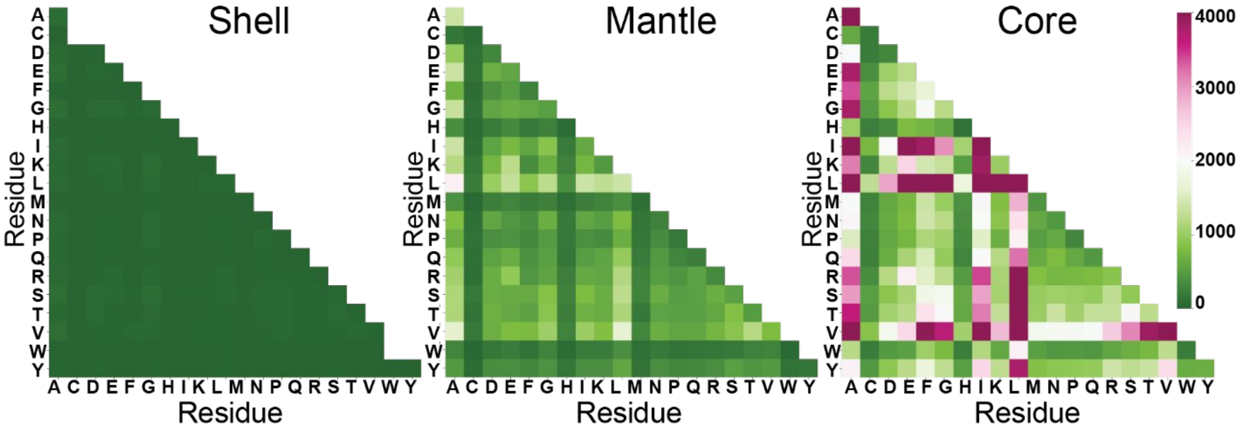

Figure S15. Glycine triads

Heat maps of GXX triads in the surface (left), mantle (center), and core (right) zones of the 1000 protein data set, where X represents 20 amino acids on the x- or y-axis.

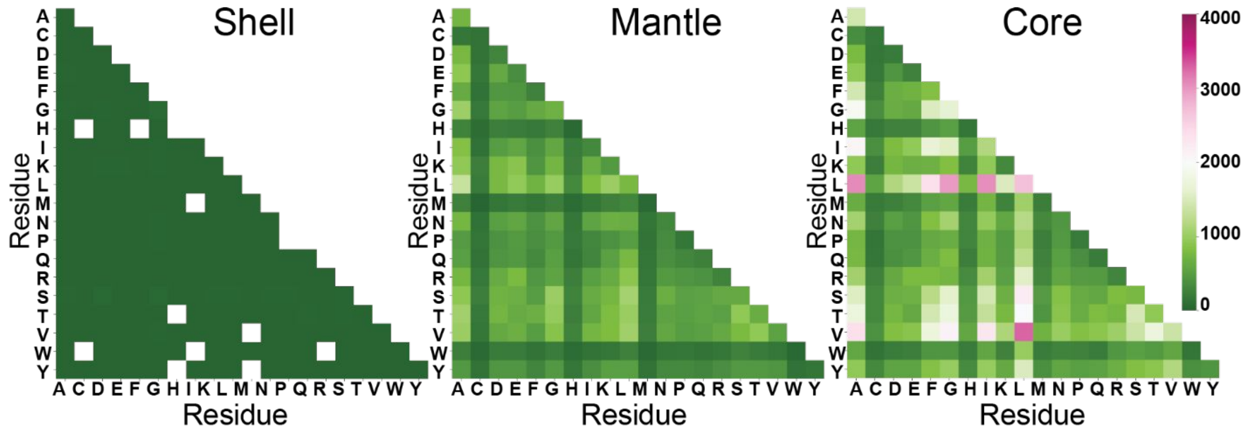

Figure S16. Histidine triads
Heat maps of HXX triads in the surface (left), mantle (center), and core (right) zones of the 1000 protein data set, where X represents 20 amino acids on the x- or y-axis.

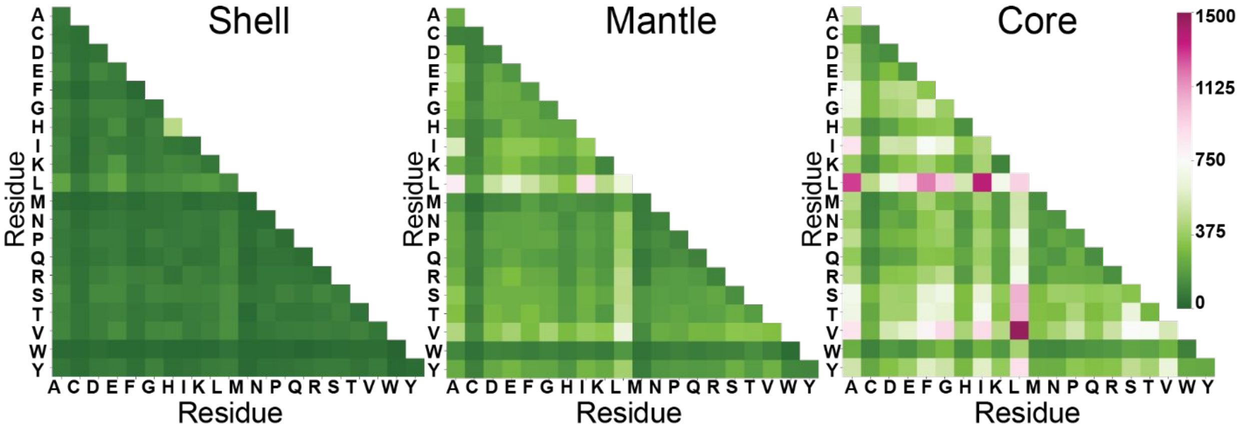

Figure S17. Proline triads

Heat maps of PXX triads in the surface (left), mantle (center), and core (right) zones of the 1000 protein data set, where X represents 20 amino acids on the x- or y-axis.

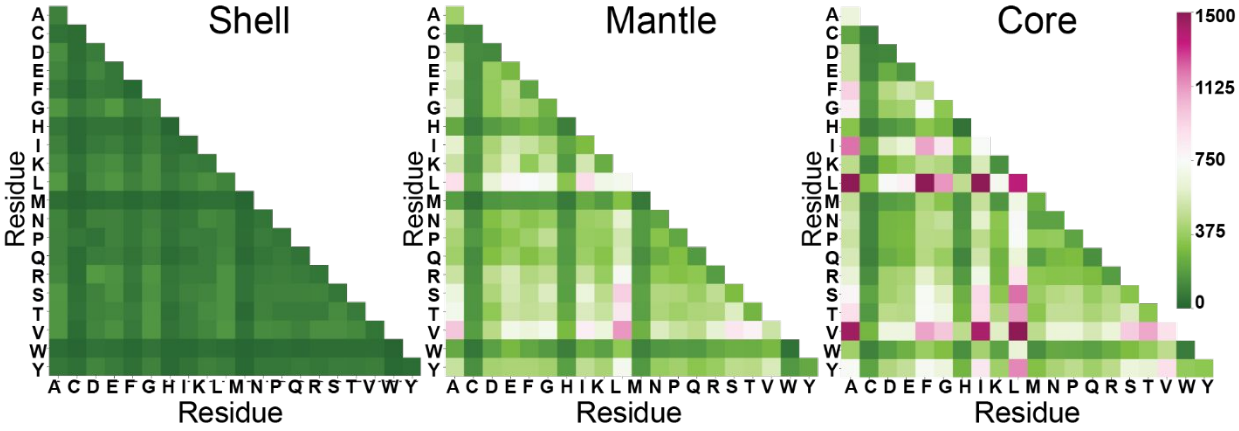

Figure S18. Tryptophan triads

Heat maps of WXX triads in the surface (left), mantle (center), and core (right) zones of the 1000 protein data set, where X represents 20 amino acids on the x- or y-axis.

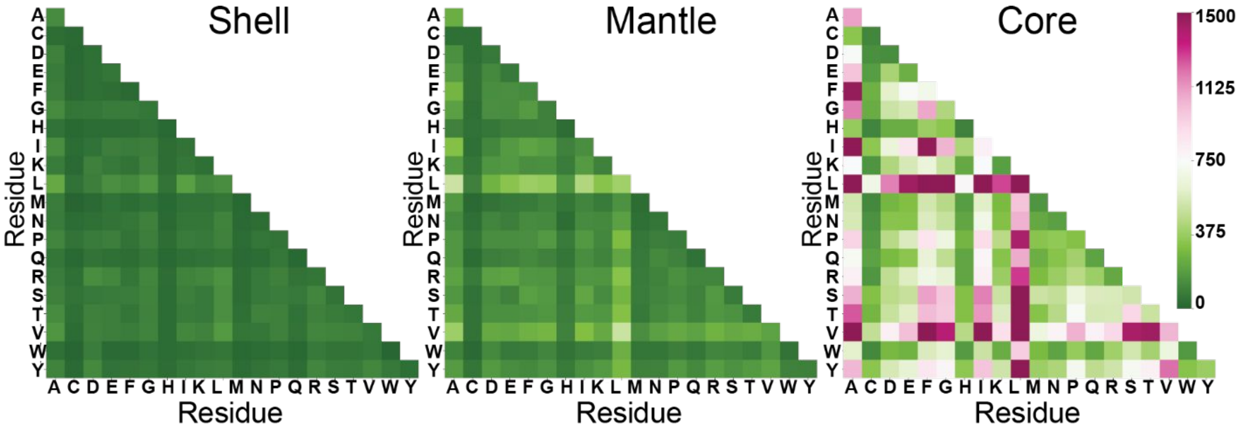

Figure S19. Methionine triads

Heat maps of MXX triads in the surface (left), mantle (center), and core (right) zones of the 1000 protein data set, where X represents 20 amino acids on the x- or y-axis.

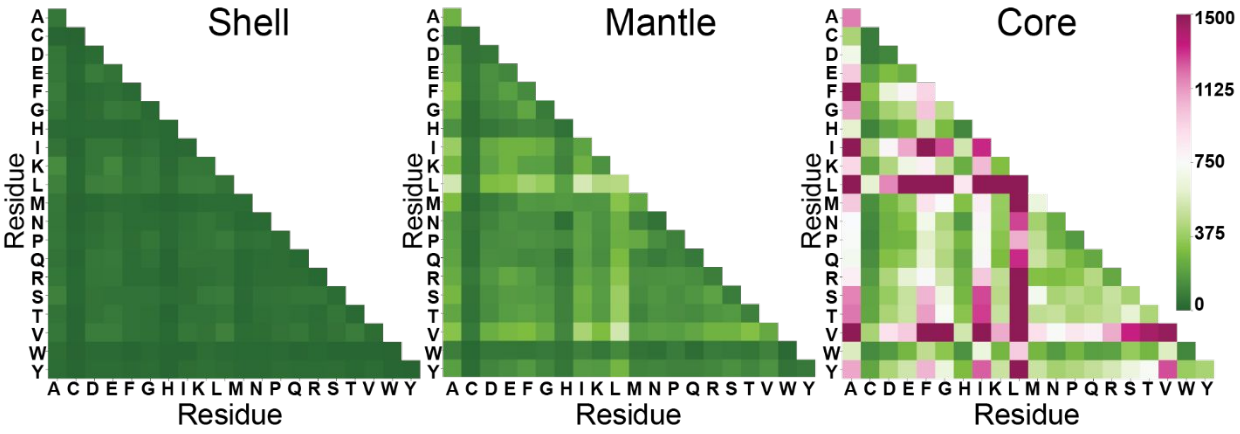

Figure S20. Phenylalanine triads

Heat maps of FXX triads in the surface (left), mantle (center), and core (right) zones of the 1000 protein data set, where X represents 20 amino acids on the x- or y-axis.

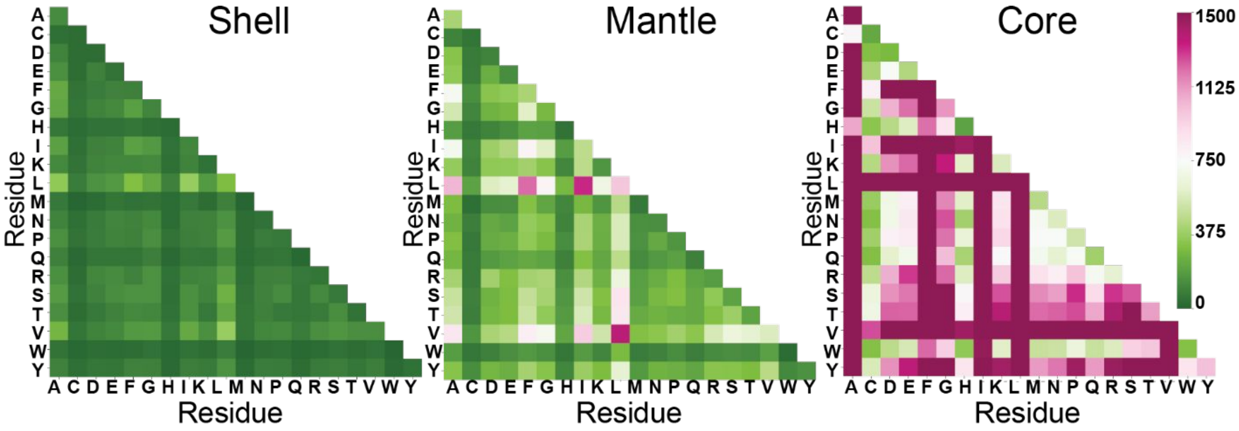

Figure S21. Cysteine triads

Heat maps of CXX triads in the surface (left), mantle (center), and core (right) zones of the 1000 protein data set, where X represents 20 amino acids on the x- or y-axis.

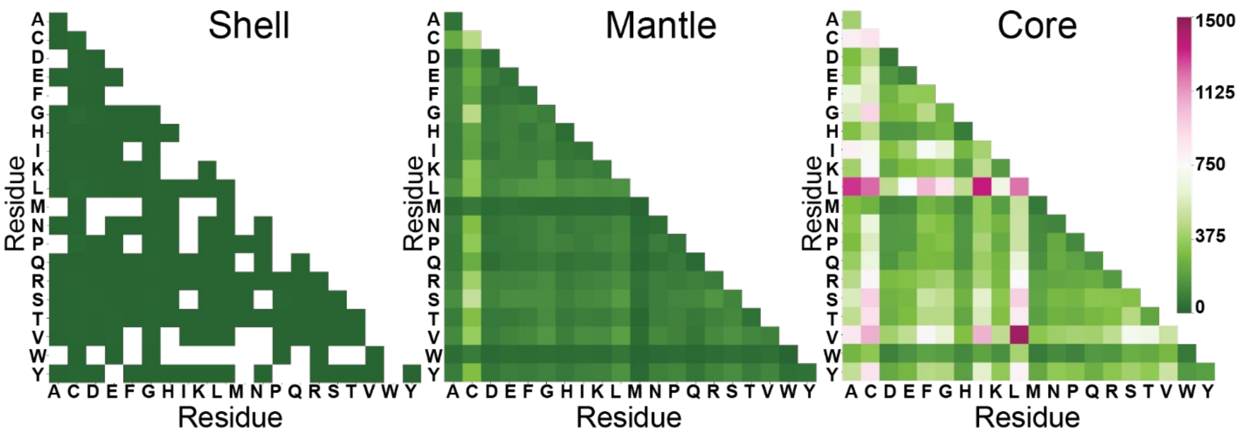

Figure S22. Distribution and parch values of LL0 triads in the shell, mantle, and core regions of proteins.

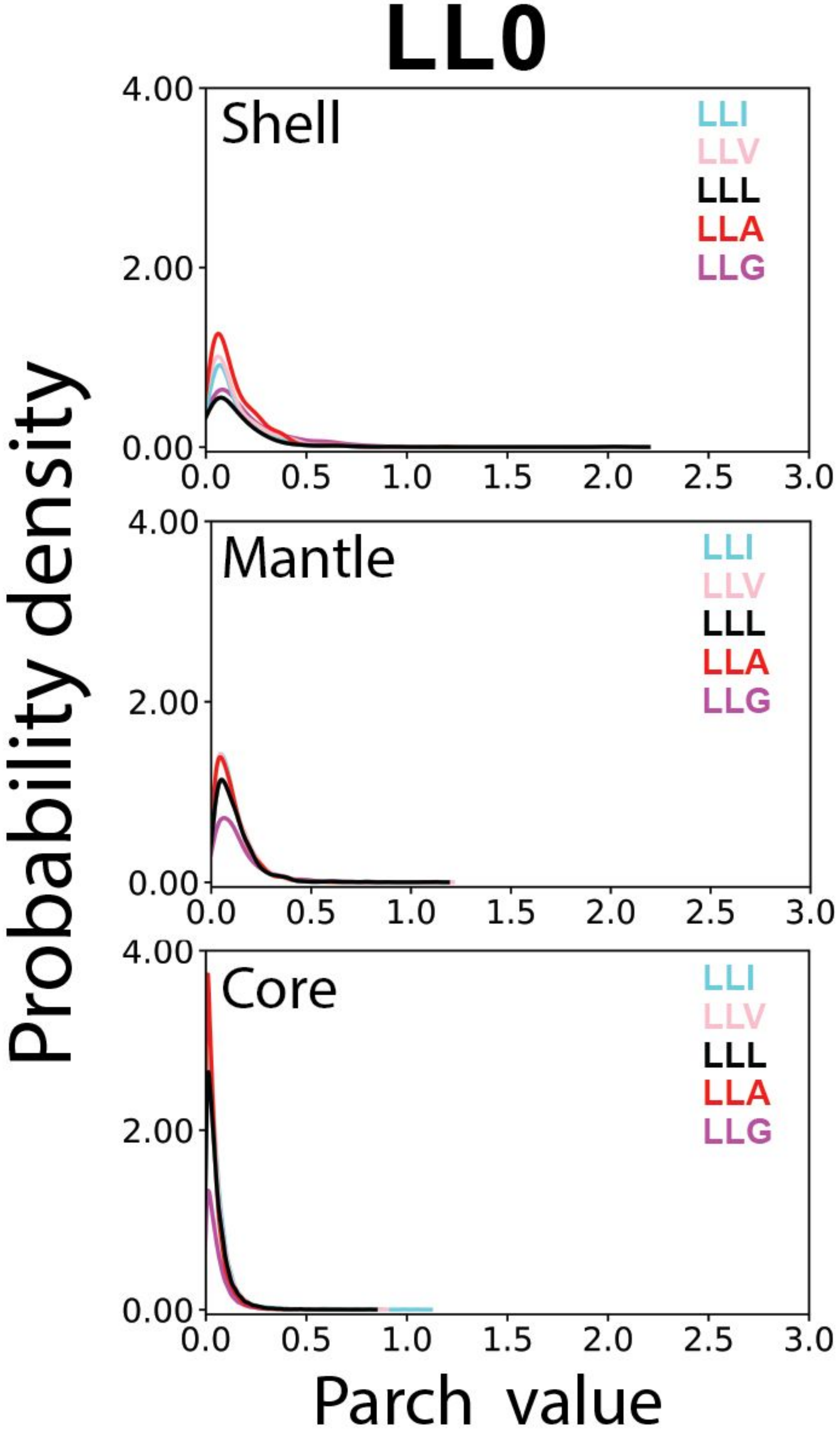

Methods

Protein Equilibration. All-atom molecular dynamics (MD) simulations were performed using the GROMACS software suite (*939*). Each protein was solvated in solution where Na^+ and Cl^- were added to maintain 0.15 M ionic concentration. The CHARMM36-2021 force field parameters (*940*) were used for proteins, ions, and explicit TIP3P water. Each system was simulated using a four-step process: (i) energy minimization using the steepest descent algorithm; (ii) 1 ns isothermal-isochoric (NVT) equilibration using the velocity-rescale thermostat (*941*) with temperature coupling constant τ_t = 1.0 ps; (iii) 1 ns isothermal-isobaric (*NPT*) equilibration with pressure coupling at 1 bar using Berendsen barostat (*942*) with pressure coupling constant τ_p = 5.0 ps and compressibility of 4.5×10^{-5} bar^{-1}; (iv) 10 ns production run with pressure coupling at 1 bar using Parrinello-Rahman barostat (*943*) with τ_p = 5.0 ps and compressibility of 4.5×10^{-5} bar^{-1}. The temperature was maintained at 300 K. The heavy atoms of the proteins were restrained during the *NVT* and *NPT* runs. All restraints were removed during the production run. A 2 fs time step was used, and the nonbonded interaction neighbor list was updated every 20 steps. A 1.2 nm cutoff was used for the electrostatic and van der Waals interactions. The long-range electrostatic interactions were calculated using the Particle-Mesh Ewald (PME) method (*944*) after a 1.2 nm cutoff. The bonds involving hydrogen atoms were constrained using the linear constraint solver (LINCS) algorithm (*945*). Three-dimensional periodic boundary conditions were applied to each system.

PARCH Calculations. The equilibrated protein was extracted and placed into an empty cubic simulation box. The protein was then rehydrated with a shell of water (average density 0.977 g cm^{-3}) of thickness d_{shell} = 0.415 nm larger than the first hydration shell of water from the protein's surface. The edge length (l) of the simulation box is determined by the maximum radius of the protein (d_{max}), the distance of the hydrated counter ions from the protein's surface (d_{ion} = 3.0 nm), and the distance between the ions and the box boundary (d_b = 3.0 nm), according to the equation $l = 2 \times (d_{max} + d_{ion} + d_b)$. The cutoff for calculating the number of water molecules (d_{water}) contacting a residue at any given time is 0.315 nm.

Each system was energy-minimized, followed by water evaporation using the PARCH annealing protocol (*946*), in which water is annealed from 300 to 800 K at constant volume with an annealing rate of 1 K/10 ps. With the increase in temperature, water molecules evaporate from the protein surface and move within the fixed volume of the box without any positional constraints. VMD (*947*) is used to visualize the annealing process. However, the protein was position-restrained with a force constant of 1000 kJ mol^{-1} nm^{-2} to maintain the protein's conformation and topography. Similarly, the counter ions were restrained with force constant of 1000 kJ mol^{-1} nm^{-2} to maintain a constant charge balance around the protein. The parch calculation was performed in triplicates.

K-means Clustering. The clustering of the residues was based on two parameters: (i) the number of neighbors within 3.0 Å from any atom in the residue and (ii) the number of waters contacting the residue at t = 0, d^i_{water}, in the parch simulation process. Using these data for all 277,877 residues in the 1000 protein data set, clustering was performed in the Python programming language (*948*). We used *sklearn.cluster.KMeans* from the community-built machine learning library Scikit-learn (*949*).

To find the optimal number of clusters, we first used the elbow method with the number of clusters (N) from 1 to 20. We calculated the WCSS (Within-Cluster Sum of Square) for each cluster (Figure 1A), which indicates the elbow at N = 3. To further validate the optimal value of N, we performed the Silhouette analysis (*950*) shown in Figure 1B. The Silhouette scores for N = 2 and N = 3 (Figure 1C) show that the coefficient values in both cases exceed the average silhouette score (red dash line), and either value can be used for further analysis. To give a more detailed description of the topographical features of protein residues, we used N=3 clusters labeled as shell, mantle, and core.

Figure S23. K-means clustering.

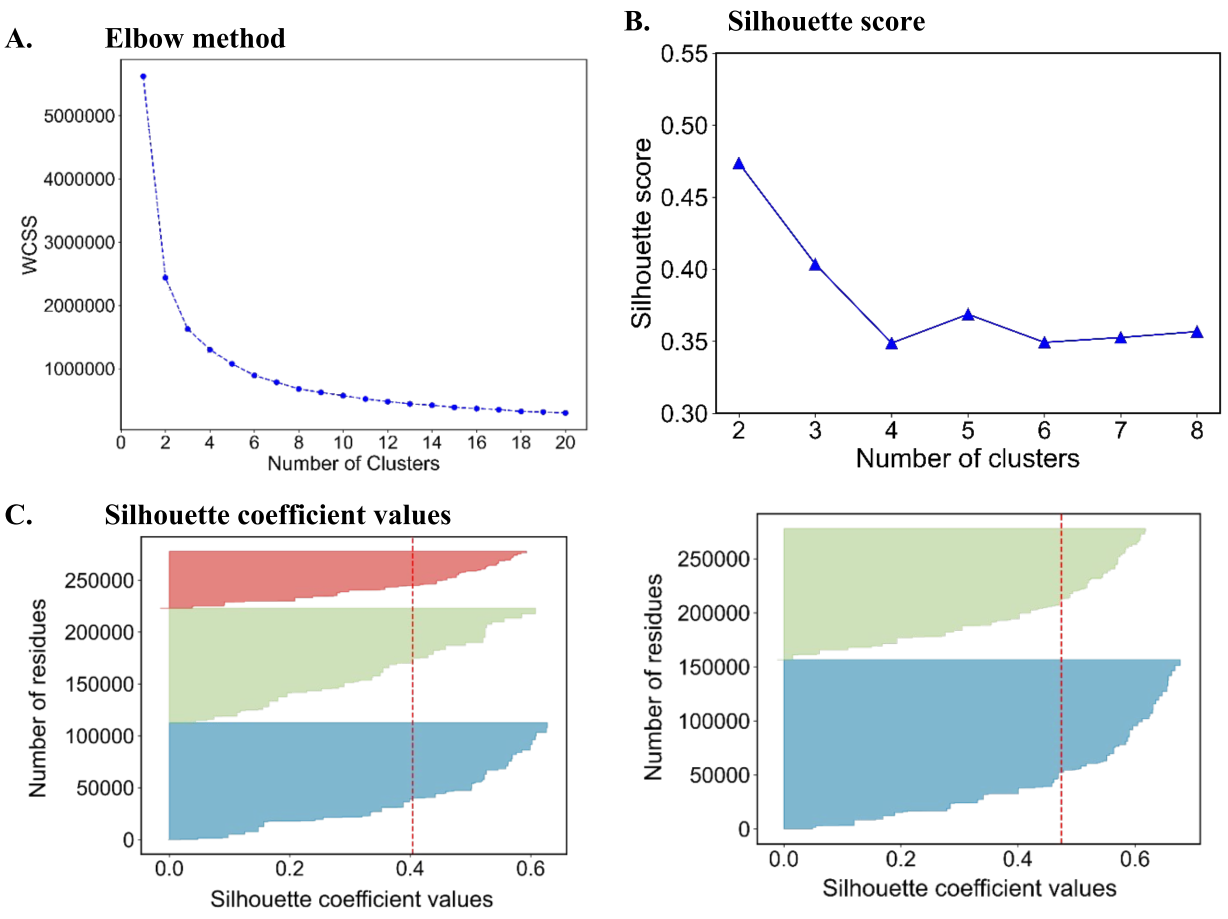

PARCH Output and Visualization. Molecular visualization and images were rendered in PyMol (*951*). The computed parch values for each residue are written in a text file. To facilitate visualization in software suites like PyMOL, the parch values are also written in the occupancy column of an output PDB file.

Data analysis and plotting were performed using in-house Python scripts based on publicly hosted python packages, such as matplotlib (*952*), seaborn (*953*), and MDAnalysis (*954*).

REFERENCES

25. L. H. Weaver, M. G. Grütter, B. W. Matthews, The refined structures of goose lysozyme and its complex with a bound trisaccharide show that the "Goose-type" ly'sozymes lack a catalytic aspartate residue. *Journal of Molecular Biology* **245**, 54-68 (1995).
26. B. W. Segelke, D. Nguyen, R. Chee, N. H. Xuong, E. A. Dennis, Structures of two novel crystal forms of Naja naja naja phospholipase A2 lacking Ca2+ reveal trimeric packing11Edited by I. A. Wilson. *Journal of Molecular Biology* **279**, 223-232 (1998).
27. R. Durley, L. Chen, F. Scott Mathews, L. W. Lim, V. L. Davidson, Crystal structure analysis of amicyanin and apoamicyanin from paracoccus denitrificans at 2.0 Å and 1.8 Å resolution. *Protein Science* **2**, 739-752 (1993).
28. G. D. Smith *et al.*, Ab Initio Structure Determination and Refinement of a Scorpion Protein Toxin. *Acta Crystallographica Section D* **53**, 551-557 (1997).
29. K. R. Acharya, D. I. Stuart, N. P. C. Walker, M. Lewis, D. C. Phillips, Refined structure of baboon α-lactalbumin at 1.7 Å resolution: Comparison with C-type lysozyme. *Journal of Molecular Biology* **208**, 99-127 (1989).
30. S. Tsunasawa, T. Masaki, M. Hirose, M. Soejima, F. Sakiyama, The primary structure and structural characteristics of Achromobacter lyticus protease I, a lysine-specific serine protease. *The Journal of biological chemistry* **264**, 3832-3839 (1989).
31. W. D. Clarkson *et al.*, Nuclear protein import is decreased by engineered mutants of nuclear transport factor 2 (NTF2) that do not bind GDP-Ran11Edited by I. B. Holland. *Journal of Molecular Biology* **272**, 716-730 (1997).
32. B. Favier-Perron, A. Lewit-Bentley, F. Russo-Marie, The High-Resolution Crystal Structure of Human Annexin III Shows Subtle Differences with Annexin V. *Biochemistry* **35**, 1740-1744 (1996).
33. R. Baumgartner *et al.*, Structure of human cyclin-dependent kinase inhibitor p19INK4d: comparison to known ankyrin-repeat-containing structures and implications for the dysfunction of tumor suppressor p16INK4a. *Structure* **6**, 1279-1290 (1998).
34. C. C. S. Deivanayagam, M. Carson, A. Thotakura, S. V. L. Narayana, R. S. Chodavarapu, Structure of FKBP12.6 in complex with rapamycin. *Acta Crystallographica Section D* **56**, 266-271 (2000).
35. L. s. Bellsolell, J. Prieto, L. Serrano, M. Coll, Magnesium Binding to the Bacterial Chemotaxis Protein CheY Results in Large Conformational Changes Involving its Functional Surface. *Journal of Molecular Biology* **238**, 489-495 (1994).
36. A. E. Aleshin *et al.*, Crystal structures of mutant monomeric hexokinase I reveal multiple ADP binding sites and conformational changes relevant to allosteric regulation11Edited by P. E. Wright. *Journal of Molecular Biology* **296**, 1001-1015 (2000).
37. J. F. Davies, II *et al.*, Crystal structures of recombinant human dihydrofolate reductase complexed with folate and 5-deazafolate. *Biochemistry* **29**, 9467-9479 (1990).
38. X. Huang *et al.*, Structure of a WW domain containing fragment of dystrophin in complex with β-dystroglycan. *Nature Structural Biology* **7**, 634-638 (2000).
39. G. T. Montelione *et al.*, Solution structure of murine epidermal growth factor determined by NMR spectroscopy and refined by energy minimization with restraints. *Biochemistry* **31**, 236-249 (1992).
40. X. Ding, B. F. Rasmussen, G. A. Petsko, D. Ringe, Direct Structural Observation of an Acyl-Enzyme Intermediate in the Hydrolysis of an Ester Substrate by Elastase. *Biochemistry* **33**, 9285-9293 (1994).

41. J. M. Jez, M. E. Bowman, R. A. Dixon, J. P. Noel, Structure and mechanism of the evolutionarily unique plant enzyme chalcone isomerase. *Nature Structural Biology* **7**, 786-791 (2000).
42. F. J. Moy *et al.*, NMR Structure of Free RGS4 Reveals an Induced Conformational Change upon Binding Gα. *Biochemistry* **39**, 7063-7073 (2000).
43. M. H. le Du, P. Marchot, P. E. Bougis, J. C. Fontecilla-Camps, 1.9-A resolution structure of fasciculin 1, an anti-acetylcholinesterase toxin from green mamba snake venom. *The Journal of biological chemistry* **267**, 22122-22130 (1992).
44. 1ff4. https://www.rcsb.org/structure/1FF4.
45. C. D. Dickinson *et al.*, Crystal structure of the tenth type III cell adhesion module of human fibronectin. *Journal of Molecular Biology* **236**, 1079-1092 (1994).
46. S. Iyer *et al.*, The Crystal Structure of Human Placenta Growth Factor-1 (PlGF-1), an Angiogenic Protein, at 2.0 Å Resolution*. *Journal of Biological Chemistry* **276**, 12153-12161 (2001).
47. K. Sasaki, S. Dockerill, D. A. Adamiak, I. J. Tickle, T. Blundell, X-ray analysis of glucagon and its relationship to receptor binding. *Nature* **257**, 751-757 (1975).
48. R. Bayliss *et al.*, Structural basis for the interaction between NTF2 and nucleoporin FxFG repeats. *The EMBO Journal* **21**, 2843-2853 (2002).
49. B. Xiao, G. Shi, X. Chen, H. Yan, X. Ji, Crystal structure of 6-hydroxymethyl-7,8-dihydropterin pyrophosphokinase, a potential target for the development of novel antimicrobial agents. *Structure* **7**, 489-496 (1999).
50. J. H. Lee, K. Z. Chang, V. Patel, C. J. Jeffery, Crystal Structure of Rabbit Phosphoglucose Isomerase Complexed with Its Substrate d-Fructose 6-Phosphate. *Biochemistry* **40**, 7799-7805 (2001).
51. G. Precigoux *et al.*, A crystallographic study of haem binding to ferritin. *Acta Crystallographica Section D* **50**, 739-743 (1994).
52. K. Briknarová *et al.*, Structural analysis of BAG1 cochaperone and its interactions with Hsc70 heat shock protein. *Nature Structural Biology* **8**, 349-352 (2001).
53. J.-O. Lee, P. Rieu, M. A. Arnaout, R. Liddington, Crystal structure of the A domain from the a subunit of integrin CR3 (CD11 b/CD18). *Cell* **80**, 631-638 (1995).
54. P. Bellosta *et al.*, Identification of Receptor and Heparin Binding Sites in Fibroblast Growth Factor 4 by Structure-Based Mutagenesis. *Molecular and Cellular Biology* **21**, 5946-5957 (2001).
55. B. Stec, A. Yamano, M. Whitlow, M. M. Teeter, Structure of Human Plasminogen Kringle 4 at 1.68 A and 277 K. A Possible Structural Role of Disordered Residues. *Acta Crystallographica Section D* **53**, 169-178 (1997).
56. P. A. Del Rizzo, Y. Bi, S. D. Dunn, B. H. Shilton, The "Second Stalk" of Escherichia coli ATP Synthase: Structure of the Isolated Dimerization Domain. *Biochemistry* **41**, 6875-6884 (2002).
57. D. Samuel, Y.-J. Liu, C.-S. Cheng, P.-C. Lyu, Solution Structure of Plant Nonspecific Lipid Transfer Protein-2 from Rice (Oryza sativa)*. *Journal of Biological Chemistry* **277**, 35267-35273 (2002).
58. P. J. Artymiuk, C. C. F. Blake, Refinement of human lysozyme at 1.5 Å resolution analysis of non-bonded and hydrogen-bond interactions. *Journal of Molecular Biology* **152**, 737-762 (1981).

59. C. Qian, Y. Yao, Y. Tong, J. Wang, W. Tang, Structural analysis of zinc-substituted cytochrome c. *JBIC Journal of Biological Inorganic Chemistry* **8**, 394-400 (2003).
60. 1mbd. https://www.rcsb.org/structure/1MBD.
61. D. H. Shin, J. Y. Lee, K. Y. Hwang, K. Kyu Kim, S. W. Suh, High-resolution crystal structure of the non-specific lipid-transfer protein from maize seedlings. *Structure* **3**, 189-199 (1995).
62. N. Friedland, H.-L. Liou, P. Lobel, A. M. Stock, Structure of a cholesterol-binding protein deficient in Niemann–Pick type C2 disease. *Proceedings of the National Academy of Sciences* **100**, 2512-2517 (2003).
63. C. Ban, B. Ramakrishnan, K.-Y. Ling, C. Kung, M. Sundaralingam, Structure of the recombinant Paramecium tetraurelia calmodulin at 1.68 A resolution. *Acta Crystallographica Section D* **50**, 50-63 (1994).
64. D. L. Scott *et al.*, Structures of Free and Inhibited Human Secretory Phospholipase A₂ from Inflammatory Exudate. *Science* **254**, 1007-1010 (1991).
65. D. Picot, P. J. Loll, R. M. Garavito, The X-ray crystal structure of the membrane protein prostaglandin H2 synthase-1. *Nature* **367**, 243-249 (1994).
66. M. Fujinaga, M. M. Chernaia, S. C. Mosimann, M. N. G. James, N. I. Tarasova, Crystal structure of human pepsin and its complex with pepstatin. *Protein Science* **4**, 960-972 (1995).
67. D. P. Barondeau, C. D. Putnam, C. J. Kassmann, J. A. Tainer, E. D. Getzoff, Mechanism and energetics of green fluorescent protein chromophore synthesis revealed by trapped intermediate structures. *Proceedings of the National Academy of Sciences* **100**, 12111-12116 (2003).
68. X. Zhang, L. Chen, D. P. Bancroft, C. K. Lai, T. E. Maione, Crystal Structure of Recombinant Human Platelet Factor 4. *Biochemistry* **33**, 8361-8366 (1994).
69. F. R. Ahmed, D. R. Rose, S. V. Evans, M. E. Pippy, R. To, Refinement of Recombinant Oncomodulin at 1·30 Å Resolution. *Journal of Molecular Biology* **230**, 1216-1224 (1993).
70. D. Pantoja-Uceda *et al.*, Solution Structure of a Methionine-Rich 2S Albumin from Sunflower Seeds: Relationship to Its Allergenic and Emulsifying Properties. *Biochemistry* **43**, 6976-6986 (2004).
71. D. W. Goddette *et al.*, The crystal structure of the Bacillus lentus alkaline protease, subtilisin BL, at 1.4 Å resolution. *Journal of Molecular Biology* **228**, 580-595 (1992).
72. C. Toyoshima, M. Nakasako, H. Nomura, H. Ogawa, Crystal structure of the calcium pump of sarcoplasmic reticulum at 2.6 Å resolution. *Nature* **405**, 647-655 (2000).
73. D. J. Leahy, W. A. Hendrickson, l. Aukhil, H. P. Erickson, Structure of a Fibronectin Type III Domain from Tenascin Phased by MAD Analysis of the Selenomethionyl Protein. *Science* **258**, 987-991 (1992).
74. A. V. Teplyakov *et al.*, Crystal structure of thermitase at 1.4A˚resolution. *Journal of Molecular Biology* **214**, 261-279 (1990).
75. R. Natesh *et al.*, Crystal structure at 1.8 Å resolution and proposed amino acid sequence of a thermostable xylanase from Thermoascus aurantiacus11Edited by A. Klug. *Journal of Molecular Biology* **288**, 999-1012 (1999).
76. S. Vijay-Kumar, C. E. Bugg, W. J. Cook, Structure of ubiquitin refined at 1.8Åresolution. *Journal of Molecular Biology* **194**, 531-544 (1987).

77. T. M. T. Hall, J. A. Porter, P. A. Beachy, D. J. Leahy, A potential catalytic site revealed by the 1.7-Å crystal structure of the amino-terminal signalling domain of Sonic hedgehog. *Nature* **378**, 212-216 (1995).
78. L. M. Henriksson, P. Johansson, T. Unge, S. L. Mowbray, X-ray structure of peptidyl-prolyl cis–trans isomerase A from Mycobacterium tuberculosis. *European Journal of Biochemistry* **271**, 4107-4113 (2004).
79. Y. S. Ho *et al.*, Brain acetylhydrolase that inactivates platelet-activating factor is a G-protein-like trimer. *Nature* **385**, 89-93 (1997).
80. S. M. Dayan, A. Van Donkelaar, A. A. Kortt, Crystallization and preliminary crystallographic data of the major albumin from Psophocarpus tetragonolobus (L.) DC. *The Journal of biological chemistry* **262**, 10287-10289 (1987).
81. 1xnb. *https://www.rcsb.org/structure/1XNB*.
82. 1xnd. *https://www.rcsb.org/structure/1XND*.
83. S. Trakhanov *et al.*, Ligand-Free and -Bound Structures of the Binding Protein (LivJ) of the Escherichia coli ABC Leucine/Isoleucine/Valine Transport System: Trajectory and Dynamics of the Interdomain Rotation and Ligand Specificity. *Biochemistry* **44**, 6597-6608 (2005).
84. M. Kato, T. Mizuno, T. Shimizu, T. Hakoshima, Refined structure of the histidine-containing phosphotransfer (HPt) domain of the anaerobic sensor kinase ArcB from Escherichia coli at 1.57 A resolution. *Acta Crystallographica Section D* **55**, 1842-1849 (1999).
85. G. D. Smith, W. A. Pangborn, R. H. Blessing, The structure of T6 bovine insulin. *Acta Crystallographica Section D* **61**, 1476-1482 (2005).
86. S. D. Patel *et al.*, Type II Cadherin Ectodomain Structures: Implications for Classical Cadherin Specificity. *Cell* **124**, 1255-1268 (2006).
87. S. Wilkens, D. Borchardt, J. Weber, A. E. Senior, Structural Characterization of the Interaction of the δ and α Subunits of the Escherichia coli F1F0-ATP Synthase by NMR Spectroscopy. *Biochemistry* **44**, 11786-11794 (2005).
88. N. Pasquato *et al.*, Crystal Structure of Peach Pru p 3, the Prototypic Member of the Family of Plant Non-specific Lipid Transfer Protein Pan-allergens. *Journal of Molecular Biology* **356**, 684-694 (2006).
89. T. Fossen *et al.*, Solution Structure of the Human Immunodeficiency Virus Type 1 p6 Protein*. *Journal of Biological Chemistry* **280**, 42515-42527 (2005).
90. A. M. Stock *et al.*, Structure of the magnesium-bound form of CheY and mechanism of phosphoryl transfer in bacterial chemotaxis. *Biochemistry* **32**, 13375-13380 (1993).
91. H. Ke, Similarities and differences between human cyclophilin A and other β-barrel structures: Structural refinement at 1.63 Å resolution. *Journal of Molecular Biology* **228**, 539-550 (1992).
92. J. Fort *et al.*, The Structure of Human 4F2hc Ectodomain Provides a Model for Homodimerization and Electrostatic Interaction with Plasma Membrane*. *Journal of Biological Chemistry* **282**, 31444-31452 (2007).
93. M. Senda *et al.*, Molecular Mechanism of the Redox-dependent Interaction between NADH-dependent Ferredoxin Reductase and Rieske-type [2Fe-2S] Ferredoxin. *Journal of Molecular Biology* **373**, 382-400 (2007).

94. H. Yagi *et al.*, Structures of the thermophilic F₁-ATPase ε subunit suggesting ATP-regulated arm motion of its C-terminal domain in F₁. *Proceedings of the National Academy of Sciences* **104**, 11233-11238 (2007).
95. K. Morikawa *et al.*, Crystal structure of a pyrimidine dimer-specific excision repair enzyme from bacteriophage T4: Refinement at 1.45 Å and X-ray analysis of the three active site mutants. *Journal of Molecular Biology* **249**, 360-375 (1995).
96. Q.-X. Hua *et al.*, Structure of a protein in a kinetic trap. *Nature Structural Biology* **2**, 129-138 (1995).
97. A. C. Terwisscha van Scheltinga, M. Hennig, B. W. Dijkstra, The 1.8 Å Resolution Structure of Hevamine, a Plant Chitinase/Lysozyme, and Analysis of the Conserved Sequence and Structure Motifs of Glycosyl Hydrolase Family 18. *Journal of Molecular Biology* **262**, 243-257 (1996).
98. E. V. Bocharov *et al.*, Spatial Structure of the Dimeric Transmembrane Domain of the Growth Factor Receptor ErbB2 Presumably Corresponding to the Receptor Active State*. *Journal of Biological Chemistry* **283**, 6950-6956 (2008).
99. J. L. Gifford, H. Ishida, H. J. Vogel, Fast methionine-based solution structure determination of calcium-calmodulin complexes. *Journal of Biomolecular NMR* **50**, 71-81 (2011).
100. A. K. Gizatullina *et al.*, Recombinant production and solution structure of lipid transfer protein from lentil Lens culinaris. *Biochemical and Biophysical Research Communications* **439**, 427-432 (2013).
101. V. R. Bacot-Davis, J. J. Ciomperlik, H. A. Basta, C. C. Cornilescu, A. C. Palmenberg, Solution structures of Mengovirus Leader protein, its phosphorylated derivatives, and in complex with nuclear transport regulatory protein, RanGTPase. *Proceedings of the National Academy of Sciences* **111**, 15792-15797 (2014).
102. 2n1b. https://www.rcsb.org/structure/2N1B.
103. S. Steinbacher, R. Bass, P. Strop, D. C. Rees, in *Current Topics in Membranes*. (Academic Press, 2007), vol. 58, pp. 1-24.
104. S. Al-Karadaghi *et al.*, Refined crystal structure of liver alcohol dehydrogenase-NADH complex at 1.8 A resolution. *Acta Crystallographica Section D* **50**, 793-807 (1994).
105. A. Müller, W. Hinrichs, W. M. Wolf, W. Saenger, Crystal structure of calcium-free proteinase K at 1.5-A resolution. *The Journal of biological chemistry* **269**, 23108-23111 (1994).
106. K. KITADOKORO, H. TSUZUKI, E. NAKAMURA, T. SATO, H. TERAOKA, Purification, characterization, primary structure, crystallization and preliminary crystallographic study of a serine proteinase from Streptomyces fradiae ATCC 14544. *European Journal of Biochemistry* **220**, 55-61 (1994).
107. R. Bott *et al.*, The three-dimensional structure of Bacillus amyloliquefaciens subtilisin at 1.8 A and an analysis of the structural consequences of peroxide inactivation. *The Journal of biological chemistry* **263**, 7895-7906 (1988).
108. S. Sreeramulu *et al.*, The Human Cdc37·Hsp90 Complex Studied by Heteronuclear NMR Spectroscopy*. *Journal of Biological Chemistry* **284**, 3885-3896 (2009).
109. E. Lolis, G. A. Petsko, Crystallographic analysis of the complex between triosephosphate isomerase and 2-phosphoglycolate at 2.5-.ANG. resolution: implications for catalysis. *Biochemistry* **29**, 6619-6625 (1990).

110. H. Yashiroda *et al.*, Crystal structure of a chaperone complex that contributes to the assembly of yeast 20S proteasomes. *Nature Structural & Molecular Biology* **15**, 228-236 (2008).
111. T. Takano, R. E. Dickerson, Redox conformation changes in refined tuna cytochrome c. *Proceedings of the National Academy of Sciences* **77**, 6371-6375 (1980).
112. E. M. Hearn, D. R. Patel, B. W. Lepore, M. Indic, B. van den Berg, Transmembrane passage of hydrophobic compounds through a protein channel wall. *Nature* **458**, 367-370 (2009).
113. J. L. Smith, P. W. R. Corfield, W. A. Hendrickson, B. W. Low, Refinement at 1.4 A resolution of a model of erabutoxin b: treatment of ordered solvent and discrete disorder. *Acta Crystallographica Section A* **44**, 357-368 (1988).
114. I. Van Molle *et al.*, The F4 fimbrial chaperone FaeE is stable as a monomer that does not require self-capping of its pilin-interactive surfaces. *Acta Crystallographica Section D* **65**, 411-420 (2009).
115. J. F. Cramer *et al.*, GGA Autoinhibition Revisited. *Traffic* **11**, 259-273 (2010).
116. J. R. Partridge, T. U. Schwartz, Crystallographic and Biochemical Analysis of the Ran-binding Zinc Finger Domain. *Journal of Molecular Biology* **391**, 375-389 (2009).
117. W. D. Mercer, S. I. Winn, H. C. Watson, Twinning in crystals of human skeletal muscle d-glyceraldehyde-3-phosphate dehydrogenase. *Journal of Molecular Biology* **104**, 277-283 (1976).
118. J. Jung *et al.*, Crystal structure of Clostridium thermocellum ribose-5-phosphate isomerase B reveals properties critical for fast enzyme kinetics. *Applied Microbiology and Biotechnology* **90**, 517-527 (2011).
119. J. Wang *et al.*, Epsin N-terminal homology domains bind on opposite sides of two SNAREs. *Proceedings of the National Academy of Sciences* **108**, 12277-12282 (2011).
120. H. C. Watson *et al.*, Sequence and structure of yeast phosphoglycerate kinase. *The EMBO Journal* **1**, 1635-1640 (1982).
121. A. C. Papageorgiou, H. S. Tranter, K. R. Acharya, Crystal structure of microbial superantigen staphylococcal enterotoxin B at 1.5 Å resolution: implications for superantigen recognition by MHC class II molecules and T-cell receptors11Edited by R. Huber. *Journal of Molecular Biology* **277**, 61-79 (1998).
122. V. M. Girish *et al.*, Identification and Structural Characterization of a New Three-Finger Toxin Hemachatoxin from Hemachatus haemachatus Venom. *PLOS ONE* **7**, e48112 (2012).
123. A. Dalby, Z. Dauter, J. A. Littlechild, Crystal structure of human muscle aldolase complexed with fructose 1,6-bisphosphate: Mechanistic implications. *Protein Science* **8**, 291-297 (1999).
124. Y. Chen *et al.*, The Crystal Structure of Arabidopsis VSP1 Reveals the Plant Class C-Like Phosphatase Structure of the DDDD Superfamily of Phosphohydrolases. *PLOS ONE* **7**, e49421 (2012).
125. S. Sun *et al.*, Structural analyses at pseudo atomic resolution of Chikungunya virus and antibodies show mechanisms of neutralization. *eLife* **2**, e00435 (2013).
126. R. Adaixo, C. A. Harley, A. F. Castro-Rodrigues, J. H. Morais-Cabral, Structural Properties of PAS Domains from the KCNH Potassium Channels. *PLOS ONE* **8**, e59265 (2013).

127. L. A. Svensson, E. Thulin, S. Forsén, Proline cis-trans isomers in calbindin D9k observed by X-ray crystallography. *Journal of Molecular Biology* **223**, 601-606 (1992).
128. S.-C. Lee *et al.*, Endocytotic Routes of Cobra Cardiotoxins Depend on Spatial Distribution of Positively Charged and Hydrophobic Domains to Target Distinct Types of Sulfated Glycoconjugates on Cell Surface*. *Journal of Biological Chemistry* **289**, 20170-20181 (2014).
129. M. Marquart, J. Walter, J. Deisenhofer, W. Bode, R. Huber, The geometry of the reactive site and of the peptide groups in trypsin, trypsinogen and its complexes with inhibitors. *Acta Crystallographica Section B* **39**, 480-490 (1983).
130. Y. Wen *et al.*, The bacterial antitoxin HipB establishes a ternary complex with operator DNA and phosphorylated toxin HipA to regulate bacterial persistence. *Nucleic Acids Research* **42**, 10134-10147 (2014).
131. E. Teplitsky *et al.*, High throughput screening using acoustic droplet ejection to combine protein crystals and chemical libraries on crystallization plates at high density. *Journal of Structural Biology* **191**, 49-58 (2015).
132. Y. Watanabe, Y. Tamura, S. Kawano, T. Endo, Structural and mechanistic insights into phospholipid transfer by Ups1–Mdm35 in mitochondria. *Nature Communications* **6**, 7922 (2015).
133. L. Lebioda, B. Stec, J. M. Brewer, E. Tykarska, Inhibition of enolase: the crystal structures of enolase-calcium(2+)-2-phosphoglycerate and enolase-zinc(2+)-phosphoglycolate complexes at 2.2-.ANG. resolution. *Biochemistry* **30**, 2823-2827 (1991).
134. M. Bommer, A.-N. Bondar, A. Zouni, H. Dobbek, H. Dau, Crystallographic and Computational Analysis of the Barrel Part of the PsbO Protein of Photosystem II: Carboxylate–Water Clusters as Putative Proton Transfer Relays and Structural Switches. *Biochemistry* **55**, 4626-4635 (2016).
135. J.-H. Na, S.-S. Cha, Structural basis for the extended substrate spectrum of AmpC BER and structure-guided discovery of the inhibition activity of citrate against the class C [beta]-lactamases AmpC BER and CMY-10. *Acta Crystallographica Section D* **72**, 976-985 (2016).
136. M. Bečková *et al.*, Structure of Psb29/Thf1 and its association with the FtsH protease complex involved in photosystem II repair in cyanobacteria. *Philosophical Transactions of the Royal Society B: Biological Sciences* **372**, 20160394 (2017).
137. M. Bayrhuber *et al.*, Nuclear Magnetic Resonance Solution Structure and Functional Behavior of the Human Proton Channel. *Biochemistry* **58**, 4017-4027 (2019).
138. E. F. Pai *et al.*, Refined crystal structure of the triphosphate conformation of H-ras p21 at 1.35 A resolution: implications for the mechanism of GTP hydrolysis. *The EMBO Journal* **9**, 2351-2359 (1990).
139. A. Wlodawer, N. Borkakoti, D. S. Moss, B. Howlin, Comparison of two independently refined models of ribonuclease-A. *Acta Crystallographica Section B* **42**, 379-387 (1986).
140. 5yl6. https://www.rcsb.org/structure/5YL6.
141. S.-H. Park *et al.*, Crystal structure and enzymatic properties of chalcone isomerase from the Antarctic vascular plant Deschampsia antarctica Desv. *PLOS ONE* **13**, e0192415 (2018).
142. 5yxd. https://www.rcsb.org/structure/5YXD.
143. 5z1y. https://www.rcsb.org/structure/5Z1Y.

144. 5z26. https://www.rcsb.org/structure/5Z26.
145. C. W. Lee *et al.*, Crystal structure of dihydrodipicolinate reductase (PaDHDPR) from Paenisporosarcina sp. TG-14: structural basis for NADPH preference as a cofactor. *Scientific Reports* **8**, 7936 (2018).
146. 5z2o. https://www.rcsb.org/structure/5Z2O.
147. Y. Choi *et al.*, Structural Insights into the FtsQ/FtsB/FtsL Complex, a Key Component of the Divisome. *Scientific Reports* **8**, 18061 (2018).
148. S. A. Mohid *et al.*, Application of tungsten disulfide quantum dot-conjugated antimicrobial peptides in bio-imaging and antimicrobial therapy. *Colloids and Surfaces B: Biointerfaces* **176**, 360-370 (2019).
149. S. Zong *et al.*, Structure of the intact 14-subunit human cytochrome c oxidase. *Cell Research* **28**, 1026-1034 (2018).
150. T. Tanaka *et al.*, High-Resolution Protein 3D Structure Determination in Living Eukaryotic Cells. *Angewandte Chemie International Edition* **58**, 7284-7288 (2019).
151. J. Gartia *et al.*, NMR structure and dynamics of inhibitory repeat domain variant 12, a plant protease inhibitor from Capsicum annuum, and its structural relationship to other plant protease inhibitors. *Journal of Biomolecular Structure and Dynamics* **38**, 1388-1397 (2020).
152. E. T. H. Goh *et al.*, A Small Molecule Targeting the Transmembrane Domain of Death Receptor p75NTR Induces Melanoma Cell Death and Reduces Tumor Growth. *Cell Chemical Biology* **25**, 1485-1494.e1485 (2018).
153. 5zrn. https://www.rcsb.org/structure/5ZRN.
154. 5zsy. https://www.rcsb.org/structure/5ZSY.
155. 5zsz. https://www.rcsb.org/structure/5ZSZ.
156. F. Shan *et al.*, A telomerase subunit homolog La protein from Trypanosoma brucei plays an essential role in ribosomal biogenesis. *The FEBS Journal* **286**, 3129-3147 (2019).
157. 5zv6. https://www.rcsb.org/structure/5ZV6.
158. R. Dwivedi, P. Aggarwal, N. S. Bhavesh, K. J. Kaur, Design of therapeutically improved analogue of the antimicrobial peptide, indolicidin, using a glycosylation strategy. *Amino Acids* **51**, 1443-1460 (2019).
159. K. G. Varnava *et al.*, Design, Synthesis, Antibacterial Potential, and Structural Characterization of N-Acylated Derivatives of the Human Autophagy 16 Polypeptide. *Bioconjugate Chemistry* **30**, 1998-2010 (2019).
160. L. Ma *et al.*, A Glimpse of the Peptide Profile Presentation by Xenopus laevis MHC Class I: Crystal Structure of pXela-UAA Reveals a Distinct Peptide-Binding Groove. *The Journal of Immunology* **204**, 147-158 (2020).
161. A. Suka *et al.*, Stability of cytochromes c′ from psychrophilic and piezophilic Shewanella species: implications for complex multiple adaptation to low temperature and high hydrostatic pressure. *Extremophiles* **23**, 239-248 (2019).
162. 6a3z. https://www.rcsb.org/structure/6A3Z.
163. 6a4c. https://www.rcsb.org/structure/6A4C.
164. S. E. Banijamali, M. Amininasab, D. Zaeifi, Structural characterization of PPTI, a kunitz-type protein from the venom of Pseudocerastes persicus. *PLOS ONE* **14**, e0214657 (2019).
165. 6a5j. https://www.rcsb.org/structure/6A5J.
166. 6a8y. https://www.rcsb.org/structure/6A8Y.

167. 6aab. https://www.rcsb.org/structure/6AAB.
168. 6acl. https://www.rcsb.org/structure/6ACL.
169. N. Takekawa *et al.*, Structure of <i>Vibrio</i> FliL, a New Stomatin-like Protein That Assists the Bacterial Flagellar Motor Function. *mBio* **10**, 10.1128/mbio.00292-00219 (2019).
170. 6ak7. https://www.rcsb.org/structure/6AK7.
171. R. Mahling *et al.*, NaV1.2 EFL domain allosterically enhances Ca2+ binding to sites I and II of WT and pathogenic calmodulin mutants bound to the channel CTD. *Structure* **29**, 1339-1356.e1337 (2021).
172. J.-A. Jentsch *et al.*, Structural basis of sterol binding and transport by a yeast StARkin domain. *Journal of Biological Chemistry* **293**, 5522-5531 (2018).
173. 6c0a. https://www.rcsb.org/structure/6C0A.
174. 6c37. https://www.rcsb.org/structure/6C37.
175. S. L. Kall, K. Whitlatch, T. E. Smithgall, A. Lavie, Molecular basis for the interaction between human choline kinase alpha and the SH3 domain of the c-Src tyrosine kinase. *Scientific Reports* **9**, 17121 (2019).
176. L. Lan *et al.*, Crystal and solution structures of human oncoprotein Musashi-2 N-terminal RNA recognition motif 1. *Proteins: Structure, Function, and Bioinformatics* **88**, 573-583 (2020).
177. K. Ellis-Guardiola *et al.*, Crystal Structure and Conformational Dynamics of Pyrococcus furiosus Prolyl Oligopeptidase. *Biochemistry* **58**, 1616-1626 (2019).
178. 6cfa. https://www.rcsb.org/structure/6CFA.
179. O. K. Shrestha *et al.*, Structure and evolution of the 4-helix bundle domain of Zuotin, a J-domain protein co-chaperone of Hsp70. *PLOS ONE* **14**, e0217098 (2019).
180. J. R. Burke *et al.*, Bifunctional Substrate Activation via an Arginine Residue Drives Catalysis in Chalcone Isomerases. *ACS Catalysis* **9**, 8388-8396 (2019).
181. D. Flores-Solis *et al.*, Solution structure of the inhibitor of cysteine proteases 1 from Entamoeba histolytica reveals a possible auto regulatory mechanism. *Biochimica et Biophysica Acta (BBA) - Proteins and Proteomics* **1868**, 140512 (2020).
182. P. Sunanda *et al.*, Identification, chemical synthesis, structure, and function of a new KV1 channel blocking peptide from Oulactis sp. *Peptide Science* **110**, e24073 (2018).
183. 6cqg. https://www.rcsb.org/structure/6CQG.
184. M. H. Cardoso *et al.*, An N-capping asparagine–lysine–proline (NKP) motif contributes to a hybrid flexible/stable multifunctional peptide scaffold. *Chemical Science* **13**, 9410-9424 (2022).
185. 6ctg. https://www.rcsb.org/structure/6CTG.
186. 6cui. https://www.rcsb.org/structure/6CUI.
187. 6d2h. https://www.rcsb.org/structure/6D2H.
188. G. M. S. Pinheiro, G. C. Amorim, A. Iqbal, F. C. L. Almeida, C. H. I. Ramos, Solution NMR investigation on the structure and function of the isolated J-domain from Sis1: Evidence of transient inter-domain interactions in the full-length protein. *Archives of Biochemistry and Biophysics* **669**, 71-79 (2019).
189. M. Shrestha *et al.*, Structure of the sensory domain of McpX from Sinorhizobium meliloti, the first known bacterial chemotactic sensor for quaternary ammonium compounds. *Biochemical Journal* **475**, 3949-3962 (2018).

190. M. Mayorga-Flores *et al.*, Novel Blocker of Onco SK3 Channels Derived from Scorpion Toxin Tamapin and Active against Migration of Cancer Cells. *ACS Medicinal Chemistry Letters* **11**, 1627-1633 (2020).
191. A. McPherson, S. B. Larson, The X-ray crystal structure of human endothelin 1, a polypeptide hormone regulator of blood pressure. *Acta Crystallographica Section F* **75**, 47-53 (2019).
192. G. A. Letourneau, T. N. Wright, Structural Insights on the Obscurin-Binding Domains in Titin. *Protein & Peptide Letters* **25**, 973-979 (2018).
193. I. V. Peshenko *et al.*, Retinal degeneration 3 (RD3) protein, a retinal guanylyl cyclase regulator, forms a monomeric and elongated four-helix bundle. *Journal of Biological Chemistry* **294**, 2318-2328 (2019).
194. 6dri. https://www.rcsb.org/structure/6DRI.
195. B. K. Miller *et al.*, Mycobacterium tuberculosis SatS is a chaperone for the SecA2 protein export pathway. *eLife* **8**, e40063 (2019).
196. 6ds8. https://www.rcsb.org/structure/6DS8.
197. L. S. Ramirez, J. Pande, A. Shekhtman, Helical Structure of Recombinant Melittin. *The Journal of Physical Chemistry B* **123**, 356-368 (2019).
198. 6dul. https://www.rcsb.org/structure/6DUL.
199. 6duu. https://www.rcsb.org/structure/6DUU.
200. Y.-T. Sun, M. D. Shortridge, G. Varani, A Small Cyclic β-Hairpin Peptide Mimics the Rbfox2 RNA Recognition Motif and Binds to the Precursor miRNA 20b. *ChemBioChem* **20**, 931-939 (2019).
201. M. Dastpeyman *et al.*, Structural Variants of a Liver Fluke Derived Granulin Peptide Potently Stimulate Wound Healing. *Journal of Medicinal Chemistry* **61**, 8746-8753 (2018).
202. A. Mitra, Y.-H. Ko, G. Cingolani, M. Niederweis, Heme and hemoglobin utilization by Mycobacterium tuberculosis. *Nature Communications* **10**, 4260 (2019).
203. G. Cingolani *et al.*, Structural basis for the homotypic fusion of chlamydial inclusions by the SNARE-like protein IncA. *Nature Communications* **10**, 2747 (2019).
204. 6e98. https://www.rcsb.org/structure/6E98.
205. 6e9m. https://www.rcsb.org/structure/6E9M.
206. D. L. Moss, H.-W. Park, R. R. Mettu, S. J. Landry, Deimmunizing substitutions in Pseudomonas exotoxin domain III perturb antigen processing without eliminating T-cell epitopes. *Journal of Biological Chemistry* **294**, 4667-4681 (2019).
207. F. Wang *et al.*, Structure of Microbial Nanowires Reveals Stacked Hemes that Transport Electrons over Micrometers. *Cell* **177**, 361-369.e310 (2019).
208. N. Darvill *et al.*, Structural Basis of Phosphatidic Acid Sensing by APH in Apicomplexan Parasites. *Structure* **26**, 1059-1071.e1056 (2018).
209. 6f98. https://www.rcsb.org/structure/6F98.
210. 6f9a. https://www.rcsb.org/structure/6F9A.
211. A. Sendoel *et al.*, MINA-1 and WAGO-4 are part of regulatory network coordinating germ cell death and RNAi in C. elegans. *Cell Death & Differentiation* **26**, 2157-2178 (2019).
212. H. A. Heikkinen, S. M. Backlund, H. Iwaï, NMR Structure Determinations of Small Proteins Using only One Fractionally 20% 13C- and Uniformly 100% 15N-Labeled Sample. *Molecules* **26**, 747 (2021).

213. C. S. F. C. Popov *et al.*, Host-defense peptides AC12, DK16 and RC11 with immunomodulatory activity isolated from Hypsiboas raniceps skin secretion. *Peptides* **113**, 11-21 (2019).
214. P. F. Varela *et al.*, Biophysical and structural characterization of a zinc-responsive repressor of the MarR superfamily. *PLOS ONE* **14**, e0210123 (2019).
215. 6fmg. https://www.rcsb.org/structure/6FMG.
216. 6fsl. https://www.rcsb.org/structure/6FSL.
217. M. Grimaldi *et al.*, NMR Structure of the FIV gp36 C-terminal Heptad Repeat and Membrane-Proximal External Region. *International Journal of Molecular Sciences* **21**, 2037 (2020).
218. A. J. F. Egan *et al.*, Induced conformational changes activate the peptidoglycan synthase PBP1B. *Molecular Microbiology* **110**, 335-356 (2018).
219. 6g4a. https://www.rcsb.org/structure/6G4A.
220. P. B. Timmons, D. O'Flynn, J. M. Conlon, C. M. Hewage, Structural and positional studies of the antimicrobial peptide brevinin-1BYa in membrane-mimetic environments. *Journal of Peptide Science* **25**, e3208 (2019).
221. K. V. Rajasekar *et al.*, Structure of SPH (self-incompatibility protein homologue) proteins: a widespread family of small, highly stable, secreted proteins. *Biochemical Journal* **476**, 809-826 (2019).
222. I. Vasiliauskaité-Brooks *et al.*, Structure of a human intramembrane ceramidase explains enzymatic dysfunction found in leukodystrophy. *Nature Communications* **9**, 5437 (2018).
223. C. Zoukimian *et al.*, Synthesis by native chemical ligation and characterization of the scorpion toxin AmmTx3. *Bioorganic & Medicinal Chemistry* **27**, 247-253 (2019).
224. D. N. Korkut *et al.*, Structural insights into the AapA1 toxin of Helicobacter pylori. *Biochimica et Biophysica Acta (BBA) - General Subjects* **1864**, 129423 (2020).
225. B. Bardiaux *et al.*, Structure and Assembly of the Enterohemorrhagic Escherichia coli Type 4 Pilus. *Structure* **27**, 1082-1093.e1085 (2019).
226. B. Ekblad, P. E. Kristiansen, NMR structures and mutational analysis of the two peptides constituting the bacteriocin plantaricin S. *Scientific Reports* **9**, 2333 (2019).
227. A. Andreou *et al.*, The putative polysaccharide deacetylase Ba0331: cloning, expression, crystallization and structure determination. *Acta Crystallographica Section F* **75**, 312-320 (2019).
228. L. D. Nielsen, C. P. Pedersen, S. Erlendsson, K. Teilum, The Capsid Domain of Arc Changes Its Oligomerization Propensity through Direct Interaction with the NMDA Receptor. *Structure* **27**, 1071-1081.e1075 (2019).
229. J. Lisboa *et al.*, The C-terminal domain of HpDprA is a DNA-binding winged helix domain that does not bind double-stranded DNA. *The FEBS Journal* **286**, 1941-1958 (2019).
230. L. Moretto, R. Heylen, N. Holroyd, S. Vance, R. W. Broadhurst, Modular type I polyketide synthase acyl carrier protein domains share a common N-terminally extended fold. *Scientific Reports* **9**, 2325 (2019).
231. G. Ferré *et al.*, Structure and dynamics of G protein-coupled receptor–bound ghrelin reveal the critical role of the octanoyl chain. *Proceedings of the National Academy of Sciences* **116**, 17525-17530 (2019).
232. 6h7s. https://www.rcsb.org/structure/6H7S.

233. J. Huber *et al.*, An atypical LIR motif within UBA5 (ubiquitin like modifier activating enzyme 5) interacts with GABARAP proteins and mediates membrane localization of UBA5. *Autophagy* **16**, 256-270 (2020).
234. K. Gruber, V. Csitkovits, A. Łyskowski, C. Kratky, B. Kräutler, Structure-Based Demystification of Radical Catalysis by a Coenzyme B12 Dependent Enzyme—Crystallographic Study of Glutamate Mutase with Cofactor Homologues. *Angewandte Chemie International Edition* **61**, e202208295 (2022).
235. H. C. Rice *et al.*, Secreted amyloid-β precursor protein functions as a GABA_BR1a ligand to modulate synaptic transmission. *Science* **363**, eaao4827 (2019).
236. S. A. Hughes *et al.*, Ambidextrous helical nanotubes from self-assembly of designed helical hairpin motifs. *Proceedings of the National Academy of Sciences* **116**, 14456-14464 (2019).
237. C. Perez, A. R. Mehdipour, G. Hummer, K. P. Locher, Structure of Outward-Facing PglK and Molecular Dynamics of Lipid-Linked Oligosaccharide Recognition and Translocation. *Structure* **27**, 669-678.e665 (2019).
238. M. Dujardin *et al.*, Cyclophilin A allows the allosteric regulation of a structural motif in the disordered domain 2 of NS5A and thereby fine-tunes HCV RNA replication. *Journal of Biological Chemistry* **294**, 13171-13185 (2019).
239. H. Nassour *et al.*, Lipidated peptides derived from intracellular loops 2 and 3 of the urotensin II receptor act as biased allosteric ligands. *Journal of Biological Chemistry* **297**, 101057 (2021).
240. J.-L. Berry *et al.*, Global biochemical and structural analysis of the type IV pilus from the Gram-positive bacterium Streptococcus sanguinis. *Journal of Biological Chemistry* **294**, 6796-6808 (2019).
241. X. Miliara *et al.*, Structural determinants of lipid specificity within Ups/PRELI lipid transfer proteins. *Nature Communications* **10**, 1130 (2019).
242. I. Cruz-Gallardo *et al.*, LARP4A recognizes polyA RNA via a novel binding mechanism mediated by disordered regions and involving the PAM2w motif, revealing interplay between PABP, LARP4A and mRNA. *Nucleic Acids Research* **47**, 4272-4291 (2019).
243. J. S. Scott *et al.*, Tricyclic Indazoles—A Novel Class of Selective Estrogen Receptor Degrader Antagonists. *Journal of Medicinal Chemistry* **62**, 1593-1608 (2019).
244. A. S. Paramonov *et al.*, Structural Diversity and Dynamics of Human Three-Finger Proteins Acting on Nicotinic Acetylcholine Receptors. *International Journal of Molecular Sciences* **21**, 7280 (2020).
245. C. Kamischke *et al.*, The Acinetobacter baumannii Mla system and glycerophospholipid transport to the outer membrane. *eLife* **8**, e40171 (2019).
246. S. K. Yadav, Archana, R. Singh, P. K. Singh, P. G. Vasudev, Insecticidal fern protein Tma12 is possibly a lytic polysaccharide monooxygenase. *Planta* **249**, 1987-1996 (2019).
247. 6iha. https://www.rcsb.org/structure/6IHA.
248. Y. K. Toh *et al.*, Effect of the additional cysteine 503 of vancomycin-resistant Enterococcus faecalis (V583) alkylhydroperoxide reductase subunit F (AhpF) and the mechanism of AhpF and subunit C assembling. *Free Radical Biology and Medicine* **138**, 10-22 (2019).
249. 6ilb. https://www.rcsb.org/structure/6ILB.

250. X. Zhen *et al.*, Structural basis of AimP signaling molecule recognition by AimR in Spbeta group of bacteriophages. *Protein & Cell* **10**, 131-136 (2018).
251. P. Lin, H. Yao, J. Zha, Y. Zhao, C. Wu, Ordered and Isomerically Stable Bicyclic Peptide Scaffolds Constrained through Cystine Bridges and Proline Turns. *ChemBioChem* **20**, 1514-1518 (2019).
252. K. Miyamoto, Y. Fujiwara, K. Saito, Zinc finger domain of the human DTX protein adopts a unique RING fold. *Protein Science* **28**, 1151-1156 (2019).
253. X. Wang *et al.*, Structural interaction between DISC1 and ATF4 underlying transcriptional and synaptic dysregulation in an iPSC model of mental disorders. *Molecular Psychiatry* **26**, 1346-1360 (2021).
254. 6ixx. https://www.rcsb.org/structure/6IXX.
255. 6iy4. https://www.rcsb.org/structure/6IY4.
256. 6izg. https://www.rcsb.org/structure/6IZG.
257. F. Koh *et al.*, The structure of a 15-stranded actin-like filament from Clostridium botulinum. *Nature Communications* **10**, 2856 (2019).
258. Y. Wang *et al.*, The meiotic TERB1-TERB2-MAJIN complex tethers telomeres to the nuclear envelope. *Nature Communications* **10**, 564 (2019).
259. R. Nordström *et al.*, Microgels as carriers of antimicrobial peptides – Effects of peptide PEGylation. *Colloids and Surfaces A: Physicochemical and Engineering Aspects* **565**, 8-15 (2019).
260. D. Lu *et al.*, Peptide presentation by bat MHC class I provides new insight into the antiviral immunity of bats. *PLOS Biology* **17**, e3000436 (2019).
261. X. Yao, Y.-J. Liu, Q. Cui, Y. Feng, Solution structure of a unicellular microalgae-derived translationally controlled tumor protein revealed both conserved features and structural diversity. *Archives of Biochemistry and Biophysics* **665**, 23-29 (2019).
262. 6j9p. https://www.rcsb.org/structure/6J9P.
263. S. Wang *et al.*, Crystal structures of Magnaporthe oryzae trehalose-6-phosphate synthase (MoTps1) suggest a model for catalytic process of Tps1. *Biochemical Journal* **476**, 3227-3240 (2019).
264. R. Zhang *et al.*, Divergent engagements between adeno-associated viruses with their cellular receptor AAVR. *Nature Communications* **10**, 3760 (2019).
265. 6ji7. https://www.rcsb.org/structure/6JI7.
266. D. Liu *et al.*, Structure of TBC1D23 N-terminus reveals a novel role for rhodanese domain. *PLOS Biology* **18**, e3000746 (2020).
267. K. Min *et al.*, Peptidoglycan reshaping by a noncanonical peptidase for helical cell shape in Campylobacter jejuni. *Nature Communications* **11**, 458 (2020).
268. 6jnp. https://www.rcsb.org/structure/6JNP.
269. Z. Wang *et al.*, Expression and characterization of an extremely thermophilic 1,4-α-glucan branching enzyme from Rhodothermus obamensis STB05. *Protein Expression and Purification* **164**, 105478 (2019).
270. S. Ji *et al.*, The molecular basis of endolytic activity of a multidomain alginate lyase from Defluviitalea phaphyphila, a representative of a new lyase family, PL39. *Journal of Biological Chemistry* **294**, 18077-18091 (2019).
271. S. Kim, M.-S. Kim, S. Jo, D. H. Shin, GTP Preference of d-Glycero-α-d-manno-Heptose-1-Phosphate Guanylyltransferase from Yersinia pseudotuberculosis. *International Journal of Molecular Sciences* **21**, 280 (2020).

272. S. Kim *et al.*, The structural basis of the low catalytic activities of the two minor β-carbonic anhydrases of the filamentous fungus Aspergillus fumigatus. *Journal of Structural Biology* **208**, 61-68 (2019).
273. J.-Y. Tsai, Y.-H. Yeh, L.-D. Lin, Y.-J. Sun, C.-D. Hsiao, Crystal structure of the flagellin protein FlaG from Helicobacter pylori. *Journal of the Chinese Chemical Society* **66**, 1178-1185 (2019).
274. P. Bai *et al.*, Rational discovery of a cancer neoepitope harboring the KRAS G12D driver mutation. *Science China Life Sciences* **64**, 2144-2152 (2021).
275. X. Li, L. Li, Y. Chang, W. Ning, X. Liu, Structural and functional study of FK domain of Fstl1. *Protein Science* **28**, 1819-1829 (2019).
276. S. Banerjee *et al.*, Inhibition of dual-specificity tyrosine phosphorylation-regulated kinase 2 perturbs 26S proteasome-addicted neoplastic progression. *Proceedings of the National Academy of Sciences* **116**, 24881-24891 (2019).
277. H. Ma *et al.*, Structural comparison of CD163 SRCR5 from different species sheds some light on its involvement in porcine reproductive and respiratory syndrome virus-2 infection in vitro. *Veterinary Research* **52**, 97 (2021).
278. 6k2f. https://www.rcsb.org/structure/6K2F.
279. M.-S. Lee *et al.*, Solution structure of MUL1-RING domain and its interaction with p53 transactivation domain. *Biochemical and Biophysical Research Communications* **516**, 533-539 (2019).
280. Z. Wang *et al.*, Development of chimeric peptides to facilitate the neutralisation of lipopolysaccharides during bactericidal targeting of multidrug-resistant Escherichia coli. *Communications Biology* **3**, 41 (2020).
281. 6k51. https://www.rcsb.org/structure/6K51.
282. V. Tripathi, K. S. Chatterjee, R. Das, Casein kinase-2–mediated phosphorylation increases the SUMO-dependent activity of the cytomegalovirus transactivator IE2. *Journal of Biological Chemistry* **294**, 14546-14561 (2019).
283. W. Xue *et al.*, Domain interactions reveal auto-inhibition of the deubiquitinating enzyme USP19 and its activation by HSP90 in the modulation of huntingtin aggregation. *Biochemical Journal* **477**, 4295-4312 (2020).
284. S. Bala *et al.*, Crystal contact-free conformation of an intrinsically flexible loop in protein crystal: Tim21 as the case study. *Biochimica et Biophysica Acta (BBA) - General Subjects* **1864**, 129418 (2020).
285. N. Shibayama, A. Sato-Tomita, M. Ohki, K. Ichiyanagi, S.-Y. Park, Direct observation of ligand migration within human hemoglobin at work. *Proceedings of the National Academy of Sciences* **117**, 4741-4748 (2020).
286. H. Ilyas, J. Kim, D. Lee, M. Malmsten, A. Bhunia, Structural insights into the combinatorial effects of antimicrobial peptides reveal a role of aromatic–aromatic interactions in antibacterial synergism. *Journal of Biological Chemistry* **294**, 14615-14633 (2019).
287. F. Dong *et al.*, Alginate Lyase Aly36B is a New Bacterial Member of the Polysaccharide Lyase Family 36 and Catalyzes by a Novel Mechanism With Lysine as Both the Catalytic Base and Catalytic Acid. *Journal of Molecular Biology* **431**, 4897-4909 (2019).
288. Y. Yang, Y. Ding, C. Zhou, Y. Wen, N. Zhang, Structural and functional studies of USP20 ZnF-UBP domain by NMR. *Protein Science* **28**, 1606-1619 (2019).

289. K.-N. Feng *et al.*, A Hydrolase-Catalyzed Cyclization Forms the Fused Bicyclic β-Lactone in Vibralactone. *Angewandte Chemie International Edition* **59**, 7209-7213 (2020).
290. 6kga. https://www.rcsb.org/structure/6KGA.
291. L. Kim, D. H. Kwon, J. Heo, M. R. Park, H. K. Song, Use of the LC3B-fusion technique for biochemical and structural studies of proteins involved in the N-degron pathway. *Journal of Biological Chemistry* **295**, 2590-2600 (2020).
292. 6khu. https://www.rcsb.org/structure/6KHU.
293. N. Nishida *et al.*, Structural basis for two-way communication between dynein and microtubules. *Nature Communications* **11**, 1038 (2020).
294. Y. El Mannai, R. Deto, M. Kuroki, R. Suzuki, E. Suzuki, Cyanobacterial branching enzymes bind to α-glucan via surface binding sites. *Archives of Biochemistry and Biophysics* **702**, 108821 (2021).
295. A. Kam *et al.*, Roseltide rT7 is a disulfide-rich, anionic, and cell-penetrating peptide that inhibits proteasomal degradation. *Journal of Biological Chemistry* **294**, 19604-19615 (2019).
296. M. K. Mohan *et al.*, Structure and allosteric activity of a single-disulfide conopeptide from Conus zonatus at human α3β4 and α7 nicotinic acetylcholine receptors. *Journal of Biological Chemistry* **295**, 7096-7112 (2020).
297. K. Wang *et al.*, Structural Mechanism for GSDMD Targeting by Autoprocessed Caspases in Pyroptosis. *Cell* **180**, 941-955.e920 (2020).
298. Y. Ye, M. Chen, K. Kato, M. Yao, The pH-dependent conformational change of eukaryotic translation initiation factor 5: Insights into partner-binding manner. *Biochemical and Biophysical Research Communications* **519**, 186-191 (2019).
299. 6kq1. https://www.rcsb.org/structure/6KQ1.
300. 6kra. https://www.rcsb.org/structure/6KRA.
301. S. Chatterjee, S. Nath, U. Sen, High resolution structure of Vibrio cholerae acylphosphatase (VcAcP) cage: Identification of drugs, location of its binding site and engineering to facilitate cage formation. *Biochemical and Biophysical Research Communications* **523**, 348-353 (2020).
302. C. M. Kim *et al.*, Crystal Structure of the Active Site Mutant Form of Soluble Fumarate Reductase, Osm1. *Crystals* **9**, 504 (2019).
303. G. T. Nguyen *et al.*, Crystal Structure of NADPH-Dependent Methylglyoxal Reductase Gre2 from Candida Albicans. *Crystals* **9**, 471 (2019).
304. Y. Niu *et al.*, A Type I-F Anti-CRISPR Protein Inhibits the CRISPR-Cas Surveillance Complex by ADP-Ribosylation. *Molecular Cell* **80**, 512-524.e515 (2020).
305. C. Liu, J. Ma, J. Wang, H. Wang, L. Zhang, Cryo-EM Structure of a Bacterial Lipid Transporter YebT. *Journal of Molecular Biology* **432**, 1008-1019 (2020).
306. B. Zhou *et al.*, Structural and functional insights into a novel two-component endolysin encoded by a single gene in Enterococcus faecalis phage. *PLOS Pathogens* **16**, e1008394 (2020).
307. 6l2h. https://www.rcsb.org/structure/6L2H.
308. K. Zhao *et al.*, Parkinson's disease associated mutation E46K of α-synuclein triggers the formation of a distinct fibril structure. *Nature Communications* **11**, 2643 (2020).
309. Z. Wang *et al.*, A Bacteriophage DNA Mimic Protein Employs a Non-specific Strategy to Inhibit the Bacterial RNA Polymerase. *Frontiers in Microbiology* **12**, (2021).

310. 6l7n. https://www.rcsb.org/structure/6L7N.
311. H. Noh, J.-H. Jeon, Y.-G. Kim, B.-H. Oh, Crystal structure of PYCH_01220 from Pyrococcus yayanosii potentially involved in binding nucleic acid. *Proteins: Structure, Function, and Bioinformatics* **89**, 468-472 (2021).
312. W. Gong, Q. Liang, Y. Tong, S. Perrett, Y. Feng, Structural Insight into Chromatin Recognition by Multiple Domains of the Tumor Suppressor RBBP1. *Journal of Molecular Biology* **433**, 167224 (2021).
313. M. Wen *et al.*, PD-L1 degradation is regulated by electrostatic membrane association of its cytoplasmic domain. *Nature Communications* **12**, 5106 (2021).
314. F. Lv *et al.*, An amphipathic Bax core dimer forms part of the apoptotic pore wall in the mitochondrial ␣ membrane. *The EMBO Journal* **40**, e106438 (2021).
315. 6l95. https://www.rcsb.org/structure/6L95.
316. X. Zhang *et al.*, 5-IP7 is a GPCR messenger mediating neural control of synaptotagmin-dependent insulin exocytosis and glucose homeostasis. *Nature Metabolism* **3**, 1400-1414 (2021).
317. N. Luan *et al.*, Identification and Characterization of ShSPI, a Kazal-Type Elastase Inhibitor from the Venom of Scolopendra Hainanum. *Toxins* **11**, 708 (2019).
318. X. Wei, S. Wang, S. Wang, X. Xie, N. Zhang, Structure and Peptidomes of Swine MHC Class I with Long Peptides Reveal the Cross-Species Characteristics of the Novel N-Terminal Extension Presentation Mode. *The Journal of Immunology* **208**, 480-491 (2022).
319. S. Y. An *et al.*, Intrinsic disorder is essential for Cas9 inhibition of anti-CRISPR AcrIIA5. *Nucleic Acids Research* **48**, 7584-7594 (2020).
320. M. Wang *et al.*, Interface switch mediates signal transmission in a two-component system. *Proceedings of the National Academy of Sciences* **117**, 30433-30440 (2020).
321. R. Koga *et al.*, Robust folding of a de novo designed ideal protein even with most of the core mutated to valine. *Proceedings of the National Academy of Sciences* **117**, 31149-31156 (2020).
322. 6lr2. https://www.rcsb.org/structure/6LR2.
323. B. Stielow *et al.*, The SAM domain-containing protein 1 (SAMD1) acts as a repressive chromatin regulator at unmethylated CpG islands. *Science Advances* **7**, eabf2229 (2021).
324. 6lvh. https://www.rcsb.org/structure/6LVH.
325. 6lwz. https://www.rcsb.org/structure/6LWZ.
326. K. Dixit *et al.*, Aromatic Interactions Drive the Coupled Folding and Binding of the Intrinsically Disordered Sesbania mosaic Virus VPg Protein. *Biochemistry* **59**, 4663-4680 (2020).
327. 6M0Y. https://www.rcsb.org/structure/6M0Y.
328. 6m3a. https://www.rcsb.org/structure/6M3A.
329. Y. Xie *et al.*, Orchestrated actin nucleation by the Candida albicans polarisome complex enables filamentous growth. *Journal of Biological Chemistry* **295**, 14840-14854 (2020).
330. 6M4Z. https://www.rcsb.org/structure/6M4Z.
331. R. Uehara *et al.*, Crystal structure of a GH1 β-glucosidase from Hamamotoa singularis. *Protein Science* **29**, 2000-2008 (2020).
332. H. Zhao *et al.*, A broad-spectrum virus- and host-targeting peptide against respiratory viruses including influenza virus and SARS-CoV-2. *Nature Communications* **11**, 4252 (2020).

333. 6m5c. https://www.rcsb.org/structure/6M5C.
334. L. Zhu *et al.*, Dynamic folding modulation generates FGF21 variant against diabetes. *EMBO reports* **22**, e51352 (2021).
335. C. Xu *et al.*, Computational design of transmembrane pores. *Nature* **585**, 129-134 (2020).
336. G. D. Brand *et al.*, Intragenic antimicrobial peptides (IAPs) from human proteins with potent antimicrobial and anti-inflammatory activity. *PLOS ONE* **14**, e0220656 (2019).
337. P. M. Wojnarowicz *et al.*, A Small-Molecule Pan-Id Antagonist Inhibits Pathologic Ocular Neovascularization. *Cell Reports* **29**, 62-75.e67 (2019).
338. W. Dai *et al.*, Structure-Based Design of N-(5-Phenylthiazol-2-yl)acrylamides as Novel and Potent Glutathione S-Transferase Omega 1 Inhibitors. *Journal of Medicinal Chemistry* **62**, 3068-3087 (2019).
339. 6mi9. https://www.rcsb.org/structure/6MI9.
340. A. Rodriguez *et al.*, Antimicrobial activity and structure of a consensus human β-defensin and its comparison to a novel putative hBD10. *Proteins: Structure, Function, and Bioinformatics* **88**, 175-186 (2020).
341. Y. Gomez-Llorente *et al.*, Structural basis for active single and double ring complexes in human mitochondrial Hsp60-Hsp10 chaperonin. *Nature Communications* **11**, 1916 (2020).
342. B. Koepnick *et al.*, De novo protein design by citizen scientists. *Nature* **570**, 390-394 (2019).
343. X. Chen *et al.*, Structure of hRpn10 Bound to UBQLN2 UBL Illustrates Basis for Complementarity between Shuttle Factors and Substrates at the Proteasome. *Journal of Molecular Biology* **431**, 939-955 (2019).
344. J. E. Paczkowski *et al.*, An Autoinducer Analogue Reveals an Alternative Mode of Ligand Binding for the LasR Quorum-Sensing Receptor. *ACS Chemical Biology* **14**, 378-389 (2019).
345. W. L. Cheung-Lee, M. E. Parry, A. Jaramillo Cartagena, S. A. Darst, A. J. Link, Discovery and structure of the antimicrobial lasso peptide citrocin. *Journal of Biological Chemistry* **294**, 6822-6830 (2019).
346. A. J. Staup *et al.*, Structure of the SARS-Unique Domain C From the Bat Coronavirus HKU4. *Natural Product Communications* **14**, 1934578X19849202 (2019).
347. K. Luna-Ramirez *et al.*, Structural basis of the potency and selectivity of Urotoxin, a potent Kv1 blocker from scorpion venom. *Biochemical Pharmacology* **174**, 113782 (2020).
348. C. J. Hosford, M. C. Adams, Y. Niu, J. S. Chappie, The N-terminal domain of Staphylothermus marinus McrB shares structural homology with PUA-like RNA binding proteins. *Journal of Structural Biology* **211**, 107572 (2020).
349. Q. Cao, D. R. Boyer, M. R. Sawaya, P. Ge, D. S. Eisenberg, Cryo-EM structures of four polymorphic TDP-43 amyloid cores. *Nature Structural & Molecular Biology* **26**, 619-627 (2019).
350. A. Kerviel *et al.*, Atomic structure of the translation regulatory protein NS1 of bluetongue virus. *Nature Microbiology* **4**, 837-845 (2019).
351. F. Wang *et al.*, An extensively glycosylated archaeal pilus survives extreme conditions. *Nature Microbiology* **4**, 1401-1410 (2019).
352. L. Pan *et al.*, Higher-Order Clustering of the Transmembrane Anchor of DR5 Drives Signaling. *Cell* **176**, 1477-1489.e1414 (2019).

353. K. M. Bauer, R. Dicovitsky, M. Pellegrini, O. Zhaxybayeva, M. J. Ragusa, The structure of a highly-conserved picocyanobacterial protein reveals a Tudor domain with an RNA-binding function. *Journal of Biological Chemistry* **294**, 14333-14344 (2019).
354. N. Ambaye, Noncovalent structure of SENP1 in complex with SUMO2. *Acta Crystallographica Section F* **75**, 332-339 (2019).
355. C. Y. Li *et al.*, Amino Acid Scanning at P5′ within the Bowman–Birk Inhibitory Loop Reveals Specificity Trends for Diverse Serine Proteases. *Journal of Medicinal Chemistry* **62**, 3696-3706 (2019).
356. T. A. Chew *et al.*, Structure and mechanism of the cation–chloride cotransporter NKCC1. *Nature* **572**, 488-492 (2019).
357. M. M. Verstraete *et al.*, The heme-sensitive regulator SbnI has a bifunctional role in staphyloferrin B production by Staphylococcus aureus. *Journal of Biological Chemistry* **294**, 11622-11636 (2019).
358. W. Wang *et al.*, The Cap-Snatching SFTSV Endonuclease Domain Is an Antiviral Target. *Cell Reports* **30**, 153-163.e155 (2020).
359. R. O. Frederick *et al.*, Function and solution structure of the Arabidopsis thaliana RALF8 peptide. *Protein Science* **28**, 1115-1126 (2019).
360. H. Cárdenas-Hernández *et al.*, Genome-wide and structural analysis of the Myb-SHAQKYF family in Entamoeba histolytica. *Biochimica et Biophysica Acta (BBA) - Proteins and Proteomics* **1869**, 140601 (2021).
361. H. A. Bunzel *et al.*, Emergence of a Negative Activation Heat Capacity during Evolution of a Designed Enzyme. *Journal of the American Chemical Society* **141**, 11745-11748 (2019).
362. G. B. Gurrola *et al.*, Cn29, a novel orphan peptide found in the venom of the scorpion Centruroides noxius: Structure and function. *Toxicon* **167**, 184-191 (2019).
363. A. Piserchio *et al.*, Solution Structure of the Carboxy-Terminal Tandem Repeat Domain of Eukaryotic Elongation Factor 2 Kinase and Its Role in Substrate Recognition. *Journal of Molecular Biology* **431**, 2700-2717 (2019).
364. Y. Cao *et al.*, ABHD10 is an S-depalmitoylase affecting redox homeostasis through peroxiredoxin-5. *Nature Chemical Biology* **15**, 1232-1240 (2019).
365. 6nzl. *https://www.rcsb.org/structure/6NZL*.
366. 6nzs. *https://www.rcsb.org/structure/6NZS*.
367. J. Zhang *et al.*, An Ancient Peptide Family Buried within Vicilin Precursors. *ACS Chemical Biology* **14**, 979-993 (2019).
368. A. Bahramzadeh, T. Huber, G. Otting, Three-Dimensional Protein Structure Determination Using Pseudocontact Shifts of Backbone Amide Protons Generated by Double-Histidine Co2+-Binding Motifs at Multiple Sites. *Biochemistry* **58**, 3243-3250 (2019).
369. F. R. Fields *et al.*, Synthetic Antimicrobial Peptide Tuning Permits Membrane Disruption and Interpeptide Synergy. *ACS Pharmacology & Translational Science* **3**, 418-424 (2020).
370. C. A. Barnes *et al.*, Remarkable Rigidity of the Single α-Helical Domain of Myosin-VI As Revealed by NMR Spectroscopy. *Journal of the American Chemical Society* **141**, 9004-9017 (2019).
371. 6obk. *https://www.rcsb.org/structure/6OBK*.

372. C.-Y. Chen, W. Lee, P. A. Renhowe, J. Jung, W. R. Montfort, Solution structures of the Shewanella woodyi H-NOX protein in the presence and absence of soluble guanylyl cyclase stimulator IWP-051. *Protein Science* **30**, 448-463 (2021).
373. M. G. Herrera *et al.*, Structure of the Human ACP-ISD11 Heterodimer. *Biochemistry* **58**, 4596-4609 (2019).
374. J. V. Lin King *et al.*, A Cell-Penetrating Scorpion Toxin Enables Mode-Specific Modulation of TRPA1 and Pain. *Cell* **178**, 1362-1374.e1316 (2019).
375. N. Paukovich *et al.*, Streptococcus pneumoniae G5 domains bind different ligands. *Protein Science* **28**, 1797-1805 (2019).
376. Y. Yuan *et al.*, Solution structural model of the complex of the binding regions of human plasminogen with its M-protein receptor from Streptococcus pyogenes. *Journal of Structural Biology* **208**, 18-29 (2019).
377. K. A. Elnahriry *et al.*, Structural and functional characterisation of a novel peptide from the Australian sea anemone Actinia tenebrosa. *Toxicon* **168**, 104-112 (2019).
378. 6otb. *https://www.rcsb.org/structure/6OTB*.
379. 6ovj. *https://www.rcsb.org/structure/6OVJ*.
380. P. L. Campbell, R. L. Duda, J. Nassur, J. F. Conway, A. Huet, Mobile Loops and Electrostatic Interactions Maintain the Flexible Tail Tube of Bacteriophage Lambda. *Journal of Molecular Biology* **432**, 384-395 (2020).
381. S. L. Crespo-Flores, A. Cabezas, S. Hassan, Y. Wei, PEA-15 C-Terminal Tail Allosterically Modulates Death-Effector Domain Conformation and Facilitates Protein–Protein Interactions. *International Journal of Molecular Sciences* **20**, 3335 (2019).
382. M. Ali *et al.*, Trapping of a Putative Intermediate in the Cytochrome c Nitrite Reductase (ccNiR)-Catalyzed Reduction of Nitrite: Implications for the ccNiR Reaction Mechanism. *Journal of the American Chemical Society* **141**, 13358-13371 (2019).
383. F. Comert *et al.*, The host-defense peptide piscidin P1 reorganizes lipid domains in membranes and decreases activation energies in mechanosensitive ion channels. *Journal of Biological Chemistry* **294**, 18557-18570 (2019).
384. D. Tavella, A. Ertekin, H. Schaal, S. P. Ryder, F. Massi, A Disorder-to-Order Transition Mediates RNA Binding of the Caenorhabditis elegans Protein MEX-5. *Biophysical Journal* **118**, 2001-2014 (2020).
385. K. Manne *et al.*, Novel structure of the N-terminal helical domain of BibA, a group B streptococcus immunogenic bacterial adhesin. *Acta Crystallographica Section D* **76**, 759-770 (2020).
386. W. L. Cheung-Lee *et al.*, Discovery of Ubonodin, an Antimicrobial Lasso Peptide Active against Members of the Burkholderia cepacia Complex. *ChemBioChem* **21**, 1335-1340 (2020).
387. A.-H. Jin *et al.*, Conotoxin Φ-MiXXVIIA from the Superfamily G2 Employs a Novel Cysteine Framework that Mimics Granulin and Displays Anti-Apoptotic Activity. *Angewandte Chemie International Edition* **56**, 14973-14976 (2017).
388. Y. Jiang, P. Rossi, C. G. Kalodimos, Structural basis for client recognition and activity of Hsp40 chaperones. *Science* **365**, 1313-1319 (2019).
389. M. Enomoto *et al.*, Coordination of a Single Calcium Ion in the EF-hand Maintains the Off State of the Stromal Interaction Molecule Luminal Domain. *Journal of Molecular Biology* **432**, 367-383 (2020).
390. 6pw8. *https://www.rcsb.org/structure/6PW8*.

391. J. Jin *et al.*, Weaponisation ‘on the fly’: Convergent recruitment of knottin and defensin peptide scaffolds into the venom of predatory assassin flies. *Insect Biochemistry and Molecular Biology* **118**, 103310 (2020).
392. W. L. Cheung-Lee, L. Cao, A. J. Link, Pandonodin: A Proteobacterial Lasso Peptide with an Exceptionally Long C-Terminal Tail. *ACS Chemical Biology* **14**, 2783-2792 (2019).
393. D. Coombes *et al.*, The basis for non-canonical ROK family function in the N-acetylmannosamine kinase from the pathogen Staphylococcus aureus. *Journal of Biological Chemistry* **295**, 3301-3315 (2020).
394. N. Kubatova *et al.*, Solution Structure and Dynamics of the Small Protein HVO_2922 from Haloferax volcanii. *ChemBioChem* **21**, 149-156 (2020).
395. L. D. Nielsen *et al.*, The three-dimensional structure of an H-superfamily conotoxin reveals a granulin fold arising from a common ICK cysteine framework. *Journal of Biological Chemistry* **294**, 8745-8759 (2019).
396. M. Hassler *et al.*, Structural Basis of an Asymmetric Condensin ATPase Cycle. *Molecular Cell* **74**, 1175-1188.e1179 (2019).
397. L. Calvanese *et al.*, Structural insights on P31-43, a gliadin peptide able to promote an innate but not an adaptive response in celiac disease. *Journal of Peptide Science* **25**, e3161 (2019).
398. K. Loth *et al.*, The Ancestral N-Terminal Domain of Big Defensins Drives Bacterially Triggered Assembly into Antimicrobial Nanonets. *mBio* **10**, 10.1128/mbio.01821-01819 (2019).
399. K. S. Usachev *et al.*, Solution structure of the N-terminal domain of the Staphylococcus aureus hibernation promoting factor. *Journal of Biomolecular NMR* **73**, 223-227 (2019).
400. R. D. Righetto, N. Biyani, J. Kowal, M. Chami, H. Stahlberg, Retrieving high-resolution information from disordered 2D crystals by single-particle cryo-EM. *Nature Communications* **10**, 1722 (2019).
401. N. Guyot *et al.*, Structure, function, and evolution of <i>Gga</i>-AvBD11, the archetype of the structural avian-double-β-defensin family. *Proceedings of the National Academy of Sciences* **117**, 337-345 (2020).
402. S. S. Denisov *et al.*, Tick saliva protein Evasin-3 modulates chemotaxis by disrupting CXCL8 interactions with glycosaminoglycans and CXCR2. *Journal of Biological Chemistry* **294**, 12370-12379 (2019).
403. W. Zhang *et al.*, Heparin-induced tau filaments are polymorphic and differ from those in Alzheimer’s and Pick’s diseases. *eLife* **8**, e43584 (2019).
404. A. Beil *et al.*, The Solution Structure and Dynamics of Cd-Metallothionein from Helix pomatia Reveal Optimization for Binding Cd over Zn. *Biochemistry* **58**, 4570-4581 (2019).
405. J. Fridmanis, R. Bobrovs, K. Brangulis, K. Tārs, K. Jaudzems, Structural and Functional Analysis of BBA03, Borrelia burgdorferi Competitive Advantage Promoting Outer Surface Lipoprotein. *Pathogens* **9**, 826 (2020).
406. O. Dobrovolska *et al.*, The Arabidopsis (ASHH2) CW domain binds monomethylated K4 of the histone H3 tail through conformational selection. *The FEBS Journal* **287**, 4458-4480 (2020).
407. R. Molina *et al.*, Structure of Csx1-cOA4 complex reveals the basis of RNA decay in Type III-B CRISPR-Cas. *Nature Communications* **10**, 4302 (2019).

408. J. Began *et al.*, Rhomboid intramembrane protease YqgP licenses bacterial membrane protein quality control as adaptor of FtsH AAA protease. *The EMBO Journal* **39**, e102935 (2020).
409. A. Chaves-Sanjuan *et al.*, Structural determinants for NF-Y subunit organization and NF-Y/DNA association in plants. *The Plant Journal* **105**, 49-61 (2021).
410. J. S. Fenn *et al.*, Structural Basis of Glycerophosphodiester Recognition by the Mycobacterium tuberculosis Substrate-Binding Protein UgpB. *ACS Chemical Biology* **14**, 1879-1887 (2019).
411. K. Jaudzems *et al.*, Targeting Bacterial Sortase A with Covalent Inhibitors: 27 New Starting Points for Structure-Based Hit-to-Lead Optimization. *ACS Infectious Diseases* **6**, 186-194 (2020).
412. A. Ignatiou *et al.*, Structural transitions during the scaffolding-driven assembly of a viral capsid. *Nature Communications* **10**, 4840 (2019).
413. M. Akram *et al.*, A nitric oxide–binding heterodimeric cytochrome c complex from the anammox bacterium Kuenenia stuttgartiensis binds to hydrazine synthase. *Journal of Biological Chemistry* **294**, 16712-16728 (2019).
414. S. W. Manka, C. A. Moores, Pseudo-repeats in doublecortin make distinct mechanistic contributions to microtubule regulation. *EMBO reports* **21**, e51534 (2020).
415. A. Sukova *et al.*, Negative charge of the AC-to-Hly linking segment modulates calcium-dependent membrane activities of Bordetella adenylate cyclase toxin. *Biochimica et Biophysica Acta (BBA) - Biomembranes* **1862**, 183310 (2020).
416. 6rpk. https://www.rcsb.org/structure/6RPK.
417. N. Essawy *et al.*, An Emerin LEM-Domain Mutation Impairs Cell Response to Mechanical Stress. *Cells* **8**, 570 (2019).
418. L. A. Malone *et al.*, Cryo-EM structure of the spinach cytochrome b6 f complex at 3.6 Å resolution. *Nature* **575**, 535-539 (2019).
419. T. Weinert *et al.*, Proton uptake mechanism in bacteriorhodopsin captured by serial synchrotron crystallography. *Science* **365**, 61-65 (2019).
420. D. Pérez-Amigot *et al.*, Towards the competent conformation for catalysis in the ferredoxin-NADP+ reductase from the Brucella ovis pathogen. *Biochimica et Biophysica Acta (BBA) - Bioenergetics* **1860**, 148058 (2019).
421. E. N. Grafskaia *et al.*, Medicinal leech antimicrobial peptides lacking toxicity represent a promising alternative strategy to combat antibiotic-resistant pathogens. *European Journal of Medicinal Chemistry* **180**, 143-153 (2019).
422. H. Choi *et al.*, A human protein hydroxylase that accepts D-residues. *Communications Chemistry* **3**, 52 (2020).
423. A. M. Lencina *et al.*, Characterization and X-ray structure of the NADH-dependent coenzyme A disulfide reductase from Thermus thermophilus. *Biochimica et Biophysica Acta (BBA) - Bioenergetics* **1860**, 148080 (2019).
424. X. Chen *et al.*, The HIV-1 maturation inhibitor, EP39, interferes with the dynamic helix-coil equilibrium of the CA-SP1 junction of Gag. *European Journal of Medicinal Chemistry* **204**, 112634 (2020).
425. 6ryq. https://www.rcsb.org/structure/6RYQ.
426. 6rz1. https://www.rcsb.org/structure/6RZ1.

427. J. Day, A. Passecker, H.-P. Beck, I. Vakonakis, The Plasmodium falciparum Hsp70-x chaperone assists the heat stress response of the malaria parasite. *The FASEB Journal* **33**, 14611-14624 (2019).
428. S. Gihaz *et al.*, Bridges to Stability: Engineering Disulfide Bonds Towards Enhanced Lipase Biodiesel Synthesis. *ChemCatChem* **12**, 181-192 (2020).
429. J. Szczepaniak *et al.*, The lipoprotein Pal stabilises the bacterial outer membrane during constriction by a mobilisation-and-capture mechanism. *Nature Communications* **11**, 1305 (2020).
430. A. Maurer, F. Leisinger, D. Lim, F. P. Seebeck, Structure and Mechanism of Ergothionase from Treponema denticola. *Chemistry – A European Journal* **25**, 10298-10303 (2019).
431. B. Zhang *et al.*, Structure of a proton-dependent lipid transporter involved in lipoteichoic acids biosynthesis. *Nature Structural & Molecular Biology* **27**, 561-569 (2020).
432. S. Reynaud *et al.*, A Venomics Approach Coupled to High-Throughput Toxin Production Strategies Identifies the First Venom-Derived Melanocortin Receptor Agonists. *Journal of Medicinal Chemistry* **63**, 8250-8264 (2020).
433. O. Acton *et al.*, Structural basis for Fullerene geometry in a human endogenous retrovirus capsid. *Nature Communications* **10**, 5822 (2019).
434. M. Blueggel, J. van den Boom, H. Meyer, P. Bayer, C. Beuck, Structure of the PUB Domain from Ubiquitin Regulatory X Domain Protein 1 (UBXD1) and Its Interaction with the p97 AAA+ ATPase. *Biomolecules* **9**, 876 (2019).
435. V. Kuban *et al.*, Structural Basis of Ca²⁺-Dependent Self-Processing Activity of Repeat-in-Toxin Proteins. *mBio* **11**, 10.1128/mbio.00226-00220 (2020).
436. E. Madland, O. Crasson, M. Vandevenne, M. Sørlie, F. L. Aachmann, NMR and Fluorescence Spectroscopies Reveal the Preorganized Binding Site in Family 14 Carbohydrate-Binding Module from Human Chitotriosidase. *ACS Omega* **4**, 21975-21984 (2019).
437. 6so8. https://www.rcsb.org/structure/6SO8.
438. T. Gutmann *et al.*, Cryo-EM structure of the complete and ligand-saturated insulin receptor ectodomain. *Journal of Cell Biology* **219**, (2019).
439. 6sxw. https://www.rcsb.org/structure/6SXW.
440. M. D. Allen, M. Bycroft, G. Zinzalla, Structure of the BRK domain of the SWI/SNF chromatin remodeling complex subunit BRG1 reveals a potential role in protein–protein interactions. *Protein Science* **29**, 1033-1039 (2020).
441. A. Ben-Bassat, M. Giladi, Y. Haitin, Structure of KCNH2 cyclic nucleotide-binding homology domain reveals a functionally vital salt-bridge. *Journal of General Physiology* **152**, (2020).
442. S. Van Belle *et al.*, Unlike its Paralog LEDGF/p75, HRP-2 Is Dispensable for MLL-R Leukemogenesis but Important for Leukemic Cell Survival. *Cells* **10**, 192 (2021).
443. L. K. Lerner *et al.*, Timeless couples G-quadruplex detection with processing by DDX11 helicase during DNA replication. *The EMBO Journal* **39**, e104185 (2020).
444. S. J. Muderspach *et al.*, Further structural studies of the lytic polysaccharide monooxygenase AoAA13 belonging to the starch-active AA13 family. *Amylase* **3**, 41-54 (2019).

445. F. Risser *et al.*, Towards improved understanding of intersubunit interactions in modular polyketide biosynthesis: Docking in the enacyloxin IIa polyketide synthase. *Journal of Structural Biology* **212**, 107581 (2020).
446. 6tey. https://www.rcsb.org/structure/6TEY.
447. S. Toledo-Patiño, M. Chaubey, M. Coles, B. Höcker, Reconstructing the Remote Origins of a Fold Singleton from a Flavodoxin-Like Ancestor. *Biochemistry* **58**, 4790-4793 (2019).
448. F. Beilstein *et al.*, Identification of a pH-Sensitive Switch in VSV-G and a Crystal Structure of the G Pre-fusion State Highlight the VSV-G Structural Transition Pathway. *Cell Reports* **32**, 108042 (2020).
449. 6tk9. https://www.rcsb.org/structure/6TK9.
450. B. K. Hansen *et al.*, Structure and Function of the Bacterial Protein Toxin Phenomycin. *Structure* **28**, 528-539.e529 (2020).
451. H. Crawley-Snowdon *et al.*, Mechanism and evolution of the Zn-fingernail required for interaction of VARP with VPS29. *Nature Communications* **11**, 5031 (2020).
452. C. Manatschal *et al.*, Mechanistic basis of the inhibition of SLC11/NRAMP-mediated metal ion transport by bis-isothiourea substituted compounds. *eLife* **8**, e51913 (2019).
453. G. Javitt *et al.*, Assembly Mechanism of Mucin and von Willebrand Factor Polymers. *Cell* **183**, 717-729.e716 (2020).
454. N. T. Odermatt *et al.*, Structural and DNA binding properties of mycobacterial integration host factor mIHF. *Journal of Structural Biology* **209**, 107434 (2020).
455. H.-S. Kang *et al.*, An autoinhibitory intramolecular interaction proof-reads RNA recognition by the essential splicing factor U2AF2. *Proceedings of the National Academy of Sciences* **117**, 7140-7149 (2020).
456. J. Watzel, C. Hacker, E. Duchardt-Ferner, H. B. Bode, J. Wöhnert, A New Docking Domain Type in the Peptide-Antimicrobial-Xenorhabdus Peptide Producing Nonribosomal Peptide Synthetase from Xenorhabdus bovienii. *ACS Chemical Biology* **15**, 982-989 (2020).
457. C. Seuring *et al.*, The three-dimensional structure of human β-endorphin amyloid fibrils. *Nature Structural & Molecular Biology* **27**, 1178-1184 (2020).
458. J. A. Rodriguez Buitrago, T. Klunemann, W. Blankenfeldt, A. Schallmey, Expression, purification and crystal structure determination of a ferredoxin reductase from the actinobacterium Thermobifida fusca. *Acta Crystallographica Section F* **76**, 334-340 (2020).
459. A. K. Tarafder *et al.*, Phage liquid crystalline droplets form occlusive sheaths that encapsulate and protect infectious rod-shaped bacteria. *Proceedings of the National Academy of Sciences* **117**, 4724-4731 (2020).
460. V. Lux *et al.*, Molecular Mechanism of LEDGF/p75 Dimerization. *Structure* **28**, 1288-1299.e1287 (2020).
461. F. Cantini *et al.*, Effect of positive charges in the structural interaction of crabrolin isoforms with lipopolysaccharide. *Journal of Peptide Science* **26**, e3271 (2020).
462. D. Sheppard *et al.*, The major subunit of widespread competence pili exhibits a novel and conserved type IV pilin fold. *Journal of Biological Chemistry* **295**, 6594-6604 (2020).
463. K. Manne, S. V. L. Narayana, Structural insights into the role of the N-terminus in the activation and function of extracellular serine protease from Staphylococcus epidermidis. *Acta Crystallographica Section D* **76**, 28-40 (2020).

464. T. Nguyen *et al.*, The role of 9-O-acetylated glycan receptor moieties in the typhoid toxin binding and intoxication. *PLOS Pathogens* **16**, e1008336 (2020).
465. G. R. Buel *et al.*, Structure of E3 ligase E6AP with a proteasome-binding site provided by substrate receptor hRpn10. *Nature Communications* **11**, 1291 (2020).
466. E. Harjes *et al.*, Experimentally based structural model of Yih1 provides insight into its function in controlling the key translational regulator Gcn2. *FEBS Letters* **595**, 324-340 (2021).
467. 6u1s. https://www.rcsb.org/structure/6U1S.
468. A. M. White *et al.*, Application and Structural Analysis of Triazole-Bridged Disulfide Mimetics in Cyclic Peptides. *Angewandte Chemie International Edition* **59**, 11273-11277 (2020).
469. S. Das *et al.*, Structural Organization and Dynamics of Homodimeric Cytohesin Family Arf GTPase Exchange Factors in Solution and on Membranes. *Structure* **27**, 1782-1797.e1787 (2019).
470. T. K. Karamanos, V. Tugarinov, G. M. Clore, Unraveling the structure and dynamics of the human DNAJB6b chaperone by NMR reveals insights into Hsp40-mediated proteostasis. *Proceedings of the National Academy of Sciences* **116**, 21529-21538 (2019).
471. 6u46. https://www.rcsb.org/structure/6U46.
472. K. L. Stern *et al.*, Context-Dependent Stabilizing Interactions among Solvent-Exposed Residues along the Surface of a Trimeric Helix Bundle. *Biochemistry* **59**, 1672-1679 (2020).
473. M. Cai *et al.*, Probing transient excited states of the bacterial cell division regulator MinE by relaxation dispersion NMR spectroscopy. *Proceedings of the National Academy of Sciences* **116**, 25446-25455 (2019).
474. A. M. Valencia *et al.*, Recurrent SMARCB1 Mutations Reveal a Nucleosome Acidic Patch Interaction Site That Potentiates mSWI/SNF Complex Chromatin Remodeling. *Cell* **179**, 1342-1356.e1323 (2019).
475. G. W. Buchko, S. N. Hewitt, A. J. Napuli, W. C. Van Voorhis, P. J. Myler, Backbone and side chain 1H, 13C, and 15N NMR assignments for the organic hydroperoxide resistance protein (Ohr) from Burkholderia pseudomallei. *Biomolecular NMR Assignments* **3**, 163-166 (2009).
476. V. Ramanujam, Y. Shen, J. Ying, M. Mobli, Residual Dipolar Couplings for Resolving Cysteine Bridges in Disulfide-Rich Peptides. *Frontiers in Chemistry* **7**, (2020).
477. 6urs. https://www.rcsb.org/structure/6URS.
478. B. Krishnarjuna *et al.*, A disulfide-stabilised helical hairpin fold in acrorhagin I: An emerging structural motif in peptide toxins. *Journal of Structural Biology* **213**, 107692 (2021).
479. T. Xiao *et al.*, HIV-1 fusion inhibitors targeting the membrane-proximal external region of Env spikes. *Nature Chemical Biology* **16**, 529-537 (2020).
480. F. Maiello *et al.*, Crystal structure of Thermus thermophilus methylenetetrahydrofolate dehydrogenase and determinants of thermostability. *PLOS ONE* **15**, e0232959 (2020).
481. H. W. Kim *et al.*, Skp1 Dimerization Conceals Its F-Box Protein Binding Site. *Biochemistry* **59**, 1527-1536 (2020).
482. M. T. Nguyen *et al.*, An effective human uracil-DNA glycosylase inhibitor targets the open pre-catalytic active site conformation. *Progress in Biophysics and Molecular Biology* **163**, 143-159 (2021).

483. M. Tauber *et al.*, Alternative splicing and allosteric regulation modulate the chromatin binding of UHRF1. *Nucleic Acids Research* **48**, 7728-7747 (2020).
484. E. K. Gilding *et al.*, Neurotoxic peptides from the venom of the giant Australian stinging tree. *Science Advances* **6**, eabb8828 (2020).
485. S. C. Bobeica, L. Zhu, J. Z. Acedo, W. Tang, W. A. van der Donk, Structural determinants of macrocyclization in substrate-controlled lanthipeptide biosynthetic pathways. *Chemical Science* **11**, 12854-12870 (2020).
486. Y. Gu *et al.*, Structure of Geobacter pili reveals secretory rather than nanowire behaviour. *Nature* **597**, 430-434 (2021).
487. G. H. Mariano *et al.*, Characterization of novel human intragenic antimicrobial peptides, incorporation and release studies from ureasil-polyether hybrid matrix. *Materials Science and Engineering: C* **119**, 111581 (2021).
488. 6vrj. https://www.rcsb.org/structure/6VRJ.
489. H.-H. Wu, J. Symersky, M. Lu, Structure and mechanism of a redesigned multidrug transporter from the Major Facilitator Superfamily. *Scientific Reports* **10**, 3949 (2020).
490. Y. Wei *et al.*, The MYC oncoprotein directly interacts with its chromatin cofactor PNUTS to recruit PP1 phosphatase. *Nucleic Acids Research* **50**, 3505-3522 (2022).
491. F. Sesterhenn *et al.*, De novo protein design enables the precise induction of RSV-neutralizing antibodies. *Science* **368**, eaay5051 (2020).
492. B. P. Medellin *et al.*, Structural Basis for the Asymmetry of a 4-Oxalocrotonate Tautomerase Trimer. *Biochemistry* **59**, 1592-1603 (2020).
493. B. J. Payliss *et al.*, Phosphorylation of the DNA repair scaffold SLX4 drives folding of the SAP domain and activation of the MUS81-EME1 endonuclease. *Cell Reports* **41**, 111537 (2022).
494. A. E. López-Giraldo *et al.*, The three-dimensional structure of the toxic peptide Cl13 from the scorpion Centruroides limpidus. *Toxicon* **184**, 158-166 (2020).
495. 6vy7. https://www.rcsb.org/structure/6VY7.
496. L. A. Powell *et al.*, Human mAbs Broadly Protect against Arthritogenic Alphaviruses by Recognizing Conserved Elements of the Mxra8 Receptor-Binding Site. *Cell Host & Microbe* **28**, 699-711.e697 (2020).
497. H. Turano *et al.*, Molecular Structure and Functional Analysis of Pyocin S8 from Pseudomonas aeruginosa Reveals the Essential Requirement of a Glutamate Residue in the H-N-H Motif for DNase Activity. *Journal of Bacteriology* **202**, 10.1128/jb.00346-00320 (2020).
498. E. F. Reinhart *et al.*, A highly conserved glutamic acid in ALFY inhibits membrane binding to aid in aggregate clearance. *Traffic* **22**, 23-37 (2021).
499. A. R. Kaplan, R. Olson, A. T. Alexandrescu, Protein yoga: Conformational versatility of the Hemolysin II C-terminal domain detailed by NMR structures for multiple states. *Protein Science* **30**, 990-1005 (2021).
500. 6wbo. https://www.rcsb.org/structure/6WBO.
501. 6wbu. https://www.rcsb.org/structure/6WBU.
502. 6wc6. https://www.rcsb.org/structure/6WC6.
503. M. A. Outram *et al.*, The crystal structure of SnTox3 from the necrotrophic fungus Parastagonospora nodorum reveals a unique effector fold and provides insight into Snn3 recognition and pro-domain protease processing of fungal effectors. *New Phytologist* **231**, 2282-2296 (2021).

504. M.-C. Gor *et al.*, Crystal structure and site-directed mutagenesis of circular bacteriocin plantacyclin B21AG reveals cationic and aromatic residues important for antimicrobial activity. *Scientific Reports* **10**, 17398 (2020).
505. B. M. Hayes *et al.*, Ticks Resist Skin Commensals with Immune Factor of Bacterial Origin. *Cell* **183**, 1562-1571.e1512 (2020).
506. F. Wang *et al.*, Structural analysis of cross α-helical nanotubes provides insight into the designability of filamentous peptide nanomaterials. *Nature Communications* **12**, 407 (2021).
507. I. P. Gomes *et al.*, Membrane interactions of the anuran antimicrobial peptide HSP1-NH2: Different aspects of the association to anionic and zwitterionic biomimetic systems. *Biochimica et Biophysica Acta (BBA) - Biomembranes* **1863**, 183449 (2021).
508. C. D. Payne *et al.*, Defining the Familial Fold of the Vicilin-Buried Peptide Family. *Journal of Natural Products* **83**, 3030-3040 (2020).
509. J. Liu *et al.*, Structural Insight into Binding of the ZZ Domain of HERC2 to Histone H3 and SUMO1. *Structure* **28**, 1225-1230.e1223 (2020).
510. I. Chen *et al.*, Glutamate transporters have a chloride channel with two hydrophobic gates. *Nature* **591**, 327-331 (2021).
511. K. P. Smith *et al.*, Solution structure and dynamics of the mitochondrial-targeted GTPase-activating protein (GAP) VopE by an integrated NMR/SAXS approach. *Protein Science* **31**, e4282 (2022).
512. P. Ryzhov *et al.*, Conformational States of the Cytoprotective Protein Bcl-xL. *Biophysical Journal* **119**, 1324-1334 (2020).
513. K. L. McMahon *et al.*, Discovery, Pharmacological Characterisation and NMR Structure of the Novel μ-Conotoxin SxIIIC, a Potent and Irreversible NaV Channel Inhibitor. *Biomedicines* **8**, 391 (2020).
514. 6xeh. https://www.rcsb.org/structure/6XEH.
515. 6xmn. https://www.rcsb.org/structure/6XMN.
516. L. R. H. Krumpe *et al.*, Recifin A, Initial Example of the Tyr-Lock Peptide Structural Family, Is a Selective Allosteric Inhibitor of Tyrosyl-DNA Phosphodiesterase I. *Journal of the American Chemical Society* **142**, 21178-21188 (2020).
517. N. M. Loening, E. Barbar, Structural characterization of the self-association domain of swallow. *Protein Science* **30**, 1056-1063 (2021).
518. A. Liu *et al.*, Functional elucidation of TfuA in peptide backbone thioamidation. *Nature Chemical Biology* **17**, 585-592 (2021).
519. C. T. Nordyke, Y. M. Ahmed, R. Z. Puterbaugh, G. R. Bowman, K. Varga, Intrinsically Disordered Bacterial Polar Organizing Protein Z, PopZ, Interacts with Protein Binding Partners Through an N-terminal Molecular Recognition Feature. *Journal of Molecular Biology* **432**, 6092-6107 (2020).
520. Y. Hsia *et al.*, Design of multi-scale protein complexes by hierarchical building block fusion. *Nature Communications* **12**, 2294 (2021).
521. V. Poignavent *et al.*, A Flexible and Original Architecture of Two Unrelated Zinc Fingers Underlies the Role of the Multitask P1 in RYMV Spread. *Journal of Molecular Biology* **434**, 167715 (2022).
522. A. Neuhaus *et al.*, Cryo-electron microscopy reveals two distinct type IV pili assembled by the same bacterium. *Nature Communications* **11**, 2231 (2020).
523. 6xyi. https://www.rcsb.org/structure/6XYI.

524. I. B. Trindade, M. Invernici, F. Cantini, R. O. Louro, M. Piccioli, PRE-driven protein NMR structures: an alternative approach in highly paramagnetic systems. *The FEBS Journal* **288**, 3010-3023 (2021).
525. S. Baeriswyl *et al.*, A mixed chirality α-helix in a stapled bicyclic and a linear antimicrobial peptide revealed by X-ray crystallography. *RSC Chemical Biology* **2**, 1608-1617 (2021).
526. N. M. Hollmann *et al.*, Pseudo-RNA-Binding Domains Mediate RNA Structure Specificity in Upstream of N-Ras. *Cell Reports* **32**, 107930 (2020).
527. A. Kumar, C. Planchais, R. Fronzes, H. Mouquet, N. Reyes, Binding mechanisms of therapeutic antibodies to human CD20. *Science* **369**, 793-799 (2020).
528. 6ydh. https://www.rcsb.org/structure/6YDH.
529. P. Rathner *et al.*, Interhelical interactions within the STIM1 CC1 domain modulate CRAC channel activation. *Nature Chemical Biology* **17**, 196-204 (2021).
530. J. Bogomolovas *et al.*, Titin kinase ubiquitination aligns autophagy receptors with mechanical signals in the sarcomere. *EMBO reports* **22**, e48018 (2021).
531. M. Silber, M. Hitzenberger, M. Zacharias, C. Muhle-Goll, Altered Hinge Conformations in APP Transmembrane Helix Mutants May Affect Enzyme–Substrate Interactions of γ-Secretase. *ACS Chemical Neuroscience* **11**, 4426-4433 (2020).
532. A. A. Kawale, B. M. Burmann, UvrD helicase–RNA polymerase interactions are governed by UvrD's carboxy-terminal Tudor domain. *Communications Biology* **3**, 607 (2020).
533. C. Kelly, N. Pace, M. Gage, M. Pfuhl, Solution NMR Structure of Titin N2A Region Ig Domain I83 and Its Interaction with Metal Ions. *Journal of Molecular Biology* **433**, 166977 (2021).
534. J. Berndtsson *et al.*, Respiratory supercomplexes enhance electron transport by decreasing cytochrome c diffusion distance. *EMBO reports* **21**, e51015 (2020).
535. A. Grinzato *et al.*, High-Light versus Low-Light: Effects on Paired Photosystem II Supercomplex Structural Rearrangement in Pea Plants. *International Journal of Molecular Sciences* **21**, 8643 (2020).
536. D. Aparicio *et al.*, Structure and mechanism of the Nap adhesion complex from the human pathogen Mycoplasma genitalium. *Nature Communications* **11**, 2877 (2020).
537. 6yrr. https://www.rcsb.org/structure/6YRR.
538. F. B. Bdira *et al.*, Novel anti-repression mechanism of H-NS proteins by a phage protein. *Nucleic Acids Research* **49**, 10770-10784 (2021).
539. G. Stalmans *et al.*, Addressing the Molecular Mechanism of Longitudinal Lamin Assembly Using Chimeric Fusions. *Cells* **9**, 1633 (2020).
540. D. A. Gray *et al.*, Insights into SusCD-mediated glycan import by a prominent gut symbiont. *Nature Communications* **12**, 44 (2021).
541. F. Paoletti *et al.*, Endogenous modulators of neurotrophin signaling: Landscape of the transient ATP-NGF interactions. *Computational and Structural Biotechnology Journal* **19**, 2938-2949 (2021).
542. L. Radamaker *et al.*, Cryo-EM reveals structural breaks in a patient-derived amyloid fibril from systemic AL amyloidosis. *Nature Communications* **12**, 875 (2021).
543. B. Chaves-Arquero *et al.*, eIF4G1 N-terminal intrinsically disordered domain is a multi-docking station for RNA, Pab1, Pub1, and self-assembly. *Frontiers in Molecular Biosciences* **9**, (2022).

544. E. Madland *et al.*, Structural and functional variation of chitin-binding domains of a lytic polysaccharide monooxygenase from Cellvibrio japonicus. *Journal of Biological Chemistry* **297**, 101084 (2021).
545. O. Faust *et al.*, HSP40 proteins use class-specific regulation to drive HSP70 functional diversity. *Nature* **587**, 489-494 (2020).
546. L. M. Sjøgaard-Frich *et al.*, Dynamic Na+/H+ exchanger 1 (NHE1) – calmodulin complexes of varying stoichiometry and structure regulate Ca2+-dependent NHE1 activation. *eLife* **10**, e60889 (2021).
547. 6zfv. https://www.rcsb.org/structure/6ZFV.
548. E. S. Cunha, X. Chen, M. Sanz-Gaitero, D. J. Mills, H. Luecke, Cryo-EM structure of Helicobacter pylori urease with an inhibitor in the active site at 2.0 Å resolution. *Nature Communications* **12**, 230 (2021).
549. M. T. Bueno-Carrasco *et al.*, Structural mechanism for tyrosine hydroxylase inhibition by dopamine and reactivation by Ser40 phosphorylation. *Nature Communications* **13**, 74 (2022).
550. M. Pazos *et al.*, SPOR Proteins Are Required for Functionality of Class A Penicillin-Binding Proteins in Escherichia coli. *mBio* **11**, 10.1128/mbio.02796-02720 (2020).
551. K. Cermakova *et al.*, A ubiquitous disordered protein interaction module orchestrates transcription elongation. *Science* **374**, 1113-1121 (2021).
552. B. Bersch, N. Tarbouriech, W. P. Burmeister, F. Iseni, Solution Structure of the C-terminal Domain of A20, the Missing Brick for the Characterization of the Interface between Vaccinia Virus DNA Polymerase and its Processivity Factor. *Journal of Molecular Biology* **433**, 167009 (2021).
553. 6zyg. https://www.rcsb.org/structure/6ZYG.
554. 7aaf. https://www.rcsb.org/structure/7AAF.
555. 7aao. https://www.rcsb.org/structure/7AAO.
556. A. Marcelot *et al.*, Di-phosphorylated BAF shows altered structural dynamics and binding to DNA, but interacts with its nuclear envelope partners. *Nucleic Acids Research* **49**, 3841-3855 (2021).
557. F. Friis Theisen *et al.*, αα-hub coregulator structure and flexibility determine transcription factor binding and selection in regulatory interactomes. *Journal of Biological Chemistry* **298**, 101963 (2022).
558. I. A. Bolosov *et al.*, Dodecapeptide Cathelicidins of Cetartiodactyla: Structure, Mechanism of Antimicrobial Action, and Synergistic Interaction With Other Cathelicidins. *Frontiers in Microbiology* **12**, (2021).
559. P. Lanzoni-Mangutchi *et al.*, Structure and assembly of the S-layer in C. difficile. *Nature Communications* **13**, 970 (2022).
560. A. Schedlbauer *et al.*, A conserved rRNA switch is central to decoding site maturation on the small ribosomal subunit. *Science Advances* **7**, eabf7547 (2021).
561. P. V. Panteleev *et al.*, Structure Elucidation and Functional Studies of a Novel β-hairpin Antimicrobial Peptide from the Marine Polychaeta Capitella teleta. *Marine Drugs* **18**, 620 (2020).
562. C. Spitz *et al.*, Non-canonical Shedding of TNFα by SPPL2a Is Determined by the Conformational Flexibility of Its Transmembrane Helix. *iScience* **23**, 101775 (2020).

563. O. Tőke *et al.*, Solution NMR Structure of the SH3 Domain of Human Caskin1 Validates the Lack of a Typical Peptide Binding Groove and Supports a Role in Lipid Mediator Binding. *Cells* **10**, 173 (2021).
564. Y. Korolkova *et al.*, New Insectotoxin from Tibellus Oblongus Spider Venom Presents Novel Adaptation of ICK Fold. *Toxins* **13**, 29 (2021).
565. X. Xiao *et al.*, Selective Aster inhibitors distinguish vesicular and nonvesicular sterol transport mechanisms. *Proceedings of the National Academy of Sciences* **118**, e2024149118 (2021).
566. P. Pettersson *et al.*, Structure and dynamics of plant TatA in micelles and lipid bilayers studied by solution NMR. *The FEBS Journal* **285**, 1886-1906 (2018).
567. J. P. Wurm, K.-A. Glowacz, R. Sprangers, Structural basis for the activation of the DEAD-box RNA helicase DbpA by the nascent ribosome. *Proceedings of the National Academy of Sciences* **118**, e2105961118 (2021).
568. N. Leneva, O. Kovtun, D. R. Morado, J. A. G. Briggs, D. J. Owen, Architecture and mechanism of metazoan retromer:SNX3 tubular coat assembly. *Science Advances* **7**, eabf8598 (2021).
569. S. Minami *et al.*, Exploration of novel αβ-protein folds through de novo design. *Nature Structural & Molecular Biology* **30**, 1132-1140 (2023).
570. 7bqn. https://www.rcsb.org/structure/7BQN.
571. 7bqq. https://www.rcsb.org/structure/7BQQ.
572. 7bqr. https://www.rcsb.org/structure/7BQR.
573. 7bqs. https://www.rcsb.org/structure/7BQS.
574. H. Furihata *et al.*, Structural bases of IMiD selectivity that emerges by 5-hydroxythalidomide. *Nature Communications* **11**, 4578 (2020).
575. H. L. Chun, S. Y. Lee, S. H. Lee, C. S. Lee, H. H. Park, Enzymatic reaction mechanism of cis-aconitate decarboxylase based on the crystal structure of IRG1 from Bacillus subtilis. *Scientific Reports* **10**, 11305 (2020).
576. N. Nakayama *et al.*, Nucleus Accumbens-Associated Protein 1 Binds DNA Directly through the BEN Domain in a Sequence-Specific Manner. *Biomedicines* **8**, 608 (2020).
577. D. Heymann *et al.*, Structure of a consensus chitin-binding domain revealed by solution NMR. *Journal of Structural Biology* **213**, 107725 (2021).
578. G. Pandit *et al.*, Effect of Secondary Structure and Side Chain Length of Hydrophobic Amino Acid Residues on the Antimicrobial Activity and Toxicity of 14-Residue-Long de novo AMPs. *ChemMedChem* **16**, 355-367 (2021).
579. 7bxy. https://www.rcsb.org/structure/7BXY.
580. P. Zhang *et al.*, Bacteriophage protein Gp46 is a cross-species inhibitor of nucleoid-associated HU proteins. *Proceedings of the National Academy of Sciences* **119**, e2116278119 (2022).
581. F. Xu *et al.*, Structural and molecular basis for the substrate positioning mechanism of a new PL7 subfamily alginate lyase from the arctic. *Journal of Biological Chemistry* **295**, 16380-16392 (2020).
582. S. Boral, S. Maiti, A. J. Basak, W. Lee, S. De, Structural, Dynamic, and Functional Characterization of a DnaX Mini-intein Derived from Spirulina platensis Provides Important Insights into Intein-Mediated Catalysis of Protein Splicing. *Biochemistry* **59**, 4711-4724 (2020).

583. Y. Yamada *et al.*, Fragment-Based Discovery of Novel Non-Hydroxamate LpxC Inhibitors with Antibacterial Activity. *Journal of Medicinal Chemistry* **63**, 14805-14820 (2020).
584. Y. Zhao *et al.*, Phosphosite-dependent presentation of dual phosphorylated peptides by MHC class I molecules. *iScience* **25**, 104013 (2022).
585. 7cix. https://www.rcsb.org/structure/7CIX.
586. N. Iwakawa *et al.*, Transient Diffusive Interactions with a Protein Crowder Affect Aggregation Processes of Superoxide Dismutase 1 β-Barrel. *The Journal of Physical Chemistry B* **125**, 2521-2532 (2021).
587. K. Komatsu *et al.*, Identification of a Proline-Kinked Amphipathic α-Helix Downstream from the Methyltransferase Domain of a Potexvirus Replicase and Its Role in Virus Replication and Perinuclear Complex Formation. *Journal of Virology* **95**, 10.1128/jvi.01906-01920 (2021).
588. N. Isozumi *et al.*, Structure and antimicrobial activity of NCR169, a nodule-specific cysteine-rich peptide of Medicago truncatula. *Scientific Reports* **11**, 9923 (2021).
589. J. W. Barnett *et al.*, Spontaneous drying of non-polar deep-cavity cavitand pockets in aqueous solution. *Nature Chemistry* **12**, 589-594 (2020).
590. A. F. U. H. Saeed *et al.*, Structural Insights into gp16 ATPase in the Bacteriophage ϕ29 DNA Packaging Motor. *Biochemistry* **60**, 886-897 (2021).
591. 7cnf. https://www.rcsb.org/structure/7CNF.
592. Y. Yasutake *et al.*, Bacterial triacylglycerol lipase is a potential cholesterol esterase: Identification of a key determinant for sterol-binding specificity. *International Journal of Biological Macromolecules* **167**, 578-586 (2021).
593. W. Chen *et al.*, Calmodulin binds to Drosophila TRP with an unexpected mode. *Structure* **29**, 330-344.e334 (2021).
594. N. Zhang *et al.*, Structural basis of NF-κB signaling by the p75 neurotrophin receptor interaction with adaptor protein TRADD through their respective death domains. *Journal of Biological Chemistry* **297**, 100916 (2021).
595. S. Shimamoto *et al.*, Topological Regulation of the Bioactive Conformation of a Disulfide-Rich Peptide, Heat-Stable Enterotoxin. *Molecules* **25**, 4798 (2020).
596. 7cuz. https://www.rcsb.org/structure/7CUZ.
597. W. Lan *et al.*, Structural Basis of RACK7 PHD Domain to Read a Pediatric Glioblastoma-Associated Histone Mutation H3.3G34R. *Chinese Journal of Chemistry* **39**, 2433-2440 (2021).
598. S. Kapoor, A. Kodesia, N. Kalidas, Ashish, K. G. Thakur, Structural characterization of Myxococcus xanthus MglC, a component of the polarity control system, and its interactions with its paralog MglB. *Journal of Biological Chemistry* **296**, 100308 (2021).
599. Q. Shao *et al.*, Cryo-EM reveals a previously unrecognized structural protein of a dsRNA virus implicated in its extracellular transmission. *PLOS Pathogens* **17**, e1009396 (2021).
600. 7czj. https://www.rcsb.org/structure/7CZJ.
601. O.-S. Park *et al.*, Structure of neuroendocrine regulatory peptide-2 in membrane-mimicking environments. *Peptide Science* **113**, e24206 (2021).
602. Y. Chen *et al.*, Structure and mechanism of the [gamma]-glutamyl-[gamma]-aminobutyrate hydrolase SpuA from Pseudomonas aeruginosa. *Acta Crystallographica Section D* **77**, 1305-1316 (2021).
603. 7d5y. https://www.rcsb.org/structure/7D5Y.

604. J. Liu *et al.*, Cryo-EM structures of human calcium homeostasis modulator 5. *Cell Discovery* **6**, 81 (2020).
605. 7dab. https://www.rcsb.org/structure/7DAB.
606. Q. Wang *et al.*, Regulation of PD-L1 through direct binding of cholesterol to CRAC motifs. *Science Advances* **8**, eabq4722 (2022).
607. Z. Q. Bai, B. Liu, X. Ma, K. Hu, Backbone and side-chain chemical shift assignments of a cellular FLICE-inhibitory protein (c-FLIP(S)). *Biomol NMR Assign* **14**, 239-243 (2020).
608. T. Fan, Y. Zhang, J.-S. Fan, W. Yuan, Z. Lin, 1H, 15N and 13C resonance assignments of a repetitive domain of tubuliform spidroin 2. *Biomolecular NMR Assignments* **15**, 475-477 (2021).
609. S. Yagi *et al.*, Seven Amino Acid Types Suffice to Create the Core Fold of RNA Polymerase. *Journal of the American Chemical Society* **143**, 15998-16006 (2021).
610. 7dkr. https://www.rcsb.org/structure/7DKR.
611. C. Wang *et al.*, Molecular basis for substrate recognition by the bacterial nucleoside transporter NupG. *Journal of Biological Chemistry* **296**, 100479 (2021).
612. J. Ren *et al.*, Structural basis for the DNA-binding activity of human ARID4B Tudor domain. *Journal of Biological Chemistry* **296**, 100506 (2021).
613. H. Hu *et al.*, Aha1 Exhibits Distinctive Dynamics Behavior and Chaperone-Like Activity. *Molecules* **26**, 1943 (2021).
614. B. Huang *et al.*, A backbone-centred energy function of neural networks for protein design. *Nature* **602**, 523-528 (2022).
615. R. Kamiya *et al.*, Acid-stable capsid structure of Helicobacter pylori bacteriophage KHP30 by single-particle cryoelectron microscopy. *Structure* **30**, 300-312.e303 (2022).
616. H. Gao *et al.*, Nicking mechanism underlying the DNA phosphorothioate-sensing antiphage defense by SspE. *Nature Communications* **13**, 6773 (2022).
617. Y. Dong *et al.*, Structure and mechanism of the human NHE1-CHP1 complex. *Nature Communications* **12**, 3474 (2021).
618. C. Dou *et al.*, Crystal structure and catalytic mechanism of the MbnBC holoenzyme required for methanobactin biosynthesis. *Cell Research* **32**, 302-314 (2022).
619. 7e4j. https://www.rcsb.org/structure/7E4J.
620. B. Pandey *et al.*, Insights on the disruption of the complex between human positive coactivator 4 and p53 by small molecules. *Biochemical and Biophysical Research Communications* **578**, 15-20 (2021).
621. T. Yoshimi *et al.*, Crystal structure of thermally stable homodimeric cytochrome c'-[beta] from Thermus thermophilus. *Acta Crystallographica Section F* **78**, 217-225 (2022).
622. R. Zhang *et al.*, Fungal effector SIB1 of Colletotrichum orbiculare has unique structural features and can suppress plant immunity in Nicotiana benthamiana. *Journal of Biological Chemistry* **297**, 101370 (2021).
623. 7edk. https://www.rcsb.org/structure/7EDK.
624. J.-T. Zhang *et al.*, Structure and assembly pattern of a freshwater short-tailed cyanophage Pam1. *Structure* **30**, 240-251.e244 (2022).
625. 7eg5. https://www.rcsb.org/structure/7EG5.
626. 7egr. https://www.rcsb.org/structure/7EGR.
627. 7elk. https://www.rcsb.org/structure/7ELK.
628. J. Zha *et al.*, An evolution-inspired strategy to design disulfide-rich peptides tolerant to extensive sequence manipulation. *Chemical Science* **12**, 11464-11472 (2021).

629. X. Yan *et al.*, Molecular basis for recognition of Gly/N-degrons by CRL2ZYG11B and CRL2ZER1. *Molecular Cell* **81**, 3262-3274.e3263 (2021).
630. X. Wang *et al.*, Structural and biochemical analyses of the tetrameric carboxypeptidase S9Cfn from Fusobacterium nucleatum. *Acta Crystallographica Section D* **77**, 1554-1563 (2021).
631. 7eqm. https://www.rcsb.org/structure/7EQM.
632. Y. Oda *et al.*, Structural insights into the enhanced thermostability of cysteine substitution mutants of L-histidine decarboxylase from Photobacterium phosphoreum. *The Journal of Biochemistry* **171**, 31-40 (2021).
633. 7evq. https://www.rcsb.org/structure/7EVQ.
634. 7ewh. https://www.rcsb.org/structure/7EWH.
635. D. Liu *et al.*, O-Glycosylation Induces Amyloid-β To Form New Fibril Polymorphs Vulnerable for Degradation. *Journal of the American Chemical Society* **143**, 20216-20223 (2021).
636. Y. Xia *et al.*, Nγ-Hydroxyasparagine: A Multifunctional Unnatural Amino Acid That is a Good P1 Substrate of Asparaginyl Peptide Ligases. *Angewandte Chemie International Edition* **60**, 22207-22211 (2021).
637. Q. Zhai *et al.*, DNA binding mechanism of WhiB4 from Mycobacterium tuberculosis. *Magnetic Resonance Letters* **2**, 17-27 (2022).
638. L.-Y. Qin, Z. Gong, K. Liu, X. Dong, C. Tang, Kinetic Constraints in the Specific Interaction between Phosphorylated Ubiquitin and Proteasomal Shuttle Factors. *Biomolecules* **11**, 1008 (2021).
639. R. K. Yadav, V. Krishnan, New structural insights into the PI-2 pilus from Streptococcus oralis, an early dental plaque colonizer. *The FEBS Journal* **289**, 6342-6366 (2022).
640. F. He *et al.*, 1H, 13C and 15N resonance assignments and solution structures of the two RRM domains of Matrin-3. *Biomolecular NMR Assignments* **16**, 41-49 (2022).
641. J. Lan *et al.*, Structural insights into the binding of SARS-CoV-2, SARS-CoV, and hCoV-NL63 spike receptor-binding domain to horse ACE2. *Structure* **30**, 1432-1442.e1434 (2022).
642. F. Cai, I. M. Robertson, T. Kampourakis, B. A. Klein, B. D. Sykes, The Role of Electrostatics in the Mechanism of Cardiac Thin Filament Based Sensitizers. *ACS Chemical Biology* **15**, 2289-2298 (2020).
643. J. A. I. Muller *et al.*, Antimicrobial and Anticancer Properties of Synthetic Peptides Derived from the Wasp Parachartergus fraternus. *ChemBioChem* **22**, 1415-1423 (2021).
644. A. F. Dishman *et al.*, Evolution of fold switching in a metamorphic protein. *Science* **371**, 86-90 (2021).
645. F. Pirro *et al.*, Allosteric cooperation in a de novo-designed two-domain protein. *Proceedings of the National Academy of Sciences* **117**, 33246-33253 (2020).
646. R. Takjoo *et al.*, Folding of Truncated Granulin Peptides. *Biomolecules* **10**, 1152 (2020).
647. M. R. Bleackley *et al.*, Histidine-Rich Defensins from the Solanaceae and Brasicaceae Are Antifungal and Metal Binding Proteins. *Journal of Fungi* **6**, 145 (2020).
648. 7jpm. https://www.rcsb.org/structure/7JPM.
649. S. Aiyer *et al.*, A common binding motif in the ET domain of BRD3 forms polymorphic structural interfaces with host and viral proteins. *Structure* **29**, 886-898.e886 (2021).

650. L. A. Harris *et al.*, Reactivity-Based Screening for Citrulline-Containing Natural Products Reveals a Family of Bacterial Peptidyl Arginine Deiminases. *ACS Chemical Biology* **15**, 3167-3175 (2020).
651. A. E. Sears *et al.*, Single particle cryo-EM of the complex between interphotoreceptor retinoid-binding protein and a monoclonal antibody. *The FASEB Journal* **34**, 13918-13934 (2020).
652. L. Friedrich *et al.*, Learning from Nature: From a Marine Natural Product to Synthetic Cyclooxygenase-1 Inhibitors by Automated De Novo Design. *Advanced Science* **8**, 2100832 (2021).
653. C. A. Schmidt *et al.*, Identification and Characterization of a Peptide from the Stony Coral Heliofungia actiniformis. *Journal of Natural Products* **83**, 3454-3463 (2020).
654. V. S. Mandala *et al.*, Structure and drug binding of the SARS-CoV-2 envelope protein transmembrane domain in lipid bilayers. *Nature Structural & Molecular Biology* **27**, 1202-1208 (2020).
655. 7k3s. *https://www.rcsb.org/structure/7K3S*.
656. Y. Wang *et al.*, Calcium binds and rigidifies the dysferlin C2A domain in a tightly coupled manner. *Biochemical Journal* **478**, 197-215 (2021).
657. L. Zhao, Q. Fu, L. Pan, A. Piai, J. J. Chou, The Diversity and Similarity of Transmembrane Trimerization of TNF Receptors. *Frontiers in cell and developmental biology* **8**, 569684 (2020).
658. S. A. McConnell *et al.*, Sortase-assembled pili in <i>Corynebacterium diphtheriae</i> are built using a latch mechanism. *Proceedings of the National Academy of Sciences* **118**, e2019649118 (2021).
659. T. Nguyen *et al.*, The structural basis of Salmonella A2B5 toxin neutralization by antibodies targeting the glycan-receptor binding subunits. *Cell Reports* **36**, 109654 (2021).
660. 7kbq. *https://www.rcsb.org/structure/7KBQ*.
661. O. Kern *et al.*, The structures of two salivary proteins from the West Nile vector Culex quinquefasciatus reveal a beta-trefoil fold with putative sugar binding properties. *Current Research in Structural Biology* **3**, 95-105 (2021).
662. 7kdq. *https://www.rcsb.org/structure/7KDQ*.
663. L. Robert Hollingsworth *et al.*, Mechanism of filament formation in UPA-promoted CARD8 and NLRP1 inflammasomes. *Nature Communications* **12**, 189 (2021).
664. J. Azadmanesh, W. E. Lutz, L. Coates, K. L. Weiss, G. E. O. Borgstahl, Direct detection of coupled proton and electron transfers in human manganese superoxide dismutase. *Nature Communications* **12**, 2079 (2021).
665. W. W. Aragaw *et al.*, Potency boost of a <i>Mycobacterium tuberculosis</i> dihydrofolate reductase inhibitor by multienzyme F₄₂₀H₂-dependent reduction. *Proceedings of the National Academy of Sciences* **118**, e2025172118 (2021).
666. J. E. Longbotham, M. J. S. Kelly, D. G. Fujimori, Recognition of Histone H3 Methylation States by the PHD1 Domain of Histone Demethylase KDM5A. *ACS Chemical Biology*, (2021).
667. W. Lee, R. O. Frederick, M. Tonelli, A. C. Palmenberg, Solution NMR Determination of the CDHR3 Rhinovirus-C Binding Domain, EC1. *Viruses* **13**, 159 (2021).

668. J. M. Singer *et al.*, Large-scale design and refinement of stable proteins using sequence-only models. *PLOS ONE* **17**, e0265020 (2022).
669. 7kwi. https://www.rcsb.org/structure/7KWI.
670. M. Ultsch *et al.*, Structures of the ApoL1 and ApoL2 N-terminal domains reveal a non-classical four-helix bundle motif. *Communications Biology* **4**, 916 (2021).
671. A. Padilla *et al.*, Conus venom fractions inhibit the adhesion of Plasmodium falciparum erythrocyte membrane protein 1 domains to the host vascular receptors. *Journal of Proteomics* **234**, 104083 (2021).
672. K. H. Adel, H. B. Mohammed, A. Boris, Z. Jianqin, F. P. Sébastien, NMR solution structure and analysis of isolated S3b-S4a motif of repeat IV of the human cardiac sodium channel. *bioRxiv*, 2021.2005.2021.443843 (2021).
673. 7l8v. https://www.rcsb.org/structure/7L8V.
674. Q. Fu, J. J. Chou, A Trimeric Hydrophobic Zipper Mediates the Intramembrane Assembly of SARS-CoV-2 Spike. *Journal of the American Chemical Society* **143**, 8543-8546 (2021).
675. L. Cao *et al.*, Cellulonodin-2 and Lihuanodin: Lasso Peptides with an Aspartimide Post-Translational Modification. *Journal of the American Chemical Society* **143**, 11690-11702 (2021).
676. T. W. Linsky *et al.*, Sampling of structure and sequence space of small protein folds. *Nature Communications* **13**, 7151 (2022).
677. 7lgl. https://www.rcsb.org/structure/7LGL.
678. 7lie. https://www.rcsb.org/structure/7LIE.
679. H. E. Elashal *et al.*, Biosynthesis and characterization of fuscimiditide, an aspartimidylated graspetide. *Nature Chemistry* **14**, 1325-1334 (2022).
680. N. Kozlyuk *et al.*, A fragment-based approach to discovery of Receptor for Advanced Glycation End products inhibitors. *Proteins: Structure, Function, and Bioinformatics* **89**, 1399-1412 (2021).
681. L. Rheinemann *et al.*, Interactions between AMOT PPxY motifs and NEDD4L WW domains function in HIV-1 release. *Journal of Biological Chemistry* **297**, 100975 (2021).
682. Y. Gu *et al.*, Structure of Geobacter cytochrome OmcZ identifies mechanism of nanowire assembly and conductivity. *Nature Microbiology* **8**, 284-298 (2023).
683. J. Giribaldi *et al.*, Synthesis, Structural and Pharmacological Characterizations of CIC, a Novel α-Conotoxin with an Extended N-Terminal Tail. *Marine Drugs* **19**, 141 (2021).
684. 7lrw. https://www.rcsb.org/structure/7LRW.
685. 7lsp. https://www.rcsb.org/structure/7LSP.
686. 7lt7. https://www.rcsb.org/structure/7LT7.
687. E.-M. E. Uhlemann, W. Lee, M. Tonelli, O. Y. Dmitriev, At sixes and sevens: cryptic domain in the metal binding chain of the human copper transporter ATP7A. *Biophysical Journal* **120**, 4600-4607 (2021).
688. 7luw. https://www.rcsb.org/structure/7LUW.
689. A. C. Y. Foo *et al.*, Structure, Immunogenicity, and IgE Cross-Reactivity among Walnut and Peanut Vicilin-Buried Peptides. *Journal of Agricultural and Food Chemistry* **70**, 2389-2400 (2022).
690. 7lwr. https://www.rcsb.org/structure/7LWR.
691. 7lx4. https://www.rcsb.org/structure/7LX4.
692. 7lzl. https://www.rcsb.org/structure/7LZL.

693. J. Kraus *et al.*, Magic angle spinning NMR structure of human cofilin-2 assembled on actin filaments reveals isoform-specific conformation and binding mode. *Nature Communications* **13**, 2114 (2022).
694. E. López-Giraldo *et al.*, Structural and functional studies of scorpine: A channel blocker and cytolytic peptide. *Toxicon* **222**, 106985 (2023).
695. D. Herrmann *et al.*, Structural Insights into the Mechanism of Human T-cell Leukemia Virus Type 1 Gag Targeting to the Plasma Membrane for Assembly. *Journal of Molecular Biology* **433**, 167161 (2021).
696. B.-J. Baas *et al.*, Kinetic and Structural Analysis of Two Linkers in the Tautomerase Superfamily: Analysis and Implications. *Biochemistry* **60**, 1776-1786 (2021).
697. I. Anishchenko *et al.*, De novo protein design by deep network hallucination. *Nature* **600**, 547-552 (2021).
698. B. P. O. Santos *et al.*, Schistocins: Novel antimicrobial peptides encrypted in the Schistosoma mansoni Kunitz Inhibitor SmKI-1. *Biochimica et Biophysica Acta (BBA) - General Subjects* **1865**, 129989 (2021).
699. B. Ruan *et al.*, Design and characterization of a protein fold switching network. *Nature Communications* **14**, 431 (2023).
700. B. J. LaFrance *et al.*, The encapsulin from Thermotoga maritima is a flavoprotein with a symmetry matched ferritin-like cargo protein. *Scientific Reports* **11**, 22810 (2021).
701. G. U. Oka *et al.*, Structural basis for effector recognition by an antibacterial type IV secretion system. *Proceedings of the National Academy of Sciences* **119**, e2112529119 (2022).
702. R. K. Koppisetti, Y. G. Fulcher, S. R. Van Doren, Fusion Peptide of SARS-CoV-2 Spike Rearranges into a Wedge Inserted in Bilayered Micelles. *Journal of the American Chemical Society* **143**, 13205-13211 (2021).
703. A. V. Reshetnyak *et al.*, Mechanism for the activation of the anaplastic lymphoma kinase receptor. *Nature* **600**, 153-157 (2021).
704. O. Kgosisejo, J. A. Chen, P. Grochulski, T. Tanaka, Crystallographic structure of recombinant Lactococcus lactis prolidase to support proposed structure-function relationships. *Biochimica et Biophysica Acta (BBA) - Proteins and Proteomics* **1865**, 473-480 (2017).
705. N. R. Rutbeek, H. Rezasoltani, T. R. Patel, M. Khajehpour, G. Prehna, Molecular mechanism of quorum sensing inhibition in Streptococcus by the phage protein paratox. *Journal of Biological Chemistry* **297**, 100992 (2021).
706. S. Nawarathnage *et al.*, Crystals of TELSAM–target protein fusions that exhibit minimal crystal contacts and lack direct inter-TELSAM contacts. *Open Biology* **12**, 210271 (2022).
707. T. N. T. Ho *et al.*, Posttranslational modifications of α-conotoxins: sulfotyrosine and C-terminal amidation stabilise structures and increase acetylcholine receptor binding. *RSC Medicinal Chemistry* **12**, 1574-1584 (2021).
708. Y. J. Huang *et al.*, Assessment of prediction methods for protein structures determined by NMR in CASP14: Impact of AlphaFold2. *Proteins: Structure, Function, and Bioinformatics* **89**, 1959-1976 (2021).
709. S. Bhattacharya, A. Palillo, Structural and dynamic studies of the peptidase domain from Clostridium thermocellum PCAT1. *Protein Science* **31**, 498-512 (2022).

710. A. S. Weinheimer *et al.*, Extended DNA-binding interfaces beyond the canonical SAP domain contribute to the function of replication stress regulator SDE2 at DNA replication forks. *Journal of Biological Chemistry* **298**, 102268 (2022).
711. S. S. Denisov *et al.*, Molecular basis of anticoagulant and anticomplement activity of the tick salivary protein Salp14 and its homologs. *Journal of Biological Chemistry* **297**, 100865 (2021).
712. A.-P. Schaffner *et al.*, Phosphinanes and Azaphosphinanes as Potent and Selective Inhibitors of Activated Thrombin-Activatable Fibrinolysis Inhibitor (TAFIa). *Journal of Medicinal Chemistry* **64**, 3897-3910 (2021).
713. 7nqa. https://www.rcsb.org/structure/7NQA.
714. S. Nonin-Lecomte, L. Fermon, B. Felden, M.-L. Pinel-Marie, Bacterial Type I Toxins: Folding and Membrane Interactions. *Toxins* **13**, 490 (2021).
715. I. Rosa e Silva *et al.*, Molecular mechanisms underlying the role of the centriolar CEP164-TTBK2 complex in ciliopathies. *Structure* **30**, 114-128.e119 (2022).
716. A. Czajlik *et al.*, DMSO-Induced Unfolding of the Antifungal Disulfide Protein PAF and Its Inactive Variant: A Combined NMR and DSC Study. *International Journal of Molecular Sciences* **24**, 1208 (2023).
717. J. Skokowa *et al.*, A topological refactoring design strategy yields highly stable granulopoietic proteins. *Nature Communications* **13**, 2948 (2022).
718. M. A. Dubinnyi, P. V. Dubovskii, V. G. Starkov, Y. N. Utkin, The omega-loop of cobra cytotoxins tolerates multiple amino acid substitutions. *Biochemical and Biophysical Research Communications* **558**, 141-146 (2021).
719. M. Fiedler *et al.*, Head-to-tail polymerization by VEL proteins underpins cold-induced Polycomb silencing in flowering control. *Cell Reports* **41**, 111607 (2022).
720. S. Violot *et al.*, Exploring molecular determinants of polysaccharide lyase family 6–1 enzyme activity. *Glycobiology* **31**, 1557-1570 (2021).
721. S. Brüschweiler *et al.*, A Step toward NRF2-DNA Interaction Inhibitors by Fragment-Based NMR Methods. *ChemMedChem* **16**, 3576-3587 (2021).
722. 7od2. https://www.rcsb.org/structure/7OD2.
723. L. E. Sperl, F. Rührnößl, A. Schiller, M. Haslbeck, F. Hagn, High-resolution analysis of the conformational transition of pro-apoptotic Bak at the lipid membrane. *The EMBO Journal* **40**, e107159 (2021).
724. 7oio. https://www.rcsb.org/structure/7OIO.
725. H. Tossavainen *et al.*, Structure of SNX9 SH3 in complex with a viral ligand reveals the molecular basis of its unique specificity for alanine-containing class I SH3 motifs. *Structure* **30**, 828-839.e826 (2022).
726. 7olg. https://www.rcsb.org/structure/7OLG.
727. A. Huskova *et al.*, Model of abasic site DNA cross-link repair; from the architecture of NEIL3 DNA binding domains to the X-structure model. *Nucleic Acids Research* **50**, 10436-10448 (2022).
728. M. I. Sánchez-Ruiz *et al.*, Agaricales Mushroom Lignin Peroxidase: From Structure–Function to Degradative Capabilities. *Antioxidants* **10**, 1446 (2021).
729. M. Narczyk *et al.*, Interactions of 2,6-substituted purines with purine nucleoside phosphorylase from Helicobacter pylori in solution and in the crystal, and the effects of these compounds on cell cultures of this bacterium. *Journal of Enzyme Inhibition and Medicinal Chemistry* **37**, 1083-1097 (2022).

730. N. Wesch, F. Löhr, N. Rogova, V. Dötsch, V. V. Rogov, A Concerted Action of UBA5 C-Terminal Unstructured Regions Is Important for Transfer of Activated UFM1 to UFC1. *International Journal of Molecular Sciences* **22**, 7390 (2021).
731. F. Altegoer *et al.*, Structural insights into the mechanism of archaellar rotational switching. *Nature Communications* **13**, 2857 (2022).
732. 7ovx. https://www.rcsb.org/structure/7OVX.
733. A. I. Kuzmenkov *et al.*, Apamin structure and pharmacology revisited. *Frontiers in Pharmacology* **13**, (2022).
734. 7oz0. https://www.rcsb.org/structure/7OZ0.
735. M. Birkou *et al.*, Impact of a Single Nucleotide Polymorphism on the 3D Protein Structure and Ubiquitination Activity of E3 Ubiquitin Ligase Arkadia. *Frontiers in Molecular Biosciences* **9**, (2022).
736. 7p2o. https://www.rcsb.org/structure/7P2O.
737. 7p3m. https://www.rcsb.org/structure/7P3M.
738. 7p3o. https://www.rcsb.org/structure/7P3O.
739. 7p3p. https://www.rcsb.org/structure/7P3P.
740. P.-c. Chen *et al.*, Structure and dynamics of the von Willebrand Factor C6 domain. *Journal of Structural Biology* **214**, 107923 (2022).
741. F. Gorrec, D. Bellini, The FUSION protein crystallization screen. *Journal of Applied Crystallography* **55**, 310-319 (2022).
742. P. B. Timmons, C. M. Hewage, Conformation and membrane interaction studies of the potent antimicrobial and anticancer peptide palustrin-Ca. *Scientific Reports* **11**, 22468 (2021).
743. M. Buonocore *et al.*, Structural analysis of a simplified model reproducing SARS-CoV-2 S RBD/ACE2 binding site. *Heliyon* **8**, e11568 (2022).
744. 7pbh. https://www.rcsb.org/structure/7PBH.
745. V. Quèbre *et al.*, Characterization of the DNA Binding Domain of StbA, A Key Protein of A New Type of DNA Segregation System. *Journal of Molecular Biology* **434**, 167752 (2022).
746. 7ph8. https://www.rcsb.org/structure/7PH8.
747. 7pht. https://www.rcsb.org/structure/7PHT.
748. L. M. Bessa *et al.*, The intrinsically disordered SARS-CoV-2 nucleoprotein in dynamic complex with its viral partner nsp3a. *Science Advances* **8**, eabm4034 (2022).
749. 7pl4. https://www.rcsb.org/structure/7PL4.
750. 7pmp. https://www.rcsb.org/structure/7PMP.
751. B. Dorgan *et al.*, Structural Model of a Porphyromonas gingivalis type IX Secretion System Shuttle Complex. *Journal of Molecular Biology* **434**, 167871 (2022).
752. Z. F. Brotzakis *et al.*, A Structural Ensemble of a Tau-Microtubule Complex Reveals Regulatory Tau Phosphorylation and Acetylation Mechanisms. *ACS Central Science* **7**, 1986-1995 (2021).
753. K. Loth *et al.*, Aphid BCR4 Structure and Activity Uncover a New Defensin Peptide Superfamily. *International Journal of Molecular Sciences* **23**, 12480 (2022).
754. C.-A. Belén *et al.*, Structural basis of Nrd1–Nab3 heterodimerization. *Life Science Alliance* **5**, e202101252 (2022).
755. A. S. Paramonov, E. N. Lyukmanova, A. G. Tonevitsky, A. S. Arseniev, Z. O. Shenkarev, Spatial structure and oligomerization of viscotoxin A3 in detergent micelles:

Implication for mechanisms of ion channel formation and membrane lysis. *Biochemical and Biophysical Research Communications* **585**, 22-28 (2021).
756. C. C. Torres Cabán *et al.*, Tuning the Sensitivity of Genetically Encoded Fluorescent Potassium Indicators through Structure-Guided and Genome Mining Strategies. *ACS Sensors* **7**, 1336-1346 (2022).
757. J. H. Overbeck, D. Stelzig, A.-L. Fuchs, J. P. Wurm, R. Sprangers, Observation of conformational changes that underlie the catalytic cycle of Xrn2. *Nature Chemical Biology* **18**, 1152-1160 (2022).
758. 7pzt. https://www.rcsb.org/structure/7PZT.
759. O. Mikhailovskii, Y. Xue, N. R. Skrynnikov, Modeling a unit cell: crystallographic refinement procedure using the biomolecular MD simulation platform Amber. *IUCrJ* **9**, 114-133 (2022).
760. A. Ibáñez de Opakua *et al.*, Molecular interactions of FG nucleoporin repeats at high resolution. *Nature Chemistry* **14**, 1278-1285 (2022).
761. M. A. Morando *et al.*, Solution structure of recombinant Pvfp-5β reveals insights into mussel adhesion. *Communications Biology* **5**, 739 (2022).
762. V. Siebert, M. Silber, E. Heuten, C. Muhle-Goll, M. K. Lemberg, Cleavage of mitochondrial homeostasis regulator PGAM5 by the intramembrane protease PARL is governed by transmembrane helix dynamics and oligomeric state. *Journal of Biological Chemistry* **298**, 102321 (2022).
763. 7qb0. https://www.rcsb.org/structure/7QB0.
764. S. S. Mariasina *et al.*, Williams-Beuren Syndrome Related Methyltransferase WBSCR27: From Structure to Possible Function. *Frontiers in Molecular Biosciences* **9**, (2022).
765. M.-E. Chagot, A. Boutilliat, A. Kriznik, M. Quinternet, Structural Analysis of the Plasmodial Proteins ZNHIT3 and NUFIP1 Provides Insights into the Selectivity of a Conserved Interaction. *Biochemistry* **61**, 479-493 (2022).
766. H. A. Heikkinen, A. S. Aranko, H. Iwaï, The NMR structure of the engineered halophilic DnaE intein for segmental isotopic labeling using conditional protein splicing. *Journal of Magnetic Resonance* **338**, 107195 (2022).
767. 7qjf. https://www.rcsb.org/structure/7QJF.
768. P. Martin-Malpartida *et al.*, Conformational ensemble of the TNF-derived peptide solnatide in solution. *Computational and Structural Biotechnology Journal* **20**, 2082-2090 (2022).
769. R. Bücker *et al.*, The Cryo-EM structures of two amphibian antimicrobial cross-β amyloid fibrils. *Nature Communications* **13**, 4356 (2022).
770. A. M. Gigolaev *et al.*, Artificial pore blocker acts specifically on voltage-gated potassium channel isoform KV1.6. *Journal of Biological Chemistry* **298**, 102467 (2022).
771. E. N. Grafskaia *et al.*, Non-toxic antimicrobial peptide Hm-AMP2 from leech metagenome proteins identified by the gradient-boosting approach. *Materials & Design* **224**, 111364 (2022).
772. O. Arnolds, R. Stoll, Characterization of a fold in TANGO1 evolved from SH3 domains for the export of bulky cargos. *Nature Communications* **14**, 2273 (2023).
773. 7r7m. https://www.rcsb.org/structure/7R7M.
774. S. Sarkar *et al.*, Structural basis of HIV-1 maturation inhibitor binding and activity. *Nature Communications* **14**, 1237 (2023).

775. K. D. Reddy, D. Ciftci, A. J. Scopelliti, O. Boudker, The archaeal glutamate transporter homologue GltPh shows heterogeneous substrate binding. *Journal of General Physiology* **154**, (2022).
776. J. P. Hallinan *et al.*, Design of functionalised circular tandem repeat proteins with longer repeat topologies and enhanced subunit contact surfaces. *Communications Biology* **4**, 1240 (2021).
777. R. Hellinger *et al.*, Importance of the Cyclic Cystine Knot Structural Motif for Immunosuppressive Effects of Cyclotides. *ACS Chemical Biology* **16**, 2373-2386 (2021).
778. H. Yin *et al.*, Rational Design of Potent Peptide Inhibitors of the PD-1:PD-L1 Interaction for Cancer Immunotherapy. *Journal of the American Chemical Society* **143**, 18536-18547 (2021).
779. W. Wang *et al.*, Cryo-EM structure of the sodium-driven chloride/bicarbonate exchanger NDCBE. *Nature Communications* **12**, 5690 (2021).
780. 7rul. https://www.rcsb.org/structure/7RUL.
781. F. Wang, O. Gnewou, A. Solemanifar, V. P. Conticello, E. H. Egelman, Cryo-EM of Helical Polymers. *Chemical Reviews* **122**, 14055-14065 (2022).
782. S. H. Mishra *et al.*, Global protein dynamics as communication sensors in peptide synthetase domains. *Science Advances* **8**, eabn6549 (2022).
783. M. J. Maxwell *et al.*, A bivalent remipede toxin promotes calcium release via ryanodine receptor activation. *Nature Communications* **14**, 1036 (2023).
784. K. N. Kraichely *et al.*, Minimal Increments of Hydrophobic Collapse within the N-Terminus of the Neuropeptide Galanin. *Biochemistry* **61**, 1151-1166 (2022).
785. T. T. Dang *et al.*, Mutagenesis of cyclotide Cter 27 exemplifies a robust folding strategy for bracelet cyclotides. *Peptide Science* **114**, e24284 (2022).
786. S. Lu *et al.*, An active site loop toggles between conformations to control antibiotic hydrolysis and inhibition potency for CTX-M β-lactamase drug-resistance enzymes. *Nature Communications* **13**, 6726 (2022).
787. Y. Xu *et al.*, The Ephb2 Receptor Uses Homotypic, Head-to-Tail Interactions within Its Ectodomain as an Autoinhibitory Control Mechanism. *International Journal of Molecular Sciences* **22**, 10473 (2021).
788. K. Steffen *et al.*, Barrettides: A Peptide Family Specifically Produced by the Deep-Sea Sponge Geodia barretti. *Journal of Natural Products* **84**, 3138-3146 (2021).
789. D. J. Shon, D. Fernandez, N. M. Riley, M. J. Ferracane, C. R. Bertozzi, Structure-guided mutagenesis of a mucin-selective metalloprotease from Akkermansia muciniphila alters substrate preferences. *Journal of Biological Chemistry* **298**, 101917 (2022).
790. A. Weiss *et al.*, Zn-regulated GTPase metalloprotein activator 1 modulates vertebrate zinc homeostasis. *Cell* **185**, 2148-2163.e2127 (2022).
791. T. D. Sudasinghe, M. T. Banco, D. R. Ronning, Inhibitors of Mycobacterium tuberculosis EgtD target both substrate binding sites to limit hercynine production. *Scientific Reports* **11**, 22240 (2021).
792. 7sft. https://www.rcsb.org/structure/7SFT.
793. R. J. Malonis *et al.*, A Powassan virus domain III nanoparticle immunogen elicits neutralizing and protective antibodies in mice. *PLOS Pathogens* **18**, e1010573 (2022).
794. 7siy. https://www.rcsb.org/structure/7SIY.

795. N. A. Khaje *et al.*, Validated determination of NRG1 Ig-like domain structure by mass spectrometry coupled with computational modeling. *Communications Biology* **5**, 452 (2022).
796. Y. Dongol *et al.*, Voltage-Gated Sodium Channel Modulation by a New Spider Toxin Ssp1a Isolated From an Australian Theraphosid. *Frontiers in Pharmacology* **12**, (2021).
797. M. W. Martynowycz, M. T. B. Clabbers, J. Hattne, T. Gonen, Ab initio phasing macromolecular structures using electron-counted MicroED data. *Nature Methods* **19**, 724-729 (2022).
798. A. A. Melo *et al.*, Cryo-electron tomography reveals structural insights into the membrane remodeling mode of dynamin-like EHD filaments. *Nature Communications* **13**, 7641 (2022).
799. L. Poppe, J. J. Hartman, A. Romero, J. D. Reagan, Structural and Thermodynamic Model for the Activation of Cardiac Troponin. *Biochemistry* **61**, 741-748 (2022).
800. A. Mukundan *et al.*, Convergent evolution of a parasite-encoded complement control protein-scaffold to mimic binding of mammalian TGF-β to its receptors, TβRI and TβRII. *Journal of Biological Chemistry* **298**, 101994 (2022).
801. R. D. Marcum *et al.*, A capped Tudor domain within a core subunit of the Sin3L/Rpd3L histone deacetylase complex binds to nucleic acid G-quadruplexes. *Journal of Biological Chemistry* **298**, 101558 (2022).
802. 7t2e. https://www.rcsb.org/structure/7T2E.
803. T.-E. Kim *et al.*, Dissecting the stability determinants of a challenging de novo protein fold using massively parallel design and experimentation. *Proceedings of the National Academy of Sciences* **119**, e2122676119 (2022).
804. R. D. Miller *et al.*, Computational identification of a systemic antibiotic for Gram-negative bacteria. *Nature Microbiology* **7**, 1661-1672 (2022).
805. C. Cobos *et al.*, Peptides derived from hookworm anti-inflammatory proteins suppress inducible colitis in mice and inflammatory cytokine production by human cells. *Frontiers in Medicine* **9**, (2022).
806. 7t7w. https://www.rcsb.org/structure/7T7W.
807. P. Feng *et al.*, A peroxisomal ubiquitin ligase complex forms a retrotranslocation channel. *Nature* **607**, 374-380 (2022).
808. M. N. Campos *et al.*, Solution NMR structure of cementum protein 1 derived peptide (CEMP1-p1) and its role in the mineralization process. *Journal of Peptide Science* **n/a**, e3494.
809. J. Zeller *et al.*, A novel phosphocholine-mimetic inhibits a pro-inflammatory conformational change in C-reactive protein. *EMBO Molecular Medicine* **15**, e16236 (2023).
810. 7tgp. https://www.rcsb.org/structure/7TGP.
811. 7tjg. https://www.rcsb.org/structure/7TJG.
812. 7tod. https://www.rcsb.org/structure/7TOD.
813. 7tt9. https://www.rcsb.org/structure/7TT9.
814. M. J. Espiritu *et al.*, Characterization of the Native Disulfide Isomers of the Novel χ-Conotoxin PnID: Implications for Further Increasing Conotoxin Diversity. *Marine Drugs* **21**, 61 (2023).
815. 7txx. https://www.rcsb.org/structure/7TXX.

816. R. Tejero, Y. J. Huang, T. A. Ramelot, G. T. Montelione, AlphaFold Models of Small Proteins Rival the Accuracy of Solution NMR Structures. *Frontiers in Molecular Biosciences* **9**, (2022).
817. A. Chang *et al.*, Homotypic fibrillization of TMEM106B across diverse neurodegenerative diseases. *Cell* **185**, 1346-1355.e1315 (2022).
818. G. L. J. Keller, L. I. Weiss, B. M. Baker, Physicochemical Heuristics for Identifying High Fidelity, Near-Native Structural Models of Peptide/MHC Complexes. *Frontiers in Immunology* **13**, (2022).
819. K. Wu *et al.*, De novo design of modular peptide-binding proteins by superhelical matching. *Nature* **616**, 581-589 (2023).
820. 7ugc. https://www.rcsb.org/structure/7UGC.
821. J. L. Harman *et al.*, Evolution avoids a pathological stabilizing interaction in the immune protein S100A9. *Proceedings of the National Academy of Sciences* **119**, e2208029119 (2022).
822. G. I. Hallinan *et al.*, Cryo-EM structures of prion protein filaments from Gerstmann–Sträussler–Scheinker disease. *Acta Neuropathologica* **144**, 509-520 (2022).
823. R. Grinter *et al.*, Structural basis for bacterial energy extraction from atmospheric hydrogen. *Nature* **615**, 541-547 (2023).
824. A. C. Y. Foo *et al.*, Structure and IgE Cross-Reactivity among Cashew, Pistachio, Walnut, and Peanut Vicilin-Buried Peptides. *Journal of Agricultural and Food Chemistry* **71**, 2990-2998 (2023).
825. D. E. Kim *et al.*, De novo design of small beta barrel proteins. *Proceedings of the National Academy of Sciences* **120**, e2207974120 (2023).
826. 7v5e. https://www.rcsb.org/structure/7V5E.
827. 7v5f. https://www.rcsb.org/structure/7V5F.
828. H.-Y. Chang *et al.*, Structural insights into the substrate selectivity of α-oxoamine synthases from marine Vibrio sp. QWI-06. *Colloids and Surfaces B: Biointerfaces* **210**, 112224 (2022).
829. 7vbg. https://www.rcsb.org/structure/7VBG.
830. 7vd7. https://www.rcsb.org/structure/7VD7.
831. 7vh9. https://www.rcsb.org/structure/7VH9.
832. N. Sekiyama *et al.*, ALS mutations in the TIA-1 prion-like domain trigger highly condensed pathogenic structures. *Proceedings of the National Academy of Sciences* **119**, e2122523119 (2022).
833. X.-W. Zhang *et al.*, Neuroinflammation inhibition by small-molecule targeting USP7 noncatalytic domain for neurodegenerative disease therapy. *Science Advances* **8**, eabo0789 (2022).
834. 7viv. https://www.rcsb.org/structure/7VIV.
835. 7vma. https://www.rcsb.org/structure/7VMA.
836. X. Yu *et al.*, Structural and functional basis of the selectivity filter as a gate in human TRPM2 channel. *Cell Reports* **37**, 110025 (2021).
837. Q. Lu *et al.*, Lynamicin B is a Potential Pesticide by Acting as a Lepidoptera-Exclusive Chitinase Inhibitor. *Journal of Agricultural and Food Chemistry* **69**, 14086-14091 (2021).

838. S. Inouye *et al.*, Reverse mutants of the catalytic 19 kDa mutant protein (nanoKAZ/nanoLuc) from Oplophorus luciferase with coelenterazine as preferred substrate. *PLOS ONE* **17**, e0272992 (2022).
839. 7vtm. *https://www.rcsb.org/structure/7VTM*.
840. L. Liu, S. Zhou, Y. Deng, Rational Design of the Substrate Tunnel of β-Ketothiolase Reveals a Local Cationic Domain Modulated Rule that Improves the Efficiency of Claisen Condensation. *ACS Catalysis* **13**, 8183-8194 (2023).
841. 7vu3. *https://www.rcsb.org/structure/7VU3*.
842. H. Wu, R. Cao, M. Wen, H. Xue, B. OuYang, Structural characterization of a dimerization interface in the CD28 transmembrane domain. *Structure* **30**, 803-812.e805 (2022).
843. Y. Duan *et al.*, Anaerobic Hydroxyproline Degradation Involving C–N Cleavage by a Glycyl Radical Enzyme. *Journal of the American Chemical Society* **144**, 9715-9722 (2022).
844. B.-S. Jeong *et al.*, Computational design of a neutralizing antibody with picomolar binding affinity for all concerning SARS-CoV-2 variants. *mAbs* **14**, 2021601 (2022).
845. P. Lu *et al.*, Rosmarinic Acid and Sodium Citrate Have a Synergistic Bacteriostatic Effect against Vibrio Species by Inhibiting Iron Uptake. *International Journal of Molecular Sciences* **22**, 13010 (2021).
846. A. Siodmak *et al.*, Essential role of the CD docking motif of MPK4 in plant immunity, growth, and development. *New Phytologist* **239**, 1112-1126 (2023).
847. Y. Wu *et al.*, Structure-guided design of CPPC-paired disulfide-rich peptide libraries for ligand and drug discovery. *Chemical Science* **13**, 7780-7789 (2022).
848. H. Kohga *et al.*, Crystal structure of the lipid flippase MurJ in a “squeezed” form distinct from its inward- and outward-facing forms. *Structure* **30**, 1088-1097.e1083 (2022).
849. K. Kataoka *et al.*, A cryptic phosphate-binding pocket on the SPFH domain of human stomatin that regulates a novel fibril-like self-assembly. *Current Research in Structural Biology* **4**, 158-166 (2022).
850. 7wnz. *https://www.rcsb.org/structure/7WNZ*.
851. Y. Hou *et al.*, Structural mechanisms of calmodulin activation of Shigella effector OspC3 to ADP-riboxanate caspase-4/11 and block pyroptosis. *Nature Structural & Molecular Biology* **30**, 261-272 (2023).
852. X. Yang *et al.*, Cryo-EM structures reveal the activation and substrate recognition mechanism of human enteropeptidase. *Nature Communications* **13**, 6955 (2022).
853. X. Ma *et al.*, Cryo-EM structures of two human B cell receptor isotypes. *Science* **377**, 880-885 (2022).
854. L. Wang *et al.*, Selection and structural bases of potent broadly neutralizing antibodies from 3-dose vaccinees that are highly effective against diverse SARS-CoV-2 variants, including Omicron sublineages. *Cell Research* **32**, 691-694 (2022).
855. 7wwc. *https://www.rcsb.org/structure/7WWC*.
856. M. Kojima *et al.*, Engineering of an in-cell protein crystal for fastening a metastable conformation of a target miniprotein. *Biomaterials Science* **11**, 1350-1357 (2023).
857. J. Chen *et al.*, Molecular and Structural Basis of Receptor Binding and Signaling of a Fish Type I IFN with Three Disulfide Bonds. *The Journal of Immunology* **209**, 806-819 (2022).
858. 7x59. *https://www.rcsb.org/structure/7X59*.

859. Y. Ma *et al.*, Structural and functional insights into CST tethering in Tetrahymena thermophila telomerase. *Structure* **30**, 1565-1572.e1564 (2022).
860. M. A. Sobhy *et al.*, Cryo-electron structures of the extreme thermostable enzymes Sulfur Oxygenase Reductase and Lumazine Synthase. *PLOS ONE* **17**, e0275487 (2022).
861. X. Xie *et al.*, Crystal structure of a novel homodimeric d-allulose 3-epimerase from a Clostridia bacterium. *Acta Crystallographica Section D* **78**, 1180-1191 (2022).
862. 7x89. https://www.rcsb.org/structure/7X89.
863. K. Kuwasako *et al.*, 1H, 13C, and 15N resonance assignments and solution structures of the KH domain of human ribosome binding factor A, mtRbfA, involved in mitochondrial ribosome biogenesis. *Biomolecular NMR Assignments* **16**, 297-303 (2022).
864. 7xb6. https://www.rcsb.org/structure/7XB6.
865. 7xez. https://www.rcsb.org/structure/7XEZ.
866. 7xfg. https://www.rcsb.org/structure/7XFG.
867. 7xfs. https://www.rcsb.org/structure/7XFS.
868. K. Hata *et al.*, Structural basis for the unique multifaceted interaction of DPPA3 with the UHRF1 PHD finger. *Nucleic Acids Research* **50**, 12527-12542 (2022).
869. 7xhz. https://www.rcsb.org/structure/7XHZ.
870. A. K. Gaurav *et al.*, The RRM-mediated RNA binding activity in T. brucei RAP1 is essential for VSG monoallelic expression. *Nature Communications* **14**, 1576 (2023).
871. S. Suzuki *et al.*, Structural insight into the activation mechanism of MrgD with heterotrimeric Gi-protein revealed by cryo-EM. *Communications Biology* **5**, 707 (2022).
872. Y. Irie *et al.*, Structural basis of the 24B3 antibody against the toxic conformer of amyloid β with a turn at positions 22 and 23. *Biochemical and Biophysical Research Communications* **621**, 162-167 (2022).
873. E. H. L. Chen *et al.*, 2.2 Å Cryo-EM Tetra-Protofilament Structure of the Hamster Prion 108–144 Fibril Reveals an Ordered Water Channel in the Center. *Journal of the American Chemical Society* **144**, 13888-13894 (2022).
874. L. Wang *et al.*, Structural Insights into Mouse H-FABP. *Life* **12**, 1445 (2022).
875. M. Selinger *et al.*, Tick-borne encephalitis virus capsid protein induces translational shutoff as revealed by its structural–biological analysis. *Journal of Biological Chemistry* **298**, 102585 (2022).
876. 7ywr. https://www.rcsb.org/structure/7YWR.
877. A. A. Papadopoulou *et al.*, Helical stability of the GnTV transmembrane domain impacts on SPPL3 dependent cleavage. *Scientific Reports* **12**, 20987 (2022).
878. 7z3c. https://www.rcsb.org/structure/7Z3C.
879. 7zbs. https://www.rcsb.org/structure/7ZBS.
880. D. Linde *et al.*, Structural Characterization of Two Short Unspecific Peroxygenases: Two Different Dimeric Arrangements. *Antioxidants* **11**, 891 (2022).
881. M. Blatter *et al.*, RNA binding induces an allosteric switch in Cyp33 to repress MLL1-mediated transcription. *Science Advances* **9**, eadf5330 (2023).
882. X.-R. Chen *et al.*, Mechanisms by which small molecules of diverse chemotypes arrest Sec14 lipid transfer activity. *Journal of Biological Chemistry* **299**, 102861 (2023).
883. 7zok. https://www.rcsb.org/structure/7ZOK.
884. A. Naschberger *et al.*, Algal photosystem I dimer and high-resolution model of PSI-plastocyanin complex. *Nature Plants* **8**, 1191-1201 (2022).
885. 7zro. https://www.rcsb.org/structure/7ZRO.

886. 7zru. https://www.rcsb.org/structure/7ZRU.
887. V. U. Chukhutsina *et al.*, Light activation of Orange Carotenoid Protein reveals bicycle-pedal single-bond isomerization. *Nature Communications* **13**, 6420 (2022).
888. F. D. Andersen *et al.*, Triculamin: An Unusual Lasso Peptide with Potent Antimycobacterial Activity. *Journal of Natural Products* **85**, 1514-1521 (2022).
889. A. Kusova *et al.*, Structure of amyloidogenic PAP(85-120) peptide by high-resolution NMR spectroscopy. *Journal of Molecular Structure* **1253**, 132294 (2022).
890. N. Kejzar *et al.*, Cryo-EM structure of ssDNA bacteriophage ΦCjT23 provides insight into early virus evolution. *Nature Communications* **13**, 7478 (2022).
891. S. Collin *et al.*, Decrypting the programming of β-methylation in virginiamycin M biosynthesis. *Nature Communications* **14**, 1327 (2023).
892. T. Shirasaki *et al.*, Nonlytic cellular release of hepatitis A virus requires dual capsid recruitment of the ESCRT-associated Bro1 domain proteins HD-PTP and ALIX. *PLOS Pathogens* **18**, e1010543 (2022).
893. R. Dazzoni *et al.*, Structure and dynamic association of an assembly platform subcomplex of the bacterial type II secretion system. *Structure* **31**, 152-165.e157 (2023).
894. H. Singh *et al.*, Epigenetic CpG duplex marks probed by an evolved DNA reader via a well-tempered conformational plasticity. *Nucleic Acids Research* **51**, 6495-6506 (2023).
895. V. A. Lushpa *et al.*, Spatial Structure of NanoFAST in the Apo State and in Complex with its Fluorogen HBR-DOM2. *International Journal of Molecular Sciences* **23**, 11361 (2022).
896. F. D. Kornilov *et al.*, The architecture of transmembrane and cytoplasmic juxtamembrane regions of Toll-like receptors. *Nature Communications* **14**, 1503 (2023).
897. J. Bürgi *et al.*, Asymmetric horseshoe-like assembly of peroxisomal yeast oxalyl-CoA synthetase. *Biological Chemistry* **404**, 195-207 (2023).
898. C. Rouillon *et al.*, Antiviral signalling by a cyclic nucleotide activated CRISPR protease. *Nature* **614**, 168-174 (2023).
899. M. Bischetti *et al.*, Structural insights on the selective interaction of the histidine-rich piscidin antimicrobial peptide Of-Pis1 with membranes. *Biochimica et Biophysica Acta (BBA) - Biomembranes* **1865**, 184080 (2023).
900. R. Ouyang *et al.*, High-resolution reconstruction of a Jumbo-bacteriophage infecting capsulated bacteria using hyperbranched tail fibers. *Nature Communications* **13**, 7241 (2022).
901. 8boo. https://www.rcsb.org/structure/8BOO.
902. Q. Wu, M. G. Koliopoulos, K. Rittinger, B. Stieglitz, Structural basis for ubiquitylation by HOIL-1. *Frontiers in Molecular Biosciences* **9**, (2023).
903. F. D. Kornilov *et al.*, Structural basis for the ligand promiscuity of the neofunctionalized, carotenoid-binding fasciclin domain protein AstaP. *Communications Biology* **6**, 471 (2023).
904. 8c1c. https://www.rcsb.org/structure/8C1C.
905. M. Prolic-Kalinsek *et al.*, Structural basis of DNA binding by YdaT, a functional equivalent of the CII repressor in the cryptic prophage CP-933P from Escherichia coli O157:H7. *Acta Crystallographica Section D* **79**, 245-258 (2023).
906. J. Böhning *et al.*, Architecture of the biofilm-associated archaic Chaperone-Usher pilus CupE from Pseudomonas aeruginosa. *PLOS Pathogens* **19**, e1011177 (2023).

907. M. S. Dickinson, T. Miyazawa, R. S. McCool, A. T. Keatinge-Clay, Priming enzymes from the pikromycin synthase reveal how assembly-line ketosynthases catalyze carbon-carbon chemistry. *Structure* **30**, 1331-1339.e1333 (2022).
908. Y. C. Park, B. Reddy, N. Bavi, E. Perozo, J. D. Faraldo-Gómez, State-specific morphological deformations of the lipid bilayer explain mechanosensitive gating of MscS ion channels. *eLife* **12**, e81445 (2023).
909. L. C. Beltran *et al.*, Archaeal DNA-import apparatus is homologous to bacterial conjugation machinery. *Nature Communications* **14**, 666 (2023).
910. 8dh7. https://www.rcsb.org/structure/8DH7.
911. A. W. Kulczyk *et al.*, Cryo-EM reveals the molecular basis of laminin polymerization and LN-lamininopathies. *Nature Communications* **14**, 317 (2023).
912. G. Du *et al.*, Autoinhibitory structure of preligand association state implicates a new strategy to attain effective DR5 receptor activation. *Cell Research* **33**, 131-146 (2023).
913. M. Yin *et al.*, Evolution of nanobodies specific for BCL11A. *Proceedings of the National Academy of Sciences* **120**, e2218959120 (2023).
914. B. Choi, H. E. Elashal, L. Cao, A. J. Link, Mechanistic Analysis of the Biosynthesis of the Aspartimidylated Graspetide Amycolimiditide. *Journal of the American Chemical Society* **144**, 21628-21639 (2022).
915. 8ef4. https://www.rcsb.org/structure/8EF4.
916. M. H. Doran, J. L. Baker, T. Dahlberg, M. Andersson, E. Bullitt, Three structural solutions for bacterial adhesion pilus stability and superelasticity. *Structure* **31**, 529-540.e527 (2023).
917. L. Xiao, V. G. Magupalli, H. Wu, Cryo-EM structures of the active NLRP3 inflammasome disc. *Nature* **613**, 595-600 (2023).
918. 8eu7. https://www.rcsb.org/structure/8EU7.
919. B. Gao *et al.*, Functional evolution of scorpion venom peptides with an inhibitor cystine knot fold. *Bioscience Reports* **33**, (2013).
920. J. J. Hu *et al.*, Discovery, structure, and function of filamentous 3-methylcrotonyl-CoA carboxylase. *Structure* **31**, 100-110.e104 (2023).
921. Y. Guo *et al.*, Monomer and dimer structures of cytochrome bo3 ubiquinol oxidase from Escherichia coli. *Protein Science* **32**, e4616 (2023).
922. J. Giribaldi *et al.*, Pmu1a, a novel spider toxin with dual inhibitory activity at pain targets hNaV1.7 and hCaV3 voltage-gated channels. *The FEBS Journal* **290**, 3688-3702 (2023).
923. M. A. Casasanta *et al.*, Structural Insights of the SARS-CoV-2 Nucleocapsid Protein: Implications for the Inner-workings of Rapid Antigen Tests. *Microscopy and Microanalysis* **29**, 649-657 (2022).
924. X. Zhu *et al.*, Discovery, Characterization, and Engineering of LvIC, an α4/4-Conotoxin That Selectively Blocks Rat α6/α3β4 Nicotinic Acetylcholine Receptors. *Journal of Medicinal Chemistry* **66**, 2020-2031 (2023).
925. J.-H. Kim *et al.*, Crystal structure of [1,2,4]triazolo[4,3-b]pyridazine derivatives as BRD4 bromodomain inhibitors and structure–activity relationship study. *Scientific Reports* **13**, 10805 (2023).
926. N. A. Oktaviani, A. D. Malay, A. Matsugami, F. Hayashi, K. Numata, Unusual pKa Values Mediate the Self-Assembly of Spider Dragline Silk Proteins. *Biomacromolecules* **24**, 1604-1616 (2023).

927. C.-C. Kao, T.-L. Lin, C.-J. Lin, T.-S. Tseng, Deciphering Structure-Function Relationship Unveils Salt-Resistant Mode of Action of a Potent MRSA-Inhibiting Antimicrobial Peptide, RR14. *Journal of Bacteriology* **204**, e00312-00322 (2022).
928. 8gyx. https://www.rcsb.org/structure/8GYX.
929. O.-S. Park, J.-K. Bang, C. Cheong, Y.-H. Jeon, Structure of AQEE-30 of VGF Neuropeptide in Membrane-Mimicking Environments. *International Journal of Molecular Sciences* **23**, 13953 (2022).
930. 8h6r. https://www.rcsb.org/structure/8H6R.
931. L. Pang *et al.*, Structural Basis of Cysteine Ligase MshC Inhibition by Cysteinyl-Sulfonamides. *International Journal of Molecular Sciences* **23**, 15095 (2022).
932. S. Liu *et al.*, A dimer-monomer transition captured by the crystal structures of cyanobacterial apo flavodoxin. *Biochemical and Biophysical Research Communications* **639**, 134-141 (2023).
933. M. He *et al.*, Discovery of a cysteine-rich peptide with glycation modification from Achyranthes bidentata Blume. *Fitoterapia* **163**, 105338 (2022).
934. 8hz1. https://www.rcsb.org/structure/8HZ1.
935. 8hz2.
936. 8ib0. https://www.rcsb.org/structure/8IB0.
937. G. Zhang *et al.*, FTL004, an anti-CD38 mAb with negligible RBC binding and enhanced pro-apoptotic activity, is a novel candidate for treatments of multiple myeloma and non-Hodgkin lymphoma. *Journal of Hematology & Oncology* **15**, 177 (2022).
938. J. Ding, H.-W. Choe, J. Granzin, W. Saenger, Structure of ribonuclease T1 complexed with zinc(II) at 1.8 A resolution: a Zn2+.6H2O.carboxylate clathrate. *Acta Crystallographica Section B* **48**, 185-191 (1992).
939. M. J. Abraham *et al.*, GROMACS: High performance molecular simulations through multi-level parallelism from laptops to supercomputers. *SoftwareX* **1-2**, 19-25 (2015).
940. J. Huang *et al.*, CHARMM36m: an improved force field for folded and intrinsically disordered proteins. *Nature Methods* **14**, 71-73 (2017).
941. G. Bussi, D. Donadio, M. Parrinello, Canonical sampling through velocity rescaling. *The Journal of Chemical Physics* **126**, (2007).
942. H. J. C. Berendsen, J. P. M. Postma, W. F. van Gunsteren, A. DiNola, J. R. Haak, Molecular dynamics with coupling to an external bath. *The Journal of Chemical Physics* **81**, 3684-3690 (1984).
943. M. Parrinello, A. Rahman, Polymorphic transitions in single crystals: A new molecular dynamics method. *Journal of Applied Physics* **52**, 7182-7190 (1981).
944. P. P. Ewald, Die Berechnung optischer und elektrostatischer Gitterpotentiale. *Annalen der Physik* **369**, 253-287 (1921).
945. B. Hess, H. Bekker, H. J. C. Berendsen, J. G. E. M. Fraaije, LINCS: A linear constraint solver for molecular simulations. *Journal of Computational Chemistry* **18**, 1463-1472 (1997).
946. J. Ji, B. Carpentier, A. Chakraborty, S. Nangia, An Affordable Topography-Based Protocol for Assigning a Residue's Character on a Hydropathy (PARCH) Scale. *Journal of Chemical Theory and Computation*, (2023).
947. W. Humphrey, A. Dalke, K. Schulten, VMD: Visual molecular dynamics. *Journal of Molecular Graphics* **14**, 33-38 (1996).
948. G. V. Rossum, F. L. Drake, *Python 3 Reference Manual*. (CreateSpace, 2009).

949. F. Pedregosa *et al.*, Scikit-learn: Machine Learning in Python. *J. Mach. Learn. Res.* **12**, 2825–2830 (2011).
950. P. J. Rousseeuw, Silhouettes: A graphical aid to the interpretation and validation of cluster analysis. *Journal of Computational and Applied Mathematics* **20**, 53-65 (1987).
951. L. The PyMOL Molecular Graphics System version 2.0 (Schrödinger, 2015).
952. J. D. Hunter, Matplotlib: A 2D Graphics Environment. *Computing in Science & Engineering* **9**, 90-95 (2007).
953. M. L. Waskom, seaborn: statistical data visualization. *Journal of Open Source Software* **6**, 3021 (2021).
954. N. Michaud-Agrawal, E. J. Denning, T. B. Woolf, O. Beckstein, MDAnalysis: A toolkit for the analysis of molecular dynamics simulations. *Journal of Computational Chemistry* **32**, 2319-2327 (2011).
